# Ethanol-Induced Alterations in Placental and Fetal Cerebrocortical Annexin-A4 and Cerebral Cavernous Malformation Protein 3 Are Associated With Reductions in Fetal Cortical VEGF Receptor Binding and Microvascular Density

**DOI:** 10.3389/fnins.2020.00519

**Published:** 2020-06-03

**Authors:** Daniel D. Savage, Martina J. Rosenberg, Laurent Coquet, Morgan W. Porch, Nyika A. Allen, Christian Roux, Caroline Aligny, Thierry Jouenne, Bruno J. Gonzalez

**Affiliations:** ^1^Department of Neurosciences, School of Medicine, University of New Mexico, Albuquerque, NM, United States; ^2^UMR 6270, CNRS, Normandie University, UNIROUEN, Proteomic Facility PISSARO, Institute for Research and Innovation in Biomedicine, Rouen, France; ^3^Normandie University, UNIROUEN, INSERM U1245, Normandy Centre for Genomic and Personalized Medicine, Institute for Research and Innovation in Biomedicine, Rouen, France

**Keywords:** ethanol, placenta, cerebral cortex, cerebral cavernous malformation protein 3, annexin-A4, fetal alcohol spectrum disorder

## Abstract

Jegou et al. (2012) have reported prenatal alcohol exposure (PAE)-induced reductions of angiogenesis-related proteins in mouse placenta. These effects were associated with striking alterations in microvascular development in neonatal cerebral cortex. Here, we employed a rat model of moderate PAE to search for additional proteins whose placental and fetal cortical expression is altered by PAE, along with a subsequent examination of fetal cerebral cortical alterations associated with altered protein expression. Long-Evans rat dams voluntarily consumed either a 0 or 5% ethanol solution 4 h each day throughout gestation. Daily ethanol consumption, which resulted in a mean peak maternal serum ethanol concentration of 60.8 mg/dL, did not affect maternal weight gain, litter size, or placental or fetal body weight. On gestational day 20, rat placental: fetal units were removed by Caesarian section. Placental protein expression, analyzed by 2D-PAGE – tandem mass spectroscopy, identified a total of 1,117 protein spots, 20 of which were significantly altered by PAE. To date, 14 of these PAE-altered proteins have been identified. Western blotting confirmed the alterations of two of these placental proteins, namely, annexin-A4 (ANX-A4) and cerebral cavernous malformation protein 3 (CCM-3). Specifically, PAE elevated ANX-A4 and decreased CCM-3 in placenta. Subsequently, these two proteins were measured in fetal cerebral cortex, along with radiohistochemical studies of VEGF binding and histofluorescence studies of microvascular density in fetal cerebral cortex. PAE elevated ANX-A4 and decreased CCM-3 in fetal cerebral cortex, in a pattern similar to the alterations observed in placenta. Further, both VEGF receptor binding and microvascular density and orientation, measures that are sensitive to reduced CCM-3 expression in developing brain, were significantly reduced in the ventricular zone of fetal cerebral cortex. These results suggest that the expression angiogenesis-related proteins in placenta might serve as a biomarker of ethanol-induced alterations in microvascular development in fetal brain.

## Introduction

Moderate drinking during pregnancy can cause subtle, long-term cognitive impairments in affected offspring, even in the absence of the physical defects associated with Fetal Alcohol Syndrome (Hanson et al., 1978; Shaywitz et al., 1980; Abel, 1984; Conry, 1990; Streissguth et al., 1990). A large majority of children affected by prenatal alcohol exposure (PAE) present with no physical evidence of functional brain damage at birth. In such cases, the adverse neurobehavioral consequences associated with PAE may not be recognized until much later in development, if ever, thus diminishing the prospect for recognizing causal factors and delaying opportunities for earlier interventions (Chasnoff et al., 2015). As such, one critical challenge is the need to develop more sensitive and reliable methods for detecting the neurobiological consequences of drinking during pregnancy. In particular, there is a need to identify prognostic markers of PAE, easily assessed early in development, allowing for earlier identification of children at risk for longer-term adverse neurobehavioral outcomes.

Over the past decade, one experimental approach to address this challenge has been to identify novel biochemical species in biomarker tissues that are altered as a consequence of alcohol consumption during pregnancy. Various biochemical species and processes have been examined, some using high throughput screening procedures such as DNA methylation (Lussier et al., 2018), mRNA expression profiles (Rosenberg et al., 2010; Downing et al., 2012; Carter et al., 2018), miRNA expression profiles (Balaraman et al., 2014, 2016; Gardiner et al., 2016) and proteomic approaches (Datta et al., 2008; Ramadoss and Magness, 2012; Davis-Anderson et al., 2017). While these approaches have identified a large array of novel targets affected by drinking during pregnancy, to date, there have been very few efforts to link these PAE-induced alterations in a biomarker to specific downstream consequences in brain that could be directly associated with altered neurodevelopment and adverse behavioral consequences.

Jegou et al. (2012) have reported PAE-induced alterations in angiogenesis-related proteins, expressed both in placenta and in fetal cerebral cortex, whose actions are critical to microvascular development and function in the placenta (Herraiz et al., 2015), as well as brain (Won et al., 2013). In particular, daily intraperitoneal injections of 3 g/kg ethanol from gestational day 13 through Postnatal Day 1 reduced the expression of VEGFA and VEGFR1 mRNAs in the cortex of 2-day-old mice. These effects were accompanied by significant reductions in cerebral microvascular density and striking disruptions in the radial architecture of the cerebral microvascular network. Moreover, the altered cortical expression of VEGFR1 characterized in the brain of neonates exposed *in utero* to alcohol was associated with a significant reduction of placental expression of placental growth factor (PLGF), VEGFR1 and VEGFR2, suggesting the existence of a functional placenta-brain axis involved in the control of brain angiogenesis, which is impaired by PAE. Consistent with this hypothesis, a genetic knockout of PLGF expression mimicked the protein and microvascular effects of PAE, and placental PLGF overexpression rescued some of ethanol’s effects on cortical vasculature (Luna et al., 2016; Lecuyer et al., 2017). Together, these data suggest that ethanol repression of placental PLGF expression can contribute to the downstream effects of ethanol on angiogenesis and angiogenesis-related proteins, in both placenta as well as fetal cerebral cortex.

However, the effects observed by Jegou et al. (2012) have been investigated primarily using a model of high PAE that produces peak maternal serum ethanol concentrations of approximately 200 mg/dL 1 h after an intraperitoneal injection. In the present study, we investigated whether voluntary consumption of more moderate levels of PAE would produce similar changes in placental and fetal cortical protein expression. We employed our rat model of voluntary drinking during pregnancy which produces peak maternal serum ethanol concentrations of only 60 mg/dL (Davies et al., 2019) yet produces offspring with long-lasting deficits in hippocampal synaptic plasticity (Varaschin et al., 2010) and learning (Savage et al., 2010). We employed a proteomic approach to screen for ethanol-induced alterations in placental protein expression and then used western blotting to confirm alterations in two of these proteins in placenta. Subsequently, we examined whether the same two proteins were altered in fetal cerebral cortex also, and whether these alterations would be associated with downstream measures sensitive to PAE-induced changes in the expression of these proteins.

## Materials and Methods

### Materials

Unless indicated otherwise in parenthetical text, all reagents were acquired from Millipore Sigma (St. Louis, MO, United States) or from VWR International (Fontenay-sous-Bois, France).

### Voluntary Drinking Paradigm

Four-month-old Long-Evans rat breeders (Harlan Industries, Indianapolis, IN, United States) were single-housed in plastic cages at 22°C and maintained on a “reverse” 12-h dark/12-h light schedule (lights on from 2100 to 0900 h) with Harlan Teklad rat chow and water *ad libitum*. After 1 week of acclimation to the animal facility, all female rats were provided 0.066% saccharin in tap water for 4 h each day from 1000 to 1400 h. On Days 1 and 2, the saccharin water contained 0% ethanol and on Days 3 and 4, the saccharin water contained 2.5% ethanol (v/v). On Day 5 and thereafter, the saccharin water contained 5% ethanol. Daily 4-h ethanol consumption was monitored for 2 weeks and the mean daily ethanol consumption determined for each rat. Females that drank less than one standard deviation below the mean of the entire group were removed from the study. The remainder of the females were assigned to either a saccharin control or 5% ethanol drinking group and matched such that the mean pre-pregnancy ethanol consumption by each group was similar.

Subsequently, females were placed with proven male breeders until pregnant, as evidenced by the presence of a vaginal plug. Female rats did not consume ethanol during the breeding procedure, which typically required 1 to 3 days of housing with male breeders. Beginning on Day 1 of gestation, rat dams were provided saccharin water containing either 0 or 5% ethanol for 4 h a day. The volume of 0% ethanol saccharin water provided to the controls was matched to the mean volume of saccharin water consumed by the 5% ethanol-drinking group. Daily 4-h ethanol consumption was recorded for each dam.

### Maternal Serum Ethanol Levels

A separate set of 12 rat dams were used to determine serum ethanol concentrations. These dams were run through the same voluntary drinking paradigm as described above, except that 45 min after the introduction of the drinking tubes on each of three alternate days during the third week of gestation (gestational days 13, 15, and 17), each rat dam was briefly anesthetized with isoflurane. The 45 min time point was selected for sampling serum ethanol level because the mean peak ethanol consumption occurs over the first 15 to 30 min after the introduction of the drinking tubes each day (Davies et al., 2019). One hundred μL of whole blood was collected from the tail vein and immediately mixed with 0.2 mL of 6.6% perchloric acid, centrifuged at 3,500×*g* at 4°C to collect serum and then frozen and stored at −20°C until assayed. Serum ethanol standards were created by mixing rat whole blood from untreated rats with known amounts of ethanol ranging from 0 to 240 mg ethanol/dL and then mixing 100 μL aliquots of each standard with perchloric acid and storing the standards frozen with the samples. Serum ethanol samples were assayed using a modification of the method of Lundquist (1959).

### Tissue Preparation

The tissue preparative procedures employed in this study were selected to minimize tissue and protein degradation during the approximately 5 to 8 min required to harvest and freeze placental and brain tissues. On gestational day 20, female rat dams were sacrificed by decapitation and a caesarian section rapidly performed, the uterine horns dissected and immediately submerged in ice-cold phosphate-buffered saline (PBS) (0.9% NaCl). All subsequent procedures were performed on ice-chilled dissection plates. Placental:fetal units were dissected and then separated noting the uterine horn position for each unit. Samples selected for a given study excluded the most distal and the most proximal placental:fetal units relative to the uterine horn bifurcation. Fetal sex was noted at the end of the dissections and roughly equal numbers of male and female units were used in each experiment. Placentas were saline-perfused to remove blood and then flash frozen in liquid N_2_. Subsequently, placentas used in the proteomic studies were pulverized with a mortar and pestle under liquid N_2_ and then lyophilized. For the western blotting studies, the fetal cerebral cortex was dissected from the head and flash frozen in liquid N_2_. For the histological studies, whole fetal heads were removed and frozen in isopentane chilled in a dry-ice methanol bath. For the proteomic and microvascular density studies, placental and fetal head tissues were shipped to the University of Rouen on dry ice by next-day freight. All tissues were stored in airtight containers at −80°C until assay.

### Proteomic Analyses

Protein samples were resuspended in IsoElectricFocusing (IEF) buffer (5 M urea, 2 M thiourea, 33 mM CHAPS, 2 mM tributylphosphine, and 10 mM DTT). After centrifugation for 20 min at 10,000 × *g*, the amount of proteins in the supernatant was determined using the Bio-Rad protein assay (Bio-Rad, Hercules, CA, United States). Protein patterns were analyzed by two-dimensional polyacrylamide gel electrophoresis (2D-PAGE). One hundred mg of protein sample was added to Destreak^TM^ Rehydration Solution (GE Healthcare, Sigma-Aldrich Chimie SARL, Saint Quentin Fallavier, France) and to 2% (v/v) carrier ampholytes (pH 3–11 NL, GE Healthcare) in a final volume of 450 μL. The first dimension gel separation was carried out with Immobiline Dry Strips NL (24 cm, pH 3–11 NL, GE Healthcare). After passive rehydration of the strip overnight, IEF was performed with the Multiphor II electrophoresis system (Amersham-BioSciences, GE Healthcare, Velizy-Villacoublay, France) up to a voltage load of 180,000 Vh. After two equilibration steps using 123 mM DTT and 135 mM iodoacetamide, respectively, the second dimension was obtained by SDS-PAGE using a 12.5% (w/v) polyacrylamide separating gel in a Protean II xi cell (Bio-Rad). Proteins were visualized by silver nitrate staining (Lelong et al., 2009).

Gels were scanned using Proxpress (Perkin Elmer Villebon sur Yvette, France), followed by image comparison with “Progenesis SameSpots” software (Version 4.0, Non-Linear Dynamics, Newcastle, United Kingdom). The gels (four gels per condition) were then aligned and matched together. The corresponding spots in different gels were assigned with the same ID number (spot alignment positions). Three statistical filters (ANOVA, power and *q*-value) are included in the software for the differential analysis of the gels. The ANOVA test return a *p*-value that takes into account the mean difference and the variance. The power reflects the confidence in the experimental data’s ability to find the differences that actually exist. Q-value is the name given to the adjusted *p*-value found using an optimized FDR (False discovery rate) approach. Thus, only spots with ANOVA *p* < 0.05, power > 0.8, *q*-values <0.05 and with a fold change of 1.5 between the 2 conditions are selected.

Following the comparative analyses of the gels, all the protein spots differentially expressed between the two conditions were cut (ProXCISION, PerkinElmer, Villebon sur Yvette, France) and digested. Trypsin digestion was performed with an automatic digester (MultiPROBE II, PerkinElmer, Villebon sur Yvette, France) by using a protocol previously described (Coquet et al., 2006). After lyophilization, the peptide extracts were resuspended in 10 μL of 0.2% formic acid and 3% CH_3_CN for nanoLC-MS/MS analyses. Five μL of peptides were injected onto a lab-on-a-chip (Agilent Technologies, Santa Clara, CA, United States). The peptides were further fragmented using an on-line XCT mass spectrometer (Agilent) and were extracted using the Data Analysis program (Version 3.4, Bruker Daltonic, Billerica, MA, United States). For protein identification, the fragmentation data were compared against the *rattus* NCBIprot 20171205 database (77 467 sequences) using the Mascot Daemon (Version 2.6.0, Matrix Science, London, United Kingdom) search engine. All searches were performed with no fixed modification and with variable modifications for carbamidomethylation of cysteines and for oxidation of methionines and with a maximum of one missed cleavage. Tandem mass spectrometry spectra were searched with a mass tolerance of 1.6 Da for precursor ions and 0.8 Da for fragment ions. Peptides matching an individual ion score >45 were considered. However, the peptides with a lower score because of their short length but identified mainly from the ions *y* and *b* were also considered. Functional analysis of the identified proteins was done using the Gene Ontology (GO) database^1^. A GO data research was performed for each protein specifying the gene name and the species. The Rat Genome Database (RGD) link and the GO annotations gave access to the protein characteristics including biological processes, cellular components, and molecular functions.

### Western Blot Analyses

Samples of placental or fetal cerebral cortex were homogenized in 40 mM Tris pH 8.5 with 1% SDS and protease inhibitor cocktail (Roche, Basel, Switzerland). Samples were then sonicated at 30 Hz and centrifuged at 14,100 × *g* for 5 min at 4°C. The supernatant was removed and flash frozen in liquid nitrogen until used in protein quantification. Proteins were quantified using the Pierce^TM^ BCA Protein Assay Kit (Thermo Scientific, Rockford, IL, United States) according to manufacturers’ instructions. Twenty-five μg of total proteins were loaded per lane on a 12% SDS polyacrylamide gel and run at 100 V. The proteins were transferred onto a PVDF membrane for 1 h at 100 V. The membranes were blocked using I-block in TBS at room temperature for an hour and then incubated with the primary antibodies against Annexin-A4 (ANX-A4) (1:10,000; sc-1930, Santa Cruz, Dallas, TX, United States), cerebral cavernous malformation protein 3 (CCM-3) (1:500; sc-365586, Santa Cruz), and to β-actin (1:2000; #4970, Cell Signaling, Danvers, MA, United States) overnight at 4°C in I-block containing 0.1% Tween-20. Membranes were then washed 4 × 5 min in TBS-T, incubated in corresponding secondary antibodies (1:15,000, LI-COR Biosciences, Lincoln, ME, United States) in I-block containing TBS plus 0.1% Tween-20 and 0.01% SDS for 45 min at room temperature. Membranes were washed 4 × 5 min in TBS-T and an additional 5 min in TBS. Membranes were stored in TBS at 4°C until scanned. Membranes were scanned and analyzed using the Li-Cor Odyssey Infrared Imaging System (Lincoln, NE, United States) in the 700 and 800 nm channels. The optical densities of the proteins of interest were normalized to β-actin.

### *In vitro* [^125^I]-VEGF Autoradiography

The methods employed to visualize labeled VEGF binding to VEGF receptors were adapted from Jakeman et al. (1992) and Cooper et al. (1999). Twelve-mm-thick microtome cryostat sections were collected in the sagittal plane corresponding to Sagittal Plate 6 in the E20 rat of the Altman & Bayer stereotaxic atlas (Altman, 1995). The sections were thaw-mounted onto pre-cleaned Superfrost-Plus^®^ microscope slides and stored at −80°C in airtight containers until assay. Tissue sections were preincubated for 10 min in incubation buffer (10 mM HEPES, 100 mM NaCl, 5 mM MgCl_2_, 5 mM KCl, 1.0 mM EGTA, 100 mg/mL bacitracin, and 0.1% bovine serum albumin; pH 7.4) at 25°C and then incubated with 22.5 fM [^125^I]-rhVEGF(165) (Perkin Elmer, specific activity = 3,832 Ci/mmole) for 90 min at 25°C in the absence (total binding) or presence of 1.5 nM unlabeled VEGF (non-specific binding). After incubation, sections were rinsed twice for 5 min each in ice-cold incubation buffer, dipped in ice-cold distilled water, dried and placed in a vacuum desiccator overnight. Sections were then exposed to Kodak Biomax MR film for 1 week and then the film developed in Kodak D-19 (1:1) and fixed. Microdensitometric measurement of [^125^I]-VEGF binding was performed using Media Cybernetics Image Pro Plus^®^ (Silver Spring, MD, United States) on an Olympus BH-2 microscope. An optical density standard curve, expressed in picoCuries/10^5^ μm^2^, was established based on autoradiograms of ^14^carbon standards. Total and non-specific [^125^I]-VEGF binding were measured in three zonal regions of the fetal cortex at 3.125× total magnification. For each region of interest, specific [^125^I]-VEGF binding was defined as the difference between total VEGF binding in quadruplicate sections and non-specific VEGF binding in duplicate sections.

### Cerebrocortical Microvascular Analysis

Forty-μm-thick cryomicrotome sections of frozen fetal brain were collected in transverse slices similar to the sagittal plane described above. Histological slices were incubated in 0.1 M PBS containing 4% paraformaldehyde for 20 min at 24°C, rinsed three times in PBS and incubated overnight at 4°C with isolectin B4-FITC (1 mg/ml in 0.1 M PBS containing 0.3% Triton X-100). After a 5-min wash with PBS, slices were examined under a DMI 6000 fluorescence microscope (Leica, Rueil-Malmaison, France) equipped with a CCD camera (Retiga Exit, Qimaging, Roper Scientific, Evry, France) to visualize the microvascular network. Microvascular density was measured by a ratiometric approach using the Metamorph software (Roper Scientific, Lisses, France). Images were acquired at standardized conditions of magnification and illumination and saved in TIFF file format using the computer-assisted image analysis station from Roper Scientific. For analysis of the vessel density, a threshold was set in order to distinguish the isolectin B4-FITC-positive structures from the background and the corresponding regions of interest (ROI) were selected by segmentation. For each ROI, the ratio of “vascular area/cortical area” was calculated by the Metamorph Software. For each ROI, eight to twelve measures were collected from two sections to obtain a single average value for each ROI from each subject. For analysis of the vascular organization, the angular orientation of microvessels was quantified using the Metamorph Software (Lecuyer et al., 2017). The quantification was performed in the fronto-parietal cortex and a frame of lines was defined perpendicular to the cortical border for each section. For microvessels parallel to these lines, the Metamorph Software arbitrarily attributed the angular value 0° and the maximal angular value was 90°. For better comprehension, angular values were distributed into four groups: 0°–25°, 25°–50°, 50°–75°, and 75°–90°.

### Study Design Issues and Statistical Analyses

For each type of study, no more than one offspring was used from a litter to avoid the potential confound of “litter effects.” All experiments and analyses were performed by investigators blinded to the prenatal treatment condition. With the exception of two control samples lost in the placental Western blot studies and one 5% ethanol sample lost in the microvascular density experiment, all other analyses were conducted in pairs of samples from each prenatal treatment group. There were no exclusion criteria and no data were excluded from the analyses.

**FIGURE 1:**
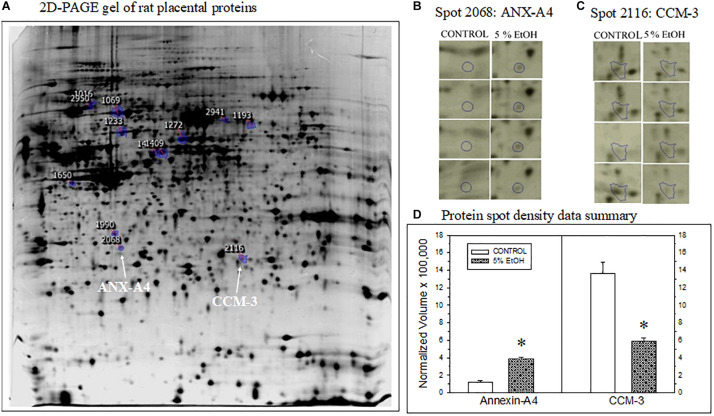
Impact of maternal drinking during pregnancy on placental protein expression. **(A)** Representative 2D-PAGE gel of rat placental proteins prepared from a saccharin control rat. Blue-circles on the gel denote location and spot identification numbers differentially expressed in controls compared to gels prepared from 5% ethanol-consuming dam harvested on gestational day 20. Spot 2068 subsequently identified as annexin-A4 (ANX-A4) and Spot 2116 as cerebral cavernous malformation protein 3 (CCM-3) are denoted by the white arrows. **(B,C)** Protein spot location images for ANX-A4 **(B)** and CCM-3 **(C)** on each gel from the four controls and four ethanol-exposed placental samples. **(D)** Summary of the protein spot density data, expressed as normalized volumes, for the protein spots illustrated in panels **(B,C)**. Data bars represent the mean + S.E.M. protein density from four pairs of control and 5% ethanol placentae. Asterisks denote data significantly different from saccharin control: Anx-A4, *t* = 12.3, *p* < 0.001; CCM-3, *t* = 5.68, *p* = 0.001.

All statistical procedures and graphical illustrations were performed using SigmaPlot^®^ 11 (Systat Software Inc., San Jose, CA, United States). The effects of PAE on maternal weight gain during pregnancy, litter size, placental wet weight, and fetal pup weight (Table 1) were conducted using a Mann–Whitney Rank Sum test. The effects of PAE on placental protein spot density (Figure 1D), Western blot densities (Figures 3C, 4C), VEGF binding (Figure 5C), microvascular density (Figure 6D), and microvascular organization (Figure 6E) were all compared using a Student’s two-tailed *t*-test. Sex-related differences were examined as part of the Western blotting studies given that the sample sizes in this experiment were sufficient to provide adequate power to examine sex-related differences. No sex-related differences were observed for either protein in either tissue type. Thus, the western blot data was collapsed for sex as was the analysis of all other data types for which the total sample size was too small to test for sex-related differences. All data is expressed as the mean ± standard error of the mean (S.E.M.) and a value of *p* < 0.05 was deemed as statistically significant.

**TABLE 1:** Effects of daily 4-h consumption of 5% ethanol on rat dams and their off spring.

	Saccharin control	5% Ethanol
Daily 4-h ethanol consumption	NA	2.04 ± 0.04^a^ (40)
Maternal serum ethanol concentration	NA	60.8 ± 5.8^b^ (35)
Maternal weight gain during pregnancy	105 ± 2 (38)	105 ± 2^c^ (40)
Litter size	12.5 ± 0.13 (38)	12.4 ± 0.30^d^ (40)
Placental wet weight	0.53 ± 0.02 (38)	0.51 ± 0.02^e^ (40)
Fetal pup weight	6.17 ± 0.13 (38)	6.13 ± 0.11^f^ (40)

*^*a*^Mean ± S.E.M. grams ethanol consumed/kg body weight/day. ^*b*^Mean ± S.E.M. mg ethanol/dL serum, 45 min into the drinking period. ^*c*^Mean ± S.E.M. grams increase in body weight from GD 1 through of GD 20. ^*d*^Mean ± S.E.M. number of live fetal pups/litter. ^*e*^Mean ± S.E.M. grams placental wet weight on gestational day 20. ^*f*^Mean ± S.E.M. grams fetal pup weight on gestational day 20. NA, not applicable. (*n*), Group sample size.*

## Results

### Voluntary Drinking Paradigm

Results from voluntary drinking paradigm are summarized in Table 1. Rat dams in the 5% ethanol group consumed 2.04 ± 0.04 g/kg/day during the daily 4 h period. This level of consumption produced a mean maternal serum ethanol concentration of 60.8 ± 5.8 mg/dL, 45 min after the introduction of the drinking tubes during the third week of gestation (Davies et al., 2019). This level of ethanol consumption produced no significant effects on maternal weight gain during pregnancy or on fetal litter size, fetal pup wet or placental wet weight on gestational day 20 (Table 1).

### Proteomic Analysis of Placental Protein Expression

Two-dimensional PAGE of placental tissue detected a total of 1,117 distinct spots in all four pairs of placental tissue samples (Figure 1A). Densitometric analysis of the four pairs of 2D gels indicated that 185 of the protein spots exhibited significant differences between prenatal treatment groups. The fold change difference was greater than 1.5 for 19 of these proteins. The spots for these 19 proteins were subsequently excised and processed for XCT mass spectrometry. Two of the 19 protein spots altered by ethanol exposure were identified as ANX-A4 and CCM-3. The location of these two proteins on the 2D gel is shown in Figure 1A. A higher magnification of these two protein spots on all eight gels is illustrated for ANX-A4 and CCM-3 in Figures 1B,C, respectively. ANX-A4 spot densities were clearly darker in all four ethanol-exposed placental samples compared to control (Figure 1B). In contrast, ethanol exposure reduced CCM-3 spot density in three of the four pairs of samples (Figure 1C). A summary of the spot density data is illustrated in Figure 1D. Ethanol exposure significantly elevated placental ANX-A4 expression and significantly reduced placental CCM-3 expression.

**FIGURE 2:**
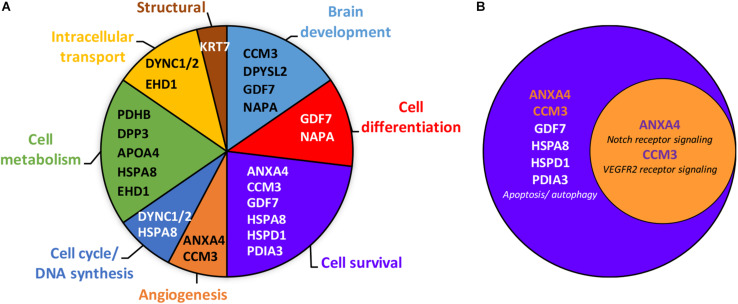
Gene ontology analysis of the 14 placental proteins significantly altered by prenatal ethanol exposure. **(A)** Clustering of the placental proteins by biological processes revealed eight clusters, with the proteins belonging primarily to the cell survival, cell metabolism and brain development clusters. Gene names of each protein are mentioned within clusters. Full name proteins and GO term numbers related to each biological process are provided in the Supplementary Table S1. **(B)** Venn diagram showing that annexin-A4 and CCM-3 belong to both the cell survival and angiogenesis clusters.

In addition to ANX-A4 and CCM-3, mass spectrometry identified 12 other proteins from the 19 protein spots altered by PAE. Protein identification results from the Mascot analysis are available in a Supplementary Data file is: Mascot Search Results.pdf^2^. Table 2 summarizes the fold-change data for all 14 identified proteins. The expression of six of these proteins was up-regulated by ethanol exposure whereas eight proteins were repressed (Table 2). The Mascot score values, which reflect the matching between the experimental data and the identified database protein sequence, ranged from 32 to 355 (for indication, a 95% confidence level is around a Mascot score of 90). GO analysis targeting biological processes regulated by these 14 proteins indicated that they are mainly involved in cell survival, the regulation of cell metabolism, and brain development (Figure 2A). Interestingly, ANX-A4 and CCM-3 are both associated with angiogenesis and the cell survival clusters (Figure 2B). Supplementary Table S1 illustrates the different biological processes associated with one or more of the placental proteins whose expression was altered by prenatal ethanol exposure.

**TABLE 2:** Effects of maternal drinking during pregnancy on placental protein expression.

Spot ID #	Access No. (NCBI)	Proteins^a^	Fold change (EtOH/Control)	Mascot Score^b^	TNPM/NPM/PC (%)^c^	pI/MW (kDa) (theoretical)
2068	P55260.3	Annexin-A4	3.20	167	4/3/16	5.31/36
1990	NP_542152.1	NEM-sensitive factor attachment protein alpha*	3.00	58	1/1/4	5.30/33
	NP_001007621	Pyruvate dehydrogenase, beta*	3.00	39	1/1/4	6.20/39
2950	NP_446200.1	Dipeptidyl peptidase III	2.60	61	5/1/10	5.12/83
1016	Q9JII4.1	Cytoplasmic dynein intermediate chain 2*	2.60	32	1/0/3	5.11/71
1650	P02651.2	Apolipoprotein A-IV	1.90	103	9/2/43	5.12/44
1409	XP_003754401.1	Keratin 7 CRA_a	0.67	355	15/10/26	5.67/51
1069	S31716	Heat shock protein 8 (Hsp 72)	0.67	125	5/3/8	5.43/71
1233	CAA38564.1	Heat shock protein (Hsp 60)	0.63	91	3/2/5	5.91/61
1272	P11598.2	Protein disulfide isomerase 3	0.56	92	6/2/14	5.88/57
1193	NP_034249.1	EH-domain containing protein 1	0.56	76	2/2/5	6.35/61
1412	XP_006239940.1	Growth differentiation factor 7, isoform 1*	0.50	34	1/0/7	9.15/48
2116	NP_001009542.1	Cerebral cavernous malformation protein 3	0.43	103	4/3/27	6.78/24
2941	NP_034085.2	Dihydropyrimidinase-like protein 2	0.25	59	1/1/1	5.95/62

^*a*^Differentially expressed proteins between EtOH and control conditions after analysis with Progenesis SameSpots software. ^*b*^From all identified peptides. ^*c*^Total number of peptide matches/number of peptide matches with peptides exhibiting significant individual ion scores/protein coverage. *Identification from one peptide with a significant or non-significant score but validated since mainly identified from y and b ions.

**FIGURE 3:**
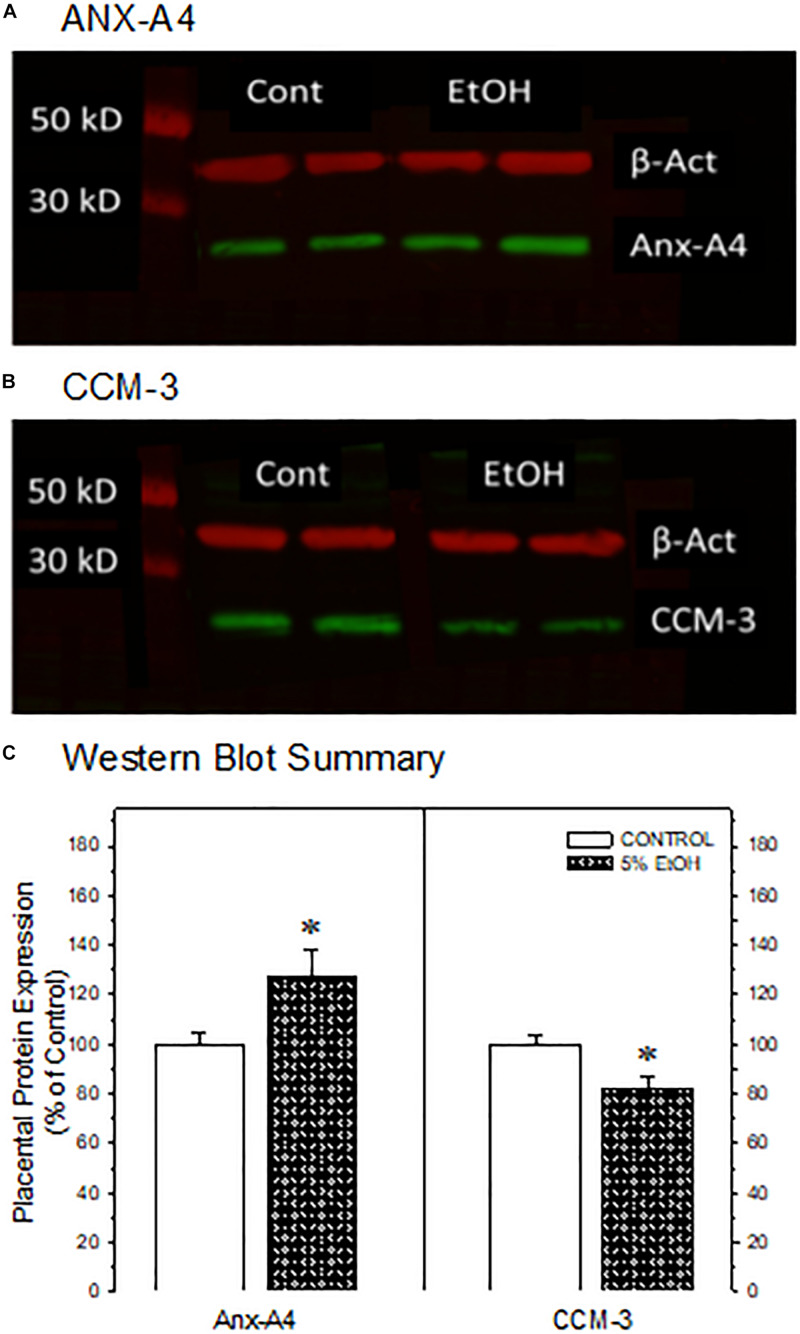
Western blot confirmation of the impact of maternal drinking during pregnancy on the expression of annexin-A4 and CCM-3 in placental tissue collected on gestational day 20. Representative Western blots of ANX-A4 **(A)** and CCM-3 **(B)** from two separate samples of placenta from each prenatal treatment group. **(C)** Summary of Western blot data for placental ANX-A4 and CCM-3. Data bars represent the mean ± S.E.M. protein expressed as percent of the saccharin control group for 14 control and 16 5% ethanol placental samples. Asterisks denote data significantly different from saccharin control: Anx-A4, *t* = 2.26, *p* = 0.032; CCM-3, *t* = 3.00, *p* = 0.006.

**FIGURE 4:**
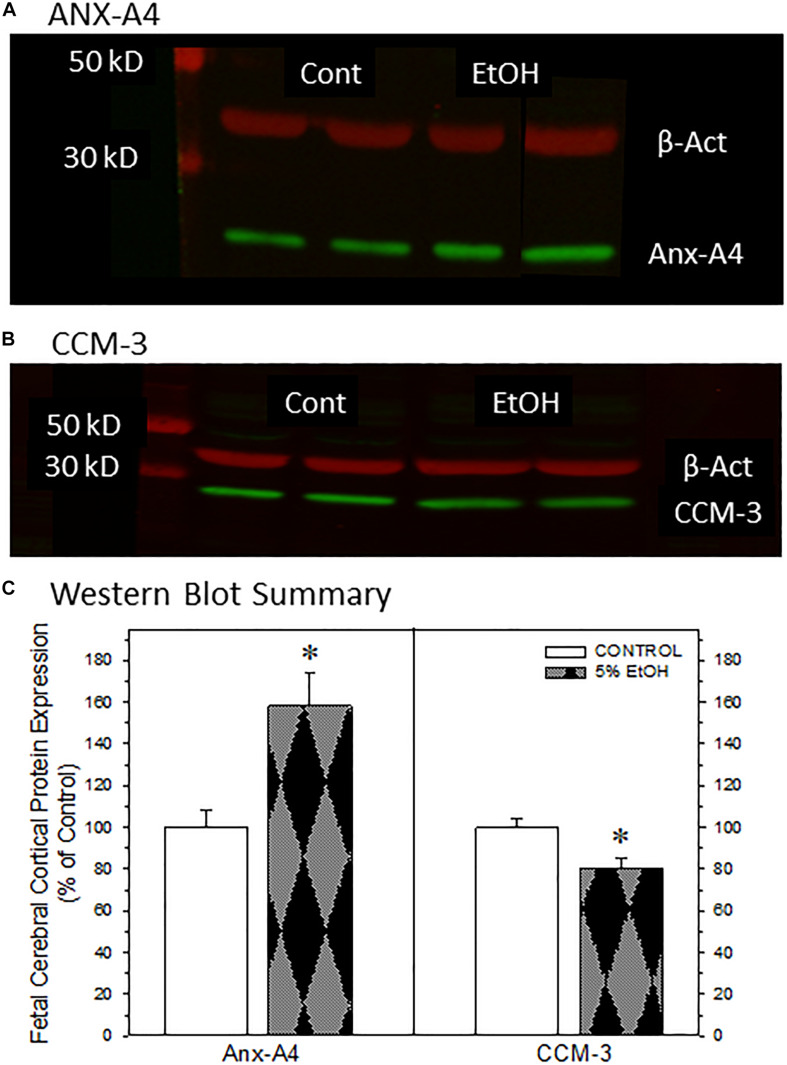
Impact of maternal drinking during pregnancy on the expression of annexin-A4 and CCM-3 in fetal cortical tissue collected on gestational day 20. Representative Western blots of ANX-A4 **(A)** and CCM-3 **(B)** from two separate samples of fetal cerebral cortex from each prenatal treatment group. **(C)** Summary of Western blot data for fetal cortical ANX-A4 and CCM-3. Data bars represent the mean ± S.E.M. protein expressed as percent of the saccharin control group from eleven pairs of control and 5% ethanol fetal cortical samples. Asterisks denote data significantly different from saccharin control: Anx-A4, *t* = 3.56, *p* = 0.003; CCM-3, *t* = 3.00, *p* = 0.007.

**FIGURE 5:**
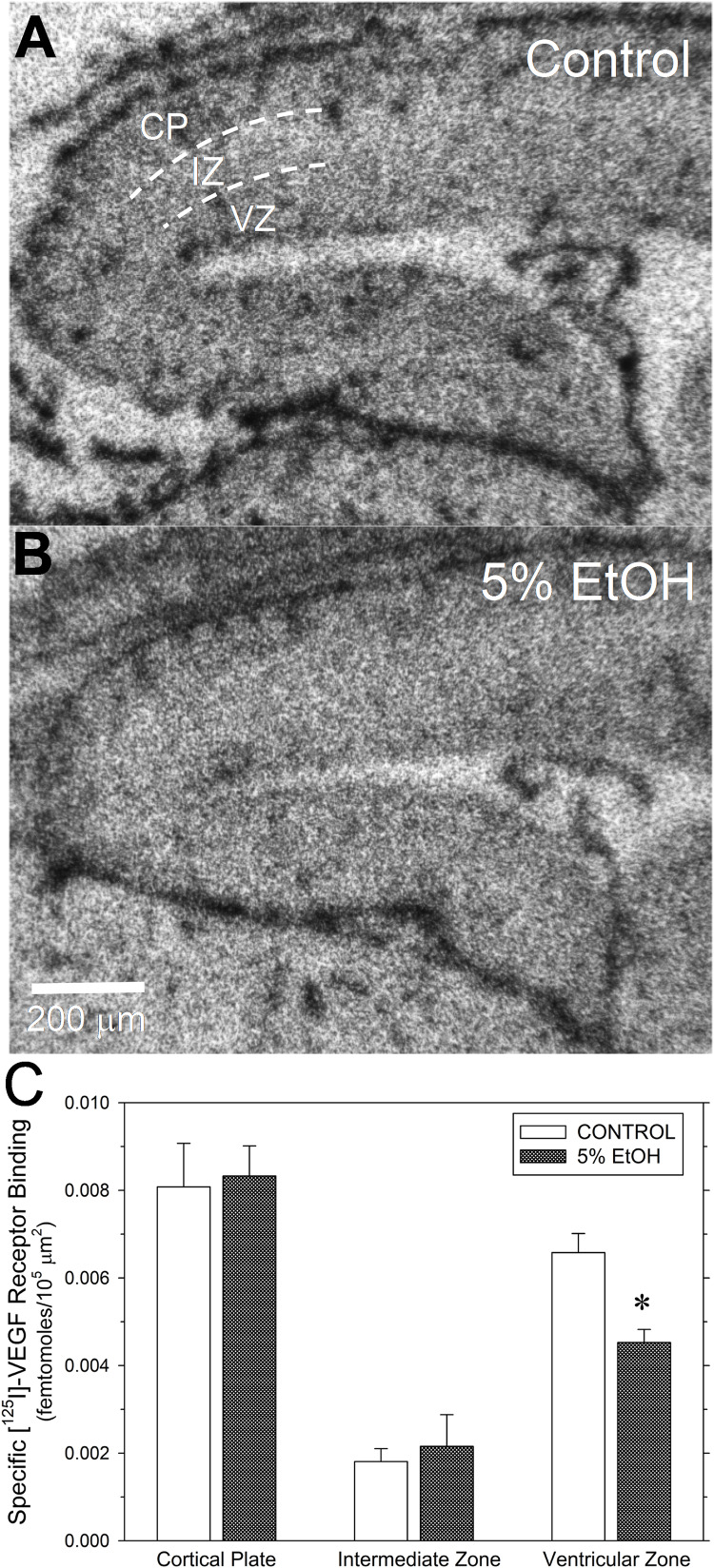
Impact of maternal drinking during pregnancy on [^125^I]-VEGF receptor binding density in fetal cerebral cortex on gestational day 20. **(A,B)** Autoradiograms of total [^125^I]-VEGF binding in sagital sections of fetal cortex from a saccharin control **(A)** and a 5% ethanol-exposed fetus **(B)**. Binding site densities were measured between the dotted line demarcations in three cortical zones, denoted in **(A)**, as the cortical plate (CP), the intermediate zone (IZ), and the ventricular zone (VZ). **(C)** Summary of the specific [^125^I]-VEGF receptor binding data. Data bars represent the mean ± S.E.M. specific [^125^I]-VEGF binding, expressed as femtomoles of binding per 10^5^ mm^2^ from seven pairs of saccharin control and ethanol-exposed fetuses. Asterisks denote data significantly reduced compared to saccharin control: *t* = 3.89, *p* = 0.002.

**FIGURE 6:**
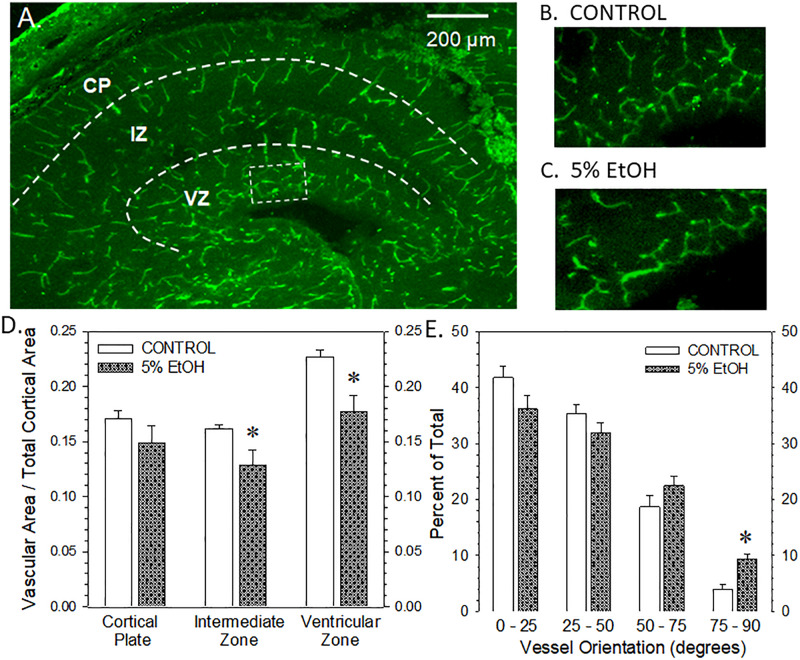
Impact of maternal drinking during pregnancy on fetal cortical microvascular density on gestational day 20. **(A)** Visualization of the microvasculature elements across three zones within in fetal cortex, namely, the cortical plate (CP), the intermediate zone (IZ), and the ventricular zone (VZ). **(B,C)** The rectangular box in panel **(A)** denotes the region magnified in the ventricular zone of a saccharin control **(B)** and an ethanol-exposed fetus **(C)**. **(D)** Summary of the vascular density measurements made in each of the three zones of fetal cerebral cortex. Data bars represent the mean ± S.E.M. of the microvascular area relative to the total area measured in each cortical zone from nine control and eight ethanol-exposed fetuses. Asterisks denote data significantly reduced compared to saccharin control: Intermediate zone, *t* = 2.328, *p* = 0.0295; ventricular zone, *t* = 3.147, *p* = 0.0047. **(E)** Summary of the vessel orientation measurements made in the ventricular zone of the fetal cerebral cortex. Data bars represent the mean ± S.E.M. of the percentage of microvessels having an angular orientation between 0° and 25° (radial), 25° and 50°, 50° and 75°, and 75° and 90°. Asterisks denote data significantly decreased compared to saccharin control, *t* = 4.63, *p* = 0.0003.

### Western Blot Confirmation of Placental Protein Alterations

We focused subsequent protein confirmation studies on ANX-A4 and CCM-3. Figure 3 summarizes Western blotting studies in placenta. Representative Western blots of placental ANX-A4 and CCM-3 are illustrated in Figures 3A,B, respectively. Placental expression of the reference protein, β-actin, was not altered by ethanol exposure (data not shown). However, as summarized in Figure 3C, placental ANX-A4 expression was significantly elevated and CCM-3 expression significantly decreased in ethanol-exposed placenta, confirming the results of the proteomic analyses (Figure 1D).

### Western Blot Analysis of Fetal Cortical Proteins

We next examined whether ANX-A4 and CCM-3 would be altered in fetal cerebral cortex of PAE offspring in a manner similar to the alterations observed in placenta. Figure 4 summarizes the Western blotting studies in fetal cerebral cortex. Representative Western blots for ANX-A4 and CCM-3 in fetal cortex are illustrated in Figures 4A,B, respectively. Fetal cerebral cortical expression of β-actin was not altered by ethanol exposure (data not shown). In contrast, fetal cortical ANX-A4 expression was significantly elevated and CCM-3 expression significantly reduced in PAE offspring compared to control (Figure 4C). These alterations were in the same directions as the changes observed for these proteins in placenta (Figure 3C).

### Specific [^125^I]-VEGF Binding

Given that CCM-3 impacts angiogenesis and regulates VEGFR2 endocytosis and expression in vascular endothelium (Fisher and Boggon, 2014), we investigated the effect of ethanol exposure on [^125^I]-VEGF binding to VEGF receptors in fetal cerebral cortex. Under the assay conditions and tissue type employed here, specific [^125^I]-VEGF binding exceeded 90% of total [^125^I]-VEGF binding. Figure 5 illustrates the effects of ethanol exposure on [^125^I]-VEGF binding in GD20-old fetal cerebral cortex. Total [^125^I]-VEGF binding was highest in the cortical plate, with intermediate levels of binding in the ventricular zone and relatively low binding in the intermediate zone (Figure 5A). By contrast, VEGF binding site density appeared to be lower in the ventricular zone region from an ethanol-exposed fetal brain (Figure 5B), such that the boundary between the intermediate zone and the ventricular zone was less distinct than in control. A summary of the specific [^125^I]-VEGF binding provided substantiates this observation. Specific [^125^I]-VEGF binding was significantly reduced in the ventricular zone of PAE fetal cortex with no significant changes in the other two regions (Figure 5C).

### Fetal Cerebral Cortical Microvascular Density

The visualization of fetal cortical microvasculature and the impact of moderate drinking during pregnancy of microvascular density are presented in Figure 6. Figure 6A illustrates the distribution of isolectin B4-FITC-labeled cerebrocortical microvessels in a transverse section taken from a saccharin control fetus on gestational day 20. In the cortical plate and the intermediate zone, microvessels present a radial orientation while, in the ventricular zone, vessels are preferentially organized in a meshed network. Figures 6B,C are higher magnification images of the ventricular zone from a saccharin control and ethanol-exposed fetuses, respectively. A comparison of Figures 6B,C illustrates a reduction in vascular density in the meshed network within the ventricular zone. Figure 6D summarizes the vascular density data from all three cortical regions. Ethanol exposure significantly reduced vascular density in the intermediate and ventricular zones, though the most striking effect was observed in the ventricular zone. The alcohol-induced alteration in vessel density in the ventricular zone was also associated with a significant impairment of their angular orientation (Figure 6E). In particular, the percentage of vessels with a radial orientation (angle values between 0° and 25°) tends to decrease in favor of a significant increase of perpendicular (75°–90°) vessels indicating that alcohol impaired the positioning of microvessels in this layer.

## Discussion

The salient observations from this study are that moderate drinking during pregnancy alters the expression of more than a dozen placental proteins (Table 2) and that the changes in at least two placental proteins (Figure 3C) are mirrored in fetal cerebral cortex (Figure 4C). Further, the reductions in fetal cortical CCM-3 expression are associated with the effects ethanol exposure on VEGF receptor expression and microvascular density and organization in fetal cerebral cortex (Figures 5C, 6D,E). It is noteworthy that these three proteins were not identified in previous high-throughput screening surveys of proteins altered by PAE (Datta et al., 2008; Ramadoss and Magness, 2012; Davis-Anderson et al., 2017). These differential outcomes may be due to differences in animal models of PAE, particularly in dosing or patterns of alcohol exposure. In addition, our prior work examining the impact of moderate PAE on placental gene expression (Rosenberg et al., 2010) did not identify parallel changes in either ANX-A4 or CCM-3 mRNA expression. More recently, we have observed that the placental and fetal cortical expression of miRNAs known to regulate ANX-A4 or CCM-3 mRNA expression are not altered by PAE (Davies et al., 2019) in a manner that would be consistent with the observed changes in placental ANX-A4 or CCM-3 protein expression in the current study. These differences underscore the impression that prenatal ethanol-induced alterations in protein expression may provide greater fidelity for linkage with functional consequences in PAE than other biochemical species under consideration as biomarker candidates.

Annexin-A4 is a member of a family of calcium-dependent membrane phospholipid binding proteins expressed primarily in epithelial cells. Placental ANX-A4 increases near term, where it is thought to have anticoagulant properties (Masuda et al., 2004). ANX-A4’s expression patterns also change during neurodevelopment (Woolgar et al., 1990). However, ANX-A4 also appears to be involved in mediating calcium-induced neurotoxicity in a variety of neuropathological states. For example, significant elevations of ANX-A4 occur in a variety of cell types including vascular endothelium, reactive astrocytes, oligodendrocytes, ependymocytes, choroid plexus, and meningothelium in postmortem brain samples from Alzheimer’s patients (Eberhard et al., 1994). ANX-A4 expression was also elevated in occipital cortex and hippocampal formation and autoantibodies to ANX-A4 were detected in postmortem brain samples of alcoholic patients compared with controls (Sohma et al., 2001). Ethanol exposure dose-dependently elevates ANX-A4 expression in rat C6 glioma and human adenocarcinoma A549 cell lines and overexpression of ANX-A4 increased ethanol-induced cytotoxic damage (Ohkawa et al., 2002; Sohma et al., 2002). Taken together, these studies indicate that ethanol mediates at least some neurocytotoxic damage *via* increased ANX-A4 expression. In contrast to the studies cited above, where elevations in ANX-A4 occurred after either long-term or moderately high levels of ethanol exposure, the elevation in ANX-A4 in placenta and cerebral cortex reported here (Figures 3C, 4C) occurred after relatively moderate levels of ethanol exposure, suggesting that ANX-A4 may be a particularly sensitive indicator of alcohol-induced damage in a variety of tissue types.

Cerebral cavernous malformation protein 3, also known as PDCD10, has been linked to the activation of a number of kinases (see review by Fisher and Boggon, 2014) including triggering downstream DLL4-Notch signaling pathways (You et al., 2013). In the presence of VEGF, CCM-3 is recruited to cell membranes where it protects VEGFR2 receptors from endocytosis (He et al., 2010). Further, endothelial-specific deletion of CCM-3 produces marked deficits in embryonic angiogenesis leading to early death (He et al., 2010). Deficits in CCM-3 result in aberrations in laminar positioning of late-born neurons (Louvi et al., 2014). In developing cerebral cortex, CCM-3 is also required for the migration of pyramidal cells and radial glia through the subventricular zone, but it does not affect migration from the cortical plate. This differential effect in the ventricular zone, in contrast to the cortical plate region, is consistent with the higher expression of CCM3 mRNA in the ventricular zone compared to the cortical plate in GD19 cerebral cortex (Petit et al., 2006). Given that CCM-3 is a critical factor in the stabilization and activation of VEGFR2 receptors on endothelial cells, an ethanol-induced reduction in CCM-3 expression, primarily in the ventricular zone, is one possible factor contributing to the more striking effect of effect of ethanol on [^125^I]-VEGF receptor binding (Figure 5C) and microvascular density (Figure 6D) in the ventricular zone of fetal cerebral cortex. In addition to its impact on angiogenesis and vascular remodeling, reduced VEGF receptor signaling impacts neurite outgrowth (Jin et al., 2006) and axonal elongation (Bellon et al., 2010). Thus, an ethanol-induced decrease in VEGF receptor signaling likely alters the development of synaptogenesis and the establishment of neural networks in affected regions, as well as the development of microvascular networks in brain.

Analysis of the proteomic data using the *Gene Ontology^©^* database revealed a functional association between ANX-A4, CCM-3, and angiogenesis (Notch and VEGFR2 biological pathways; Supplementary Table S1). These results reinforce the notion that angiogenesis would contribute to the deleterious effects of alcohol on both placenta and brain development. Consistent with this hypothesis, Lecuyer et al. (2017) reported that vascular area in human placenta is drastically reduced by prenatal ethanol exposure while in the fetal cerebral cortex, the radial organization of microvessels is disrupted (Jegou et al., 2012; Lecuyer et al., 2017). Furthermore, the placental and brain vascular defects markedly correlate; the greater the reduction in vascular density in the placenta, the more the radial orientation is altered in the fetal brain. The ethanol-induced reductions in perinatal cerebral cortical microvascular density reported here for a moderate alcohol exposure (Figure 6) are qualitatively similar to these previous reports (Jegou et al., 2012; Lecuyer et al., 2017). At a molecular level, alcohol has been shown to reduce PlGF expression in the placenta of rodents at both mRNA (Rosenberg et al., 2010) and protein (Lecuyer et al., 2017) levels. Moreover, the targeted overexpression of the *pgf* gene in the placenta has been shown to prevent the PAE-induced disorganization of microvessels in the fetal brain (Lecuyer et al., 2017). Taken together, these results support the conclusion that the VEGF/PlGF signalosome is one mechanistic target of alcohol during pregnancy. The recent demonstration in *pgf* knock-out mice that cortical microvessels are markedly disorganized supports this hypothesis.

Disorganization of microvascular elements during development impacts how neuronal populations such as GABAergic interneurons correctly migrate to their intended brain regions (Won et al., 2013). Moreover, the antagonism of VEGF signaling disrupts the positioning of GABAergic interneurons (Li et al., 2013) suggesting that the altered angiogenesis induced by PAE is an important mechanism contributing to the deleterious neurodevelopmental effects of alcohol during pregnancy (Wilhelm and Guizzetti, 2015). These cell migration processes are also impacted by a multifactorial trophic environment which involves neurotrophins (BDNF; Carabalona et al., 2016), metalloproteinases (MMP9; Leger et al., 2019), adhesion molecules (ICAM-1; Kevil et al., 2004), and guidance molecules (Marín et al., 2010). The present data indicating the deleterious effects of PAE on angiogenic factors may reflect an even wider alteration of this trophic environment.

**FIGURE 7:**
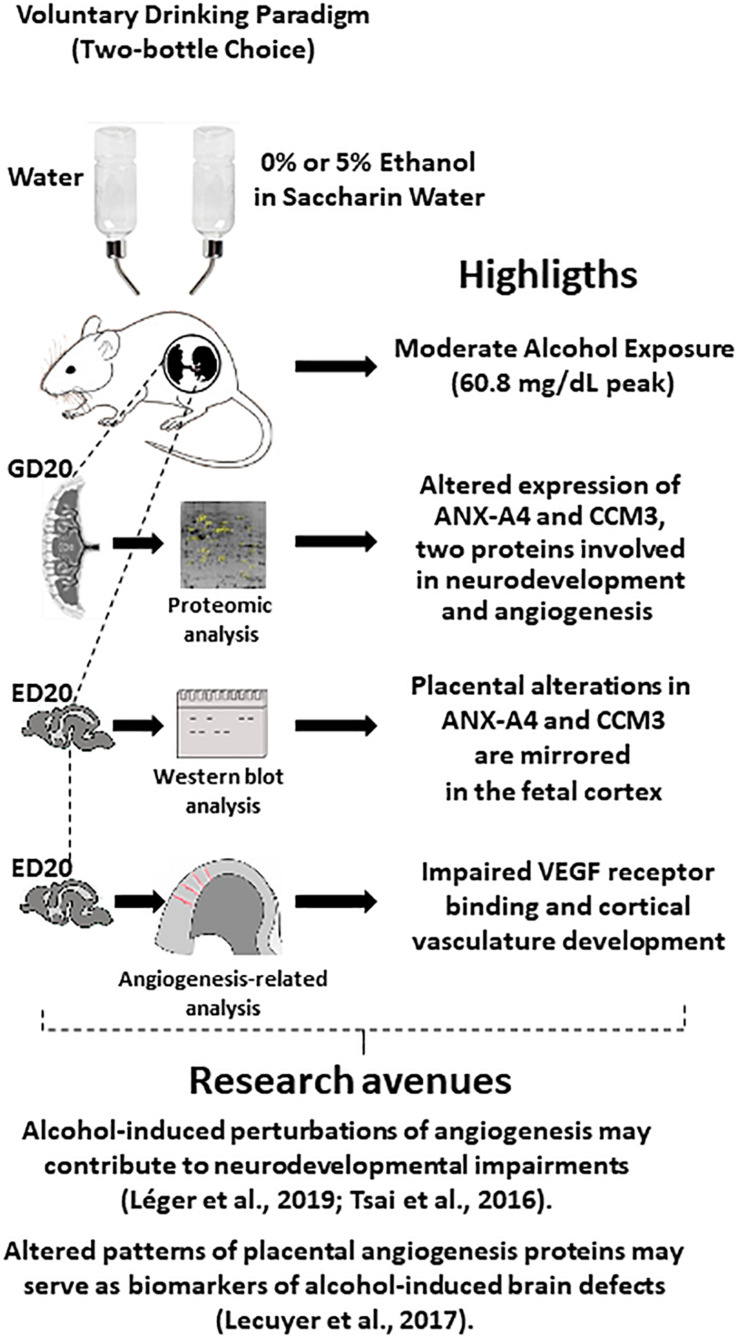
Graphical abstract integrating highlights from the present study with several studies from the literature, which suggest new research avenues. In particular, the fact that cerebral blood vessels constitute guides for nervous cell migration and that *in utero* alcohol exposure alters brain angiogenesis suggest that impaired expression of placental angiogenic proteins by ethanol (present study) may constitute a source of biomarkers of alcohol-induced damage in brain development. ED20, embryonic day 20; GD20, gestational day 20.

Collectively the Gonzalez, Savage and colleagues (Rosenberg et al., 2010; Jegou et al., 2012; Lecuyer et al., 2017) are the first to establish a direct link between prenatal alcohol-induced alterations in placental proteins that are associated with a specific physiological process in a biomarker tissue whose function is critical for fetal neurodevelopment (see Figure 7). As these initial studies were performed in whole placenta, subsequent immunohistochemical studies of ANX-A4 and CCM-3 could provide insights about which cellular entities within the placenta were affected by alcohol exposure. Future studies will examine the impact of moderate drinking during pregnancy on the expression of a larger number of angiogenesis-related proteins. In addition, it will be important to address the extent to which different patterns and levels of ethanol exposure affect the patterns of placental and fetal cerebral cortical protein expression described here. Further, the question of whether the expression of these proteins may be affected by other potentially confounding influences in a clinical study, such as co-exposure to nicotine or other substances of abuse, needs to be addressed. Lastly, recent studies revealed that brain microvessels contribute in the control of nervous cell migration, i.e., GABAergic interneurons (Leger et al., 2019) and oligodendrocytes (Tsai et al., 2016) suggesting that angiogenesis is a prerequisite for normal neurodevelopment. More detailed studies of how altered expression of angiogenesis-related proteins impact vascular development and function in both the placenta and developing brain, and relating these changes to longer-term neurophysiological and behavioral alterations will be required to determine whether altered patterns of placental protein expression have utility for predicting adverse neurodevelopmental outcomes.

## Data Availability Statement

The original contributions presented in the study are included in the article/Supplementary Material, further inquiries can be directed to the corresponding author.

## Ethics Statement

All procedures involving the use of laboratory rats were reviewed and approved by University of New Mexico Health Sciences Center Institutional Animal Care and Use Committee.

## Author Contributions

DS, MR, MP, and NA conducted all of the rat husbandry procedures for the production of experimental tissues for this study. MR prepared tissue samples for the proteomic studies. TJ and LC performed the proteomic studies on placental tissues: 2D PAGE, differential gel analyses, trypsin digestion, mass spectrometric analyses, and protein identifications. MP performed the Western blot studies. NA conducted the [^125^I]-VEGF binding studies. CR, CA, and BG performed the gene ontology analysis and the microvascular density and organization studies. DS and BG developed the experimental design, conducted the statistical analyses of data, and prepared the manuscript for publication.

## Conflict of Interest

The authors declare that the research was conducted in the absence of any commercial or financial relationships that could be construed as a potential conflict of interest.

## Footnotes

1 http://geneontology.org/

2 http://mascot2.crihan.fr/mascot/cgi/master_results_2.pl?file=../data/20
