# Supplementary material for: Ethanol-Induced Alterations in Placental and Fetal Cerebrocortical Annexin-A4 and Cerebral Cavernous Malformation Protein 3 Are Associated With Reductions in Fetal Cortical VEGF Receptor Binding and Microvascular Density
Source: Front Neurosci. 2020 Jun 3;14:519. doi: 10.3389/fnins.2020.00519 (PMC7325964; doi:10.3389/fnins.2020.00519)
Supplement: Supplementary file 1 [file Data_Sheet_1.pdf]

MATRIX SCIENCE MASCOT Search Results

User : pbm
E-mail :
Search title : Submitted from MRosenberg Data2009 by Mascot Daemon on SCT-14-65A4
MS data file : G:\PRESTATIONS\Labos académiques\Internationaux\Etats Unis\Martina Rosenberg\Data pour publi M Rosenberg\Identifications Mascot\MR__2068.mgf
Database : NCBIprot 20171205 (139,213,787 sequences; 51,013,024,959 residues)
Taxonomy : Rattus (77,467 sequences)
Timestamp : 25 Jan 2019 at 10:09:01 GMT

Not what you expected? Try **the peptide summary**.

Search parameters

Type of search : MS/MS Ion Search
Enzyme : Trypsin
Variable modifications : **Carbamidomethyl (C)**, **Oxidation (M)**
Mass values : Monoisotopic
Protein mass : Unrestricted
Peptide mass tolerance : ± 1.6 Da
Fragment mass tolerance : ±
: 0.8 Da
Max missed cleavages : 3
Instrument type : ESI-TRAP
Number of queries : 496

Score distribution

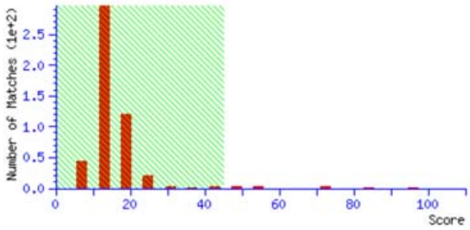

Peptide score distribution. Ions score is $-10\log(P)$, where P is the probability that the observed match is a random event.
There are **8** peptide matches above identity threshold and **11** matches above homology threshold for **496** queries. On average, individual ions scores **> 45** (beyond green shading) indicate **identity or extensive homology** (p<0.05).

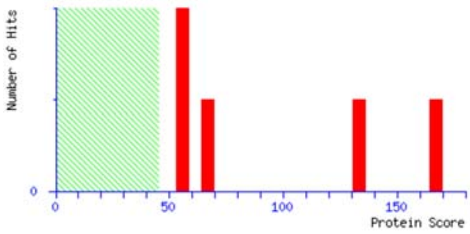

[Deprecated] Protein score distribution. Score distribution for family members in the first 50 proteins. Protein scores are derived from ions scores as a non-probabilistic basis for ranking protein families.

Legend

Protein Family Summary

Significance threshold p<	0.05	Max. number of families	20
Display non-sig. matches		Dendrograms cut at	0
Preferred taxonomy	All entries		

Sensitivity

Protein families 1–5 (out of 5)

10 per page 1

1 **P55260.3** 167 RecName: Full=Annexin A4; AltName: Full=36 kDa zymogen granule membrane-associated protein; Short=ZAP36; AltName: Full=Annex...

		Score	Mass	Matches	Sequences	emPAI
1.1	P55260.3	167	35826	4 (4)	4 (4)	0.62

RecName: Full=Annexin A4; AltName: Full=36 kDa zymogen granule membrane-associated protein; Short=ZAP36; AltName: Full=Annexin IV; AltName: Full=Annexin-4; AltName: Full=Lipoc...

4 peptide matches (4 non-duplicate, 0 duplicate)

Query	Dupes	Observed	Mr(expt)	Mr(calc)	Delta	M	Score	Expect	Rank	U	Peptide
196		574.2400	1146.4654	1146.5881	-0.1226	0	72	2.9e-05	1	U	K.GLGTDDSTLIR.V
288		700.0300	1398.0454	1397.6688	0.3767	0	56	8e-05	1	U	R.INQTYQQQYGR.S
326		775.2800	1548.5454	1548.7532	-0.2078	0	86	2e-07	1	U	K.AASGFNATEDAQVLR.K
358		833.3600	1664.7054	1665.8461	-1.1407	0	44	0.00048	1	U	K.SETSGSFEDALLAIVK.C

1 subset or intersection (1 subset protein in total)

2	NP_036767.1	132	anionic trypsin-1 precursor [Rattus norvegicus]
3	XP_008763925.1	70	PREDICTED: keratin, type II cytoskeletal 73 isoform X1 [Rattus norvegicus]
4	NP_001008825.1	61	keratin, type II cytoskeletal cochleal [Rattus norvegicus]
5	NP_001008751.1	57	keratin, type I cytoskeletal 14 [Rattus norvegicus]

10 per page 1

Not what you expected? Try **the peptide summary**.

Mascot: http://www.matrixscience.com/

MATRIX SCIENCE MASCOT Search Results

Protein View: P55260.3

RecName: Full=Annexin A4; AltName: Full=36 kDa zymogen granule membrane-associated protein; Short=ZAP36; AltName: Full=Annexin IV; AltName: Full=Annexin-4; AltName: Full=Lipocortin IV

**Database:**	NCBIprot
**Score:**	167
**Monoisotopic mass (M_r):**	35826
**Calculated pI:**	5.31
**Taxonomy:**	Rattus norvegicus

This protein sequence matches the following other entries:

- BAA07399.2 from Rattus norvegicus

Sequence similarity is available as an NCBI BLAST search of P55260.3 against nr.

Search parameters

**MS data file:**	G:\PRESTATIONS\Labos académiques\Internationaux\Etats Unis\Martina Rosenberg\Data pour publi M Rosenberg\Identifications Mascot\MR__2068.mgf
**Enzyme:**	Trypsin: cuts C-term side of KR unless next residue is P.
**Variable modifications:**	Carbamidomethyl (C), Oxidation (M)

Protein sequence coverage: 16%

Matched peptides shown in ***bold red***.

1 METKGGTVK**A ASGFNATEDA QVLR**KAMKGL GTDEDAIIGV LACRNTAQRQ
51 EIRTAYKSTI GRDLLEDLKS ELSSNFEQVI LGMMTPTVLY DVQELRRAMK
101 GAGTDEGCLI EILASRNPEE IRR**INQTYQQ QYGR**SLEEDI CSDTSFMFQR
151 VLVSLTAGGR DEGNYLDDAL VRQDAQDLYE AGEKRWGTDE VKFLSILCSR
201 NRNHLLHVFD EYKRISQKDI EQSIK**SETSG SFEDALLAIV K**CMRNKPAYF
251 AERLYKSMK**G LGTDDSTLIR** VMVSRAEIDM LDIPANFKRV YGKSLYSFIK
301 GDTSGDYRKV LLILCGGDD

Unformatted sequence string: 319 residues (for pasting into other applications).

Sort by ◉ residue number ○ increasing mass ○ decreasing mass
Show ◉ matched peptides only ○ predicted peptides also

Query	Start – End	Observed	Mr(expt)	Mr(calc)	Delta	M	Score	Expect	Rank	U	Peptide
326	10 – 24	775.2800	1548.5454	1548.7532	-0.2078	0	86	2e-07	1	U	K.AASGFNATEDAQVLR.K
288	124 – 134	700.0300	1398.0454	1397.6688	0.3767	0	56	8e-05	1	U	R.INQTYQQQYGR.S
358	226 – 241	833.3600	1664.7054	1665.8461	-1.1407	0	44	0.00048	1	U	K.SETSGSFEDALLAIVK.C
196	260 – 270	574.2400	1146.4654	1146.5881	-0.1226	0	72	2.9e-05	1	U	K.GLGTDDSTLIR.V

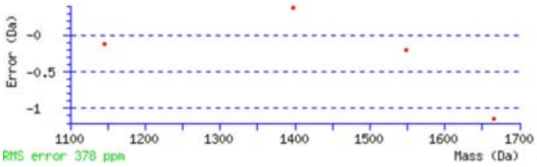

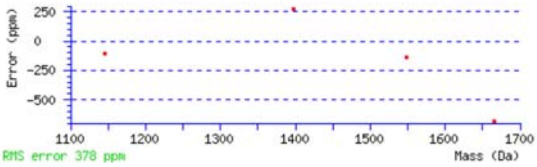


```
LOCUS       ANXA4_RAT                319 aa            linear   ROD 16-JAN-2019
DEFINITION  RecName: Full=Annexin A4; AltName: Full=36 kDa zymogen granule
            membrane-associated protein; Short=ZAP36; AltName: Full=Annexin IV;
            AltName: Full=Annexin-4; AltName: Full=Lipocortin IV.
ACCESSION   P55260
VERSION     P55260.3
DBSOURCE    UniProtKB: locus ANXA4_RAT, accession P55260;
            class: standard.
            created: Oct 1, 1996.
            sequence updated: Jan 23, 2007.
            annotation updated: Jan 16, 2019.
            xrefs: D38224.2, BAA07399.2, 2ZHI_A, 2ZHJ_A
            xrefs (non-sequence databases): UniGene:Rn.19270, PDBsum:2ZHI,
            PDBsum:2ZHJ, ProteinModelPortal:P55260, SMR:P55260,
            STRING:10116.ENSRNOP00000024436, iPTMnet:P55260,
            PhosphoSitePlus:P55260, jPOST:P55260, PaxDb:P55260, PRIDE:P55260,
            UCSC:RGD:621171, RGD:621171, eggNOG:KOG0819, eggNOG:ENOG410XPUN,
            HOGENOM:HOG000158803, HOVERGEN:HBG061815, InParanoid:P55260,
            PhylomeDB:P55260, EvolutionaryTrace:P55260, PRO:PR:P55260,
            Proteomes:UP000002494, GO:0005737, GO:0005829, GO:0005634,
            GO:0005886, GO:0005509, GO:0005544, GO:0048306, GO:0035374,
            GO:0008201, GO:0042802, GO:0030855, GO:0006887, GO:0007219,
            Gene3D:1.10.220.10, InterPro:IPR001464, InterPro:IPR018502,
            InterPro:IPR018252, InterPro:IPR037104, InterPro:IPR002391,
            PANTHER:PTHR10502:SF28, Pfam:PF00191, PRINTS:PR00196,
            SMART:SM00335, PROSITE:PS00223
KEYWORDS    3D-structure; Acetylation; Annexin; Calcium;
            Calcium/phospholipid-binding; Complete proteome; Phosphoprotein;
            Reference proteome; Repeat.
SOURCE      Rattus norvegicus (Norway rat)
  ORGANISM  Rattus norvegicus
            Eukaryota; Metazoa; Chordata; Craniata; Vertebrata; Euteleostomi;
            Mammalia; Eutheria; Euarchontoglires; Glires; Rodentia; Myomorpha;
            Muroidea; Muridae; Murinae; Rattus.
REFERENCE   1  (residues 1 to 319)
  AUTHORS   Fukuoka,S., Kern,H., Kazuki-Sugino,R. and Ikeda,Y.
  TITLE     Cloning and characterization of ZAP36, an annexin-like, zymogen
            granule membrane associated protein, in exocrine pancreas
  JOURNAL   Biochim. Biophys. Acta 1575 (1-3), 148-152 (2002)
   PUBMED   12020832
```

```
  REMARK    NUCLEOTIDE SEQUENCE [MRNA].;
            STRAIN=Wistar; TISSUE=Pancreas
COMMENT     On Oct 28, 2003 this sequence version replaced gi:1703320.
            [FUNCTION] Calcium/phospholipid-binding protein which promotes
            membrane fusion and is involved in exocytosis. {ECO:0000250}.
            [DOMAIN] A pair of annexin repeats may form one binding site for
            calcium and phospholipid.
            [MISCELLANEOUS] Seems to bind one calcium ion with high affinity.
            {ECO:0000250}.
            [SIMILARITY] Belongs to the annexin family. {ECO:0000305}.
FEATURES             Location/Qualifiers
     source          1..319
                     /organism="Rattus norvegicus"
                     /db_xref="taxon:10116"
     gene            1..319
                     /gene="Anxa4"
                     /gene_synonym="Anx4"
     Protein         1..319
                     /product="Annexin A4"
                     /note="36 kDa zymogen granule membrane-associated protein;
                     Annexin IV; Annexin-4; Lipocortin IV; ZAP36"
                     /UniProtKB_evidence="Evidence at protein level"
     Region          1..319
                     /region_name="Mature chain"
                     /experiment="experimental evidence, no additional details
                     recorded"
                     /note="Annexin A4. /FTId=PRO_0000067485."
     Site            7
                     /site_type="phosphorylation"
                     /experiment="experimental evidence, no additional details
                     recorded"
                     /note="Phosphothreonine. {ECO:0000250|UniProtKB:P08132}."
     Site            12
                     /site_type="phosphorylation"
                     /experiment="experimental evidence, no additional details
                     recorded"
                     /note="Phosphoserine. {ECO:0000250|UniProtKB:P09525}."
     Region          16..27
                     /region_name="Helical region"
                     /experiment="experimental evidence, no additional details
                     recorded"
                     /note="{ECO:0000244|PDB:2ZHJ}."
     Region          19..83
                     /region_name="Annexin"
                     /note="Annexin; pfam00191"
                     /db_xref="CDD:278615"
     Region          23..83
                     /region_name="Repetitive region"
                     /experiment="experimental evidence, no additional details
                     recorded"
                     /note="Annexin 1."
     Region          28..31
                     /region_name="Beta-strand region"
                     /experiment="experimental evidence, no additional details
                     recorded"
                     /note="{ECO:0000244|PDB:2ZHJ}."
     Region          34..41
                     /region_name="Helical region"
                     /experiment="experimental evidence, no additional details
                     recorded"
                     /note="{ECO:0000244|PDB:2ZHJ}."
     Region          46..60
                     /region_name="Helical region"
                     /experiment="experimental evidence, no additional details
                     recorded"
                     /note="{ECO:0000244|PDB:2ZHJ}."
     Region          64..71
                     /region_name="Helical region"
                     /experiment="experimental evidence, no additional details
                     recorded"
                     /note="{ECO:0000244|PDB:2ZHJ}."
     Region          74..84
                     /region_name="Helical region"
                     /experiment="experimental evidence, no additional details
                     recorded"
                     /note="{ECO:0000244|PDB:2ZHJ}."
     Region          87..99
                     /region_name="Helical region"
                     /experiment="experimental evidence, no additional details
                     recorded"
                     /note="{ECO:0000244|PDB:2ZHJ}."
     Region          90..155
                     /region_name="Annexin"
                     /note="Annexin; pfam00191"
                     /db_xref="CDD:278615"
     Region          95..155
                     /region_name="Repetitive region"
                     /experiment="experimental evidence, no additional details
                     recorded"
                     /note="Annexin 2."
     Region          100..102
                     /region_name="Beta-strand region"
                     /experiment="experimental evidence, no additional details
                     recorded"
                     /note="{ECO:0000244|PDB:2ZHJ}."
     Region          106..115
                     /region_name="Helical region"
                     /experiment="experimental evidence, no additional details
                     recorded"
                     /note="{ECO:0000244|PDB:2ZHJ}."
     Region          118..132
                     /region_name="Helical region"
                     /experiment="experimental evidence, no additional details
                     recorded"
                     /note="{ECO:0000244|PDB:2ZHJ}."
     Region          136..143
                     /region_name="Helical region"
                     /experiment="experimental evidence, no additional details
                     recorded"
                     /note="{ECO:0000244|PDB:2ZHJ}."
     Region          146..157
                     /region_name="Helical region"
                     /experiment="experimental evidence, no additional details
```

```
                     recorded"
                     /note="{ECO:0000244|PDB:2ZHJ}."
     Region          168..181
                     /region_name="Helical region"
                     /experiment="experimental evidence, no additional details
                     recorded"
                     /note="{ECO:0000244|PDB:2ZHJ}."
     Region          173..239
                     /region_name="Annexin"
                     /note="Annexin; pfam00191"
                     /db_xref="CDD:278615"
     Region          179..239
                     /region_name="Repetitive region"
                     /experiment="experimental evidence, no additional details
                     recorded"
                     /note="Annexin 3."
     Region          182..184
                     /region_name="Hydrogen bonded turn"
                     /experiment="experimental evidence, no additional details
                     recorded"
                     /note="{ECO:0000244|PDB:2ZHJ}."
     Region          185..187
                     /region_name="Beta-strand region"
                     /experiment="experimental evidence, no additional details
                     recorded"
                     /note="{ECO:0000244|PDB:2ZHJ}."
     Region          190..199
                     /region_name="Helical region"
                     /experiment="experimental evidence, no additional details
                     recorded"
                     /note="{ECO:0000244|PDB:2ZHJ}."
     Region          202..216
                     /region_name="Helical region"
                     /experiment="experimental evidence, no additional details
                     recorded"
                     /note="{ECO:0000244|PDB:2ZHJ}."
     Site            213
                     /site_type="acetylation"
                     /experiment="experimental evidence, no additional details
                     recorded"
                     /note="N6-acetyllysine. {ECO:0000250|UniProtKB:P09525}."
     Region          220..227
                     /region_name="Helical region"
                     /experiment="experimental evidence, no additional details
                     recorded"
                     /note="{ECO:0000244|PDB:2ZHJ}."
     Region          230..244
                     /region_name="Helical region"
                     /experiment="experimental evidence, no additional details
                     recorded"
                     /note="{ECO:0000244|PDB:2ZHJ}."
     Region          246..258
                     /region_name="Helical region"
                     /experiment="experimental evidence, no additional details
                     recorded"
                     /note="{ECO:0000244|PDB:2ZHJ}."
     Region          249..314
                     /region_name="Annexin"
                     /note="Annexin; pfam00191"
                     /db_xref="CDD:278615"
     Region          254..314
                     /region_name="Repetitive region"
                     /experiment="experimental evidence, no additional details
                     recorded"
                     /note="Annexin 4."
     Region          259..262
                     /region_name="Beta-strand region"
                     /experiment="experimental evidence, no additional details
                     recorded"
                     /note="{ECO:0000244|PDB:2ZHJ}."
     Region          265..275
                     /region_name="Helical region"
                     /experiment="experimental evidence, no additional details
                     recorded"
                     /note="{ECO:0000244|PDB:2ZHJ}."
     Region          276..278
                     /region_name="Hydrogen bonded turn"
                     /experiment="experimental evidence, no additional details
                     recorded"
                     /note="{ECO:0000244|PDB:2ZHJ}."
     Region          280..291
                     /region_name="Helical region"
                     /experiment="experimental evidence, no additional details
                     recorded"
                     /note="{ECO:0000244|PDB:2ZHJ}."
     Site            293
                     /site_type="acetylation"
                     /experiment="experimental evidence, no additional details
                     recorded"
                     /note="N6-acetyllysine. {ECO:0000250|UniProtKB:P09525}."
     Region          295..302
                     /region_name="Helical region"
                     /experiment="experimental evidence, no additional details
                     recorded"
                     /note="{ECO:0000244|PDB:2ZHJ}."
     Site            300
                     /site_type="acetylation"
                     /experiment="experimental evidence, no additional details
                     recorded"
                     /note="N6-acetyllysine. {ECO:0000250|UniProtKB:P09525}."
     Region          305..315
                     /region_name="Helical region"
                     /experiment="experimental evidence, no additional details
                     recorded"
                     /note="{ECO:0000244|PDB:2ZHJ}."
```

Mascot: http://www.matrixscience.com/

MATRIX SCIENCE MASCOT Search Results

**User**	: pbm
**E-mail**	:
**Search title**	: Submitted from MRosenberg Data2009 by Mascot Daemon on SCT-14-65A4
**MS data file**	: G:\PRESTATIONS\Labos académiques\Internationaux\Etats Unis\Martina Rosenberg\Data pour publi M Rosenberg\Identifications Mascot\MR_1990.mgf
**Database**	: NCBIprot 20171205 (139,213,787 sequences; 51,013,024,959 residues)
**Taxonomy**	: Rattus (77,467 sequences)
**Timestamp**	: 25 Jan 2019 at 10:26:10 GMT

Not what you expected? Try **the peptide summary**.

Search parameters

Score distribution

Legend

Protein Family Summary

Significance threshold p<	0.05	Max. number of families	20
Display non-sig. matches	☐	Dendrograms cut at	0
Preferred taxonomy	All entries		

Sensitivity

Protein families 1–5 (out of 5)

10 per page 1

1	**EDL86882.1**	92	rCG50690, partial [Rattus norvegicus]
2	**XP_008763925.1**	70	PREDICTED: keratin, type II cytoskeletal 73 isoform X1 [Rattus norvegicus]
3	**NP_036767.1**	61	anionic trypsin-1 precursor [Rattus norvegicus]
4	**NP_542152.1**	58	alpha-soluble NSF attachment protein [Rattus norvegicus]

		Score	Mass	Matches	Sequences	emPAI
4.1	NP_542152.1	58	33171	1 (1)	1 (1)	0.14

alpha-soluble NSF attachment protein [Rattus norvegicus]

1 sameset of NP_542152.1

1 peptide matches (1 non-duplicate, 0 duplicate)

Query	Dupes	Observed	Mr(expt)	Mr(calc)	Delta	M	Score	Expect	Rank	U	Peptide
322		734.8300	1467.6454	1467.7358	-0.0903	0	58	0.00045	1	U	K.VAGYAAQLEQYQK.A

5	**EDM05185.1**	32	similar to mKIAA0664 protein (predicted), isoform CRA_b, partial [Rattus norvegicus]

10 per page 1

Not what you expected? Try **the peptide summary**.

Mascot: http://www.matrixscience.com/

MATRIX SCIENCE MASCOT Search Results

Protein View: NP_542152.1

alpha-soluble NSF attachment protein [Rattus norvegicus]

**Database:**	NCBIprot
**Score:**	58
**Monoisotopic mass (M_r):**	33171
**Calculated pI:**	5.30
**Taxonomy:**	Rattus norvegicus

This protein sequence matches the following other entries:

- XP_005086504.1 from **Mesocricetus auratus**
- XP_012980973.1 from **Mesocricetus auratus**
- XP_021074482.1 from **Mus pahari**
- P54921.2 from **Rattus norvegicus**
- CAA62005.1 from **Rattus norvegicus**
- AAH63156.1 from **Rattus norvegicus**
- EDM08339.1 from **Rattus norvegicus**

Sequence similarity is available as **an NCBI BLAST search of NP_542152.1 against nr**.

Search parameters

**MS data file:**	G:\PRESTATIONS\Labos académiques\Internationaux\Etats Unis\Martina Rosenberg\Data pour publi M Rosenberg\Identifications Mascot\MR_1990.mgf
**Enzyme:**	Trypsin: cuts C-term side of KR unless next residue is P.
**Variable modifications:**	**Carbamidomethyl (C)**, **Oxidation (M)**

Protein sequence coverage: 4%

Matched peptides shown in ***bold red***.

```
  1 MDTSGKQAEA MALLAEAERK VKNSQSFFSG LFGGSSKIEE ACEIYARAAN
 51 MFKMAKNWSA AGNAFCQAAQ LHLQLQSKHD AATCFVDAGN AFKKADPQEA
101 INCLMRAIEI YTDMGRFTIA AKHHISIAEI YETELVDVEK AIAHYEQSAD
151 YYKGEESNSS ANKCLLKVAG YAAQLEQYQK AIDIYEQVGT SAMDSPLLKY
201 SAKDYFFKAA LCHFCIDMLN AKLAVQKYEE LFPAFSDSRE CKLMKKLLEA
251 HEEQNVDSYT ESVKEYDSIS RLDQWLTTML LRIKKTIQGD EEDLR
```

Unformatted sequence string: **295 residues** (for pasting into other applications).

Sort by ◉ residue number ○ increasing mass ○ decreasing mass
Show ◉ matched peptides only ○ predicted peptides also

Query	Start – End	Observed	Mr(expt)	Mr(calc)	Delta	M	Score	Expect	Rank	U	Peptide
322	168 – 180	734.8300	1467.6454	1467.7358	-0.0903	0	58	0.00045	1	U	K.VAGYAAQLEQYQK.A

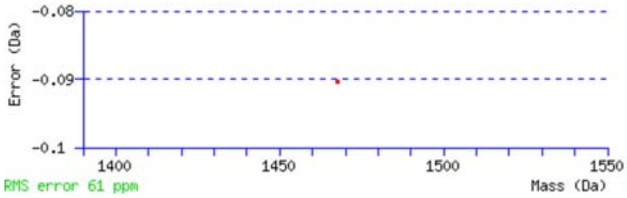

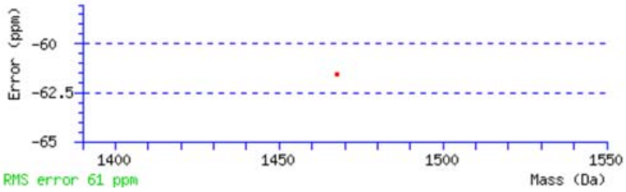

```
LOCUS       NP_542152                295 aa            linear   ROD 20-OCT-2018
DEFINITION  alpha-soluble NSF attachment protein [Rattus norvegicus].
ACCESSION   NP_542152
VERSION     NP_542152.1
DBSOURCE    REFSEQ: accession NM_080585.1
KEYWORDS    RefSeq.
SOURCE      Rattus norvegicus (Norway rat)
  ORGANISM  Rattus norvegicus
            Eukaryota; Metazoa; Chordata; Craniata; Vertebrata; Euteleostomi;
            Mammalia; Eutheria; Euarchontoglires; Glires; Rodentia; Myomorpha;
            Muroidea; Muridae; Murinae; Rattus.
REFERENCE   1  (residues 1 to 295)
  AUTHORS   Xi Z, Deng W, Wang L, Xiao F, Li J, Wang Z, Wang X, Mi X, Wang N
            and Wang X.
  TITLE     Association of Alpha-Soluble NSF Attachment Protein with Epileptic
            Seizure
  JOURNAL   J. Mol. Neurosci. 57 (3), 417-425 (2015)
   PUBMED   26156199
  REMARK    GeneRIF: alphaSNAP expression showed no change in
            pilocarpine-induced epileptic rats in the acute phase, but in the
            chronic phase levels were lower. siRNA decreased the time of
            latency to seizure and increased the incidence of chronic
            idiopathic epilepsy seizures.
```

```
REFERENCE   2  (residues 1 to 295)
  AUTHORS   Chen D, Li L, Yan J, Yang X, You Y, Zhou Y and Ling X.
  TITLE     The loss of alphaSNAP downregulates the expression of occludin in
            the intestinal epithelial cell of acute pancreatitis model
  JOURNAL   Pancreatology 14 (5), 347-355 (2014)
   PUBMED   25278303
  REMARK    GeneRIF: In severe acute pancreatitis, downregulated expression of
            alphaSNAP in intestinal epithelial cells leads to reduced occludin
            expression, apoptosis of intestinal epithelial cells, and increased
            permeability of the intestinal barrier.
REFERENCE   3  (residues 1 to 295)
  AUTHORS   Wilhelm BG, Mandad S, Truckenbrodt S, Krohnert K, Schafer C,
            Rammner B, Koo SJ, Classen GA, Krauss M, Haucke V, Urlaub H and
            Rizzoli SO.
  TITLE     Composition of isolated synaptic boutons reveals the amounts of
            vesicle trafficking proteins
  JOURNAL   Science 344 (6187), 1023-1028 (2014)
   PUBMED   24876496
REFERENCE   4  (residues 1 to 295)
  AUTHORS   Vivona S, Cipriano DJ, O'Leary S, Li YH, Fenn TD and Brunger AT.
  TITLE     Disassembly of all SNARE complexes by N-ethylmaleimide-sensitive
            factor (NSF) is initiated by a conserved 1:1 interaction between
            alpha-soluble NSF attachment protein (SNAP) and SNARE complex
  JOURNAL   J. Biol. Chem. 288 (34), 24984-24991 (2013)
   PUBMED   23836889
  REMARK    GeneRIF: a model of NSF-mediated disassembly in which the reaction
            is initiated by a 1:1 interaction between alpha-SNAP and the
            ternary SNARE complex, followed by NSF binding.
REFERENCE   5  (residues 1 to 295)
  AUTHORS   Burgalossi A, Jung S, Meyer G, Jockusch WJ, Jahn O, Taschenberger
            H, O'Connor VM, Nishiki T, Takahashi M, Brose N and Rhee JS.
  TITLE     SNARE protein recycling by alphaSNAP and betaSNAP supports synaptic
            vesicle priming
  JOURNAL   Neuron 68 (3), 473-487 (2010)
   PUBMED   21040848
  REMARK    Erratum:[Neuron. 2012 Feb 9;73(3):620]
REFERENCE   6  (residues 1 to 295)
  AUTHORS   Wong SH, Zhang T, Xu Y, Subramaniam VN, Griffiths G and Hong W.
  TITLE     Endobrevin, a novel synaptobrevin/VAMP-like protein preferentially
            associated with the early endosome
  JOURNAL   Mol. Biol. Cell 9 (6), 1549-1563 (1998)
   PUBMED   9614193
REFERENCE   7  (residues 1 to 295)
  AUTHORS   Hanson PI, Roth R, Morisaki H, Jahn R and Heuser JE.
  TITLE     Structure and conformational changes in NSF and its membrane
            receptor complexes visualized by quick-freeze/deep-etch electron
            microscopy
  JOURNAL   Cell 90 (3), 523-535 (1997)
   PUBMED   9267032
REFERENCE   8  (residues 1 to 295)
  AUTHORS   Bock JB, Klumperman J, Davanger S and Scheller RH.
  TITLE     Syntaxin 6 functions in trans-Golgi network vesicle trafficking
  JOURNAL   Mol. Biol. Cell 8 (7), 1261-1271 (1997)
   PUBMED   9243506
REFERENCE   9  (residues 1 to 295)
  AUTHORS   McMahon HT, Missler M, Li C and Sudhof TC.
  TITLE     Complexins: cytosolic proteins that regulate SNAP receptor function
  JOURNAL   Cell 83 (1), 111-119 (1995)
   PUBMED   7553862
REFERENCE   10 (residues 1 to 295)
  AUTHORS   Schmidt,D.D. and Messner,E.
  TITLE     The female hysterical personality disorder
  JOURNAL   J Fam Pract 4 (3), 573-577 (1977)
   PUBMED   845572
COMMENT     PROVISIONAL REFSEQ: This record has not yet been subject to final
            NCBI review. The reference sequence was derived from X89968.1.

            Summary: human homolog is involved in intra-Golgi transport [RGD,
            Feb 2006].

            Publication Note:  This RefSeq record includes a subset of the
            publications that are available for this gene. Please see the Gene
            record to access additional publications.

            ##Evidence-Data-START##
            Transcript exon combination :: X89968.1, BC063156.1 [ECO:0000332]
            RNAseq introns              :: single sample supports all introns
                                           SAMD00052296, SAMD00052297
                                           [ECO:0000348]
            ##Evidence-Data-END##
FEATURES             Location/Qualifiers
     source          1..295
                     /organism="Rattus norvegicus"
                     /strain="W"
                     /db_xref="taxon:10116"
                     /chromosome="1"
                     /map="1q21"
     Protein         1..295
                     /product="alpha-soluble NSF attachment protein"
                     /note="SNAP-alpha; N-ethylmaleimide sensitive fusion
                     protein attachment protein alpha;
                     N-ethylmaleimide-sensitive factor attachment protein,
                     alpha"
                     /calculated_mol_wt=33062
     Site            1
                     /site_type="other"
                     /experiment="experimental evidence, no additional details
```

```
                     recorded"
                     /note="N-acetylmethionine. {ECO:0000250|UniProtKB:P54920};
                     propagated from UniProtKB/Swiss-Prot (P54921.2)"
     Region          26..287
                     /region_name="SNAP"
                     /note="Soluble N-ethylmaleimide-sensitive factor (NSF)
                     Attachment Protein family; cd15832"
                     /db_xref="CDD:276937"
     Site            26
                     /site_type="other"
                     /experiment="experimental evidence, no additional details
                     recorded"
                     /note="Phosphoserine. {ECO:0000250|UniProtKB:Q9DB05};
                     propagated from UniProtKB/Swiss-Prot (P54921.2)"
     Site            29
                     /site_type="other"
                     /experiment="experimental evidence, no additional details
                     recorded"
                     /note="Phosphoserine. {ECO:0000250|UniProtKB:P54920};
                     propagated from UniProtKB/Swiss-Prot (P54921.2)"
     Region          37..74
                     /region_name="TPR repeat"
                     /note="TPR repeat [structural motif]"
                     /db_xref="CDD:276937"
     Region          78..114
                     /region_name="TPR repeat"
                     /note="TPR repeat [structural motif]"
                     /db_xref="CDD:276937"
     Region          117..154
                     /region_name="TPR repeat"
                     /note="TPR repeat [structural motif]"
                     /db_xref="CDD:276937"
     Region          158..194
                     /region_name="TPR repeat"
                     /note="TPR repeat [structural motif]"
                     /db_xref="CDD:276937"
     Site            195
                     /site_type="other"
                     /experiment="experimental evidence, no additional details
                     recorded"
                     /note="Phosphoserine. {ECO:0000250|UniProtKB:P54920};
                     propagated from UniProtKB/Swiss-Prot (P54921.2)"
     CDS             1..295
                     /gene="Napa"
                     /gene_synonym="alpha-SNAP"
                     /coded_by="NM_080585.1:46..933"
                     /db_xref="GeneID:140673"
                     /db_xref="RGD:620855"
```

Mascot: http://www.matrixscience.com/

MATRIX SCIENCE MASCOT Search Results

User : pbm
E-mail :
Search title : Submitted from MRosenberg Data2009 by Mascot Daemon on SCT-14-65A4
MS data file : G:\PRESTATIONS\Labos académiques\Internationaux\Etats Unis\Martina Rosenberg\Data pour publi M Rosenberg\Identifications Mascot\MR__2950.mgf
Database : NCBIprot 20171205 (139,213,787 sequences; 51,013,024,959 residues)
Taxonomy : Rattus (77,467 sequences)
Timestamp : 25 Jan 2019 at 10:12:25 GMT

Not what you expected? Try **the peptide summary**.

Search parameters
Score distribution
Modification statistics
Legend

Protein Family Summary

Significance threshold p< 0.05 Max. number of families 20
Display non-sig. matches Dendrograms cut at 0
Preferred taxonomy All entries

Sensitivity

Protein families 1–8 (out of 8)

10 per page 1

1	XP_008763925.1	69	PREDICTED: keratin, type II cytoskeletal 73 isoform X1 [Rattus norvegicus]
2	EDM12437.1	61	rCG47227, isoform CRA_a [Rattus norvegicus]

		Score	Mass	Matches	Sequences	emPAI
2.1	EDM12437.1	61	66079	3 (3)	3 (3)	0.22
	rCG47227, isoform CRA_a [Rattus norvegicus]					
	**2 samesets of EDM12437.1**					
	NP_446200.1	61	82987	3 (3)	3 (3)	
	dipeptidyl peptidase 3 [Rattus norvegicus]					
	XP_017444144.1	61	70685	3 (3)	3 (3)	
	PREDICTED: dipeptidyl peptidase 3 isoform X2 [Rattus norvegicus]					

3 peptide matches (3 non-duplicate, 0 duplicate)

Query	Dupes	Observed	Mr(expt)	Mr(calc)	Delta	M	Score	Expect	Rank	U	Peptide
177		536.3900	1070.7654	1070.6335	0.1319	0	37	0.004	1	U	R.LVASAEQLLK.E
179		538.6100	1612.8082	1611.7430	1.0652	0	35	0.014	1	U	K.SYEFQGNHFQVTR.G
409		944.4100	1886.8054	1886.9837	-0.1782	0	43	0.0013	1	U	R.LASVLNTEPALDSELTSK.L

3	NP_036767.1	58	anionic trypsin-1 precursor [Rattus norvegicus]
4	Q62871.1	43	RecName: Full=Cytoplasmic dynein 1 intermediate chain 2; AltName: Full=Cytoplasmic dynein intermediate chain 2; AltName: Full=Dyne...
5	XP_008770740.1	39	PREDICTED: nuclear factor of activated T-cells, cytoplasmic 3 isoform X1 [Rattus norvegicus]
6	NP_001008751.1	38	keratin, type I cytoskeletal 14 [Rattus norvegicus]
7	EDM06524.1	33	similar to hypothetical protein D11Ertd636e (predicted), isoform CRA_a [Rattus norvegicus]
8	Q9QW30.1	25	RecName: Full=Neurogenic locus notch homolog protein 2; Short=Notch 2; Contains: RecName: Full=Notch 2 extracellular truncation; C...

10 per page 1

Not what you expected? Try **the peptide summary**.

Mascot: http://www.matrixscience.com/

MATRIX SCIENCE MASCOT Search Results

Protein View: NP_446200.1

dipeptidyl peptidase 3 [Rattus norvegicus]

**Database:**	NCBIprot
**Score:**	61
**Monoisotopic mass (M_r):**	82987
**Calculated pI:**	5.12
**Taxonomy:**	Rattus norvegicus

This protein sequence matches the following other entries:

- XP_006230695.1 from **Rattus norvegicus**
- O55096.2 from **Rattus norvegicus**
- BAA24608.2 from **Rattus norvegicus**
- AAI07674.1 from **Rattus norvegicus**

Sequence similarity is available as **an NCBI BLAST search of NP_446200.1 against nr**.

Search parameters

**MS data file:**	G:\PRESTATIONS\Labos académiques\Internationaux\Etats Unis\Martina Rosenberg\Data pour publi M Rosenberg\Identifications Mascot\MR__2950.mgf
**Enzyme:**	Trypsin: cuts C-term side of KR unless next residue is P.
**Variable modifications:**	Carbamidomethyl (C), Oxidation (M)

Protein sequence coverage: 5%

Matched peptides shown in ***bold red***.

```
  1 MADTQYILPN DIGVSSLDCR EAFRLLSPTE RLYAHHLSRA AWYGGLAVLL
 51 QTSPEAPYIY ALLSRLFRAQ DPDQLRQHAL AEGLTEEEYQ AFLVYAAGVY
101 SNMGNYKSFG DTKFVPNLPK EKLERVILGS KAAQQHPEEV RSLWQTCGEL
151 MFSLEPRLRH LGLGKEGVTT YFSGDCAMED AKLAQDFLDS QNLSAYNTRL
201 FKVVGQEGKY HYEVRLASVL NTEPALDSEL TSKLKSYEFQ GNHFQVTRGD
251 YAPILQKVVE HLEKAKAYAA NSHQEQMLAQ YVESFTQGSI EAHKRGSRFW
301 IQDKGPIVES YIGFIESYRD PFGSRGEFEG FVAMVNKDMS AKFERLVASA
351 EQLLKELPWP PAFEKDKFLT PDFTSLDVLT FAGSGIPAGI NIPNYDDLRQ
401 TEGFKNVSLG NVLAVAYATK REKLTFMEEE DKDLYIRWKG PSFDVQVGLH
451 ELLGHGSGKL FVQDEKGAFN FDQETVINPE TGEQIQSWYR SGETWDSKFS
501 TIASSYEECR AESVGLYLCL NPQVLQIFGF EGTDAEDVIY VNWLNMVRAG
551 LLALEFYTPE TANWRQAHMQ ARFVILRVLL EAGEGLVTVT PTTGSDGRPD
601 ARVHLDRSKI RSVGKPALER FLRRLQVLKS TGDVVAGRAL YEGYAAVTDA
651 PPECFLTLRD TVLLRKESRK LIVQPNTRLE GSEVQLVEYE ASAAGLIRSF
701 CERFPEDGPE LEEVLTQLAT ADAQFWRDQV QEAPSGQA
```

Unformatted sequence string: **738 residues** (for pasting into other applications).

Sort by ◉ residue number ○ increasing mass ○ decreasing mass
Show ◉ matched peptides only ○ predicted peptides also

Query	Start – End	Observed	Mr(expt)	Mr(calc)	Delta	M	Score	Expect	Rank	U	Peptide
409	216 – 233	944.4100	1886.8054	1886.9837	-0.1782	0	43	0.0013	1	U	R.LASVLNTEPALDSELTSK.L
179	236 – 248	538.6100	1612.8082	1611.7430	1.0652	0	35	0.014	1	U	K.SYEFQGNHFQVTR.G
177	346 – 355	536.3900	1070.7654	1070.6335	0.1319	0	37	0.004	1	U	R.LVASAEQLLK.E

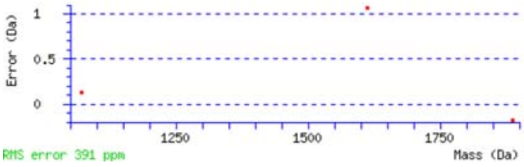

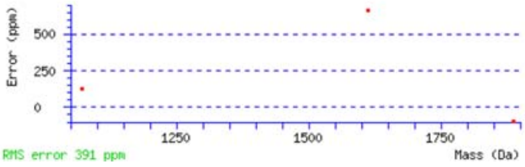

```
LOCUS       NP_446200                738 aa            linear   ROD 11-NOV-2018
DEFINITION  dipeptidyl peptidase 3 [Rattus norvegicus].
ACCESSION   NP_446200 XP_341989
VERSION     NP_446200.1
DBSOURCE    REFSEQ: accession NM_053748.1
KEYWORDS    RefSeq.
SOURCE      Rattus norvegicus (Norway rat)
  ORGANISM  Rattus norvegicus
            Eukaryota; Metazoa; Chordata; Craniata; Vertebrata; Euteleostomi;
            Mammalia; Eutheria; Euarchontoglires; Glires; Rodentia; Myomorpha;
            Muroidea; Muridae; Murinae; Rattus.
REFERENCE   1  (residues 1 to 738)
  AUTHORS   Hayes JD and Dinkova-Kostova AT.
  TITLE     The Nrf2 regulatory network provides an interface between redox and
            intermediary metabolism
  JOURNAL   Trends Biochem. Sci. 39 (4), 199-218 (2014)
   PUBMED   24647116
  REMARK    Review article
REFERENCE   2  (residues 1 to 738)
  AUTHORS   Hirose J, Hata T, Kawaoka C, Ikeura T, Kitahara S, Horii K, Tomida
            H, Iwamoto H, Ono Y and Fukasawa KM.
  TITLE     Flexibility of the coordination geometry around the cupric ions in
            Cu(II)-rat dipeptidyl peptidase III is important for the expression
            of enzyme activity
  JOURNAL   Arch. Biochem. Biophys. 525 (1), 71-81 (2012)
   PUBMED   22683474
  REMARK    GeneRIF: structure and activity of Cu(II)-rat dipeptidyl peptidase
            III
```

```
REFERENCE   3  (residues 1 to 738)
  AUTHORS   Buschow SI, van Balkom BW, Aalberts M, Heck AJ, Wauben M and
            Stoorvogel W.
  TITLE     MHC class II-associated proteins in B-cell exosomes and potential
            functional implications for exosome biogenesis
  JOURNAL   Immunol. Cell Biol. 88 (8), 851-856 (2010)
   PUBMED   20458337
REFERENCE   4  (residues 1 to 738)
  AUTHORS   Cicin-Sain L, Simaga S, Froebe A and Abramic M.
  TITLE     Central aminopeptidase and serotonin system activities: possible
            relationship
  JOURNAL   Neuropeptides 42 (4), 435-440 (2008)
   PUBMED   18547641
  REMARK    GeneRIF: Results suggest that the upregulation of serotonergic
            activity may be related to a lowered brain dipeptidyl peptidase
            III, alanyl- and arginyl aminopeptidase activity which may have an
            influence on the cleavage of their physiological substrates.
REFERENCE   5  (residues 1 to 738)
  AUTHORS   Abramic M, Schleuder D, Dolovcak L, Schroder W, Strupat K, Sagi D,
            Peter-Katalini J and Vitale L.
  TITLE     Human and rat dipeptidyl peptidase III: biochemical and mass
            spectrometric arguments for similarities and differences
  JOURNAL   Biol. Chem. 381 (12), 1233-1243 (2000)
   PUBMED   11209758
REFERENCE   6  (residues 1 to 738)
  AUTHORS   Li YH, Maeda T, Yamane T and Ohkubo I.
  TITLE     Alteration of rat dipeptidyl peptidase III by site-directed
            mutagenesis: cysteine(176) is a regulatory residue for the enzyme
            activity
  JOURNAL   Biochem. Biophys. Res. Commun. 276 (2), 553-558 (2000)
   PUBMED   11027512
REFERENCE   7  (residues 1 to 738)
  AUTHORS   Fukasawa K, Fukasawa KM, Iwamoto H, Hirose J and Harada M.
  TITLE     The HELLGH motif of rat liver dipeptidyl peptidase III is involved
            in zinc coordination and the catalytic activity of the enzyme
  JOURNAL   Biochemistry 38 (26), 8299-8303 (1999)
   PUBMED   10387075
REFERENCE   8  (residues 1 to 738)
  AUTHORS   Fukasawa K, Fukasawa KM, Kanai M, Fujii S, Hirose J and Harada M.
  TITLE     Dipeptidyl peptidase III is a zinc metallo-exopeptidase. Molecular
            cloning and expression
  JOURNAL   Biochem. J. 329 (Pt 2), 275-282 (1998)
   PUBMED   9425109
COMMENT     PROVISIONAL REFSEQ: This record has not yet been subject to final
            NCBI review. The reference sequence was derived from D89340.2.
            On Dec 17, 2003 this sequence version replaced XP_341989.1.

            Summary: catalyzes the release of an N-terminal dipeptide from a
            peptide of four or more residues; catalyzes the hydrolysis of
            Arg-Arg-2-naphthylamide; binds zinc [RGD, Feb 2006].

            ##Evidence-Data-START##
            Transcript exon combination :: D89340.2, BC107673.1 [ECO:0000332]
            RNAseq introns              :: mixed/partial sample support
                                           SAMD00052296, SAMD00052297
                                           [ECO:0000350]
            ##Evidence-Data-END##
FEATURES             Location/Qualifiers
     source          1..738
                     /organism="Rattus norvegicus"
                     /strain="W"
                     /db_xref="taxon:10116"
                     /chromosome="1"
                     /map="1q43"
     Protein         1..738
                     /product="dipeptidyl peptidase 3"
                     /EC_number="3.4.14.4"
                     /function="hydrolase"
                     /note="dipeptidyl-peptidase 3; DPP III; dipeptidyl
                     arylamidase III; dipeptidyl aminopeptidase III; dipeptidyl
                     peptidase III; dipeptidylpeptidase III; enkephalinase B"
                     /calculated_mol_wt=82908
     Site            2
                     /site_type="other"
                     /experiment="experimental evidence, no additional details
                     recorded"
                     /note="N-acetylalanine. {ECO:0000250|UniProtKB:Q9NY33};
                     propagated from UniProtKB/Swiss-Prot (O55096.2)"
     Region          147..704
                     /region_name="Peptidase_M49"
                     /note="Peptidase family M49; pfam03571"
                     /db_xref="CDD:281557"
     CDS             1..738
                     /gene="Dpp3"
                     /coded_by="NM_053748.1:15..2231"
                     /db_xref="GeneID:114591"
                     /db_xref="RGD:621127"
```

Mascot: http://www.matrixscience.com/

MATRIX SCIENCE MASCOT Search Results

User : pbm
E-mail :
Search title : Submitted from MRosenberg Data2009 by Mascot Daemon on SCT-14-65A4
MS data file : G:\PRESTATIONS\Labos académiques\Internationaux\Etats Unis\Martina Rosenberg\Data pour publi M Rosenberg\Identifications Mascot\MR_1016.mgf
Database : NCBIprot 20171205 (139,213,787 sequences; 51,013,024,959 residues)
Taxonomy : Rattus (77,467 sequences)
Timestamp : 25 Jan 2019 at 10:14:03 GMT

Not what you expected? Try **the peptide summary**.

- **Search parameters**
- **Score distribution**
- **Modification statistics**
- **Legend**

Protein Family Summary

Significance threshold p<	0.05	Max. number of families	20
Display non-sig. matches		Dendrograms cut at	0
Preferred taxonomy	All entries		

Sensitivity

Protein families 1–7 (out of 7)

10 per page 1

1	NP_036767.1	79	anionic trypsin-1 precursor [Rattus norvegicus]
2	XP_008770740.1	40	PREDICTED: nuclear factor of activated T-cells, cytoplasmic 3 isoform X1 [Rattus norvegicus]
3	XP_008759890.2	40	PREDICTED: laminin subunit gamma-3 isoform X1 [Rattus norvegicus]
4	Q9JII4.1	33	RecName: Full=Prolactin-5A1; AltName: Full=Placental prolactin-like protein L; Short=PLP-L; Short=PRL-like protein L; Flags: Precursor
5	Q62871.1	32	RecName: Full=Cytoplasmic dynein 1 intermediate chain 2; AltName: Full=Cytoplasmic dynein intermediate chain 2; AltName: Full=Dyne...

		Score	Mass	Matches	Sequences	emPAI
5.1	Q62871.1	32	71134	1 (1)	1 (1)	0.06

RecName: Full=Cytoplasmic dynein 1 intermediate chain 2; AltName: Full=Cytoplasmic dynein intermediate chain 2; AltName: Full=Dynein intermediate chain 2, cytosolic; Short=DH IC-2

5 samesets of Q62871.1

1 peptide matches (1 non-duplicate, 0 duplicate)

Query	Dupes	Observed	Mr(expt)	Mr(calc)	Delta	M	Score	Expect	Rank	U	Peptide
432		976.0000	1949.9854	1949.8563	0.1292	0	32	0.022	1	U	K.SVSTPSEAGSQDSGDGAVGSR.T

6	NP_001008751.1	31	keratin, type I cytoskeletal 14 [Rattus norvegicus]
7	EDM13863.1	30	similar to 2210403N09Rik protein (predicted), isoform CRA_a [Rattus norvegicus]

10 per page 1

Not what you expected? Try **the peptide summary**.

Mascot: http://www.matrixscience.com/

MATRIX SCIENCE MASCOT Search Results

Protein View: Q62871.1

RecName: Full=Cytoplasmic dynein 1 intermediate chain 2; AltName: Full=Cytoplasmic dynein intermediate chain 2; AltName: Full=Dynein intermediate chain 2, cytosolic; Short=DH IC-2

**Database:**	NCBIprot
**Score:**	32
**Monoisotopic mass (M_r):**	71134
**Calculated pI:**	5.11
**Taxonomy:**	Rattus norvegicus

This protein sequence matches the following other entries:

- AAA89163.1 from **Rattus norvegicus**

Sequence similarity is available as **an NCBI BLAST search of Q62871.1 against nr**.

Search parameters

**MS data file:**	G:\PRESTATIONS\Labos académiques\Internationaux\Etats Unis\Martina Rosenberg\Data pour publi M Rosenberg\Identifications Mascot\MR_1016.mgf
**Enzyme:**	Trypsin: cuts C-term side of KR unless next residue is P.
**Variable modifications:**	**Carbamidomethyl (C)**, **Oxidation (M)**

Protein sequence coverage: 3%

Matched peptides shown in ***bold red***.

```
  1 MSDKSELKAE LERKKQRLAQ IREEKKRKEE ERKKKETDQK KEAAVSVQEE
 51 SDLEKKRREA EALLQSMGLT TDSPIVFSEH WVPPPMSPSS KSVSTPSEAG
101 SQDSGDGAVG SRTLHWDTDP SALQLHSDSD LGRGPIKLGM AKITQVDFPP
151 REIVTYTKET QTPVTAQPKE DEEEEDDVAA PKPPVEPEEE KILKKDEEND
201 SKAPPHELTE EEKQQILHSE EFLSFFDHST RIVERALSEQ INIFFDYSGR
251 DLEDKEGEIQ AGAKLSLNRQ FFDERWSKHR VVSCLDWSSQ YPELLVASYN
301 NNEEAPHEPD GVALVWNMKY KKTTPEYVFH CQSAVMSATF AKFHPNLVVG
351 GTYSGQIVLW DNRSNKRTPV QRTPLSAAAH THPVYCVNVV GTQNAHNLIS
401 ISTDGKICSW SLDMLSHPQD SMELVHKQSK AVAVTSMSFP VGDVNNFVVG
451 SEEGSVYTAC RHGSKAGISE MFEGHQGPIT GIHCHAAVGA VDFSHLFVTS
501 SFDWTVKLWS TKNNKPLYSF EDNSDYVYDV IGSPTHPALF ACVDGMGRLD
551 LWNLNNDTEV PTASISVEGN PALNRVRWTH SGREIAVGDS EGQIVIYDVG
601 EQIAVPRNDE WARFGRTLAE INASRADAEE EAATRIPA
```

Unformatted sequence string: **638 residues** (for pasting into other applications).

Sort by ◉ residue number ○ increasing mass ○ decreasing mass
Show ◉ matched peptides only ○ predicted peptides also

Query	Start – End	Observed	Mr(expt)	Mr(calc)	Delta	M	Score	Expect	Rank	U	Peptide
432	92 – 112	976.0000	1949.9854	1949.8563	0.1292	0	32	0.022	1	U	K.SVSTPSEAGSQDSGDGAVGSR.T

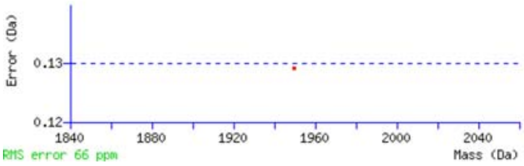

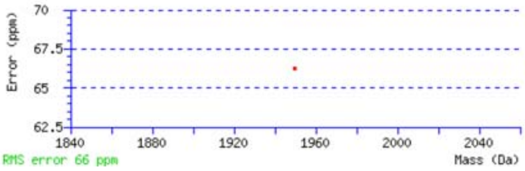


```
LOCUS       DC1I2_RAT                638 aa            linear   ROD 16-JAN-2019
DEFINITION  RecName: Full=Cytoplasmic dynein 1 intermediate chain 2; AltName:
            Full=Cytoplasmic dynein intermediate chain 2; AltName: Full=Dynein
            intermediate chain 2, cytosolic; Short=DH IC-2.
ACCESSION   Q62871
VERSION     Q62871.1
DBSOURCE    UniProtKB: locus DC1I2_RAT, accession Q62871;
            class: standard.
            extra accessions:Q62872,Q62873
            created: Nov 1, 1997.
            sequence updated: Nov 1, 1997.
            annotation updated: Jan 16, 2019.
            xrefs: U39044.1, AAA89163.1, U39045.1, AAA89164.1, U39046.1,
            AAA89165.1, 2PG1_I, 2PG1_J, 2PG1_K, 2PG1_L
            xrefs (non-sequence databases): UniGene:Rn.11014, PDBsum:2PG1,
            ProteinModelPortal:Q62871, SMR:Q62871, CORUM:Q62871,
            DIP:DIP-36880N, IntAct:Q62871, STRING:10116.ENSRNOP00000060919,
            iPTMnet:Q62871, PhosphoSitePlus:Q62871, jPOST:Q62871, PaxDb:Q62871,
            PeptideAtlas:Q62871, PRIDE:Q62871, UCSC:RGD:620174, RGD:620174,
            eggNOG:KOG1587, eggNOG:ENOG410XQ99, HOGENOM:HOG000116383,
            HOVERGEN:HBG004083, InParanoid:Q62871, PhylomeDB:Q62871,
            EvolutionaryTrace:Q62871, PRO:PR:Q62871, Proteomes:UP000002494,
            GO:0005737, GO:0005868, GO:0030286, GO:0005874, GO:0031982,
            GO:0045504, GO:0045503, GO:0003774, GO:0044877, GO:0007420,
            GO:1990090, GO:0007018, GO:0010977, Gene3D:2.130.10.10,
            InterPro:IPR025956, InterPro:IPR015943, InterPro:IPR001680,
            InterPro:IPR017986, InterPro:IPR036322, Pfam:PF11540, Pfam:PF00400,
            SMART:SM00320, SUPFAM:SSF50978, PROSITE:PS50082, PROSITE:PS50294
KEYWORDS    3D-structure; Acetylation; Alternative splicing; Complete proteome;
            Cytoplasm; Cytoskeleton; Dynein; Microtubule; Motor protein;
            Phosphoprotein; Reference proteome; Repeat; Transport; WD repeat.
SOURCE      Rattus norvegicus (Norway rat)
  ORGANISM  Rattus norvegicus
            Eukaryota; Metazoa; Chordata; Craniata; Vertebrata; Euteleostomi;
            Mammalia; Eutheria; Euarchontoglires; Glires; Rodentia; Myomorpha;
```

```
            Muroidea; Muridae; Murinae; Rattus.
REFERENCE   1  (residues 1 to 638)
  AUTHORS   Vaughan,K.T. and Vallee,R.B.
  TITLE     Cytoplasmic dynein binds dynactin through a direct interaction
            between the intermediate chains and p150Glued
  JOURNAL   J. Cell Biol. 131 (6 Pt 1), 1507-1516 (1995)
   PUBMED   8522607
  REMARK    NUCLEOTIDE SEQUENCE [MRNA] (ISOFORMS 2A; 2B AND 2C), AND
            INTERACTION WITH DCTN1.
REFERENCE   2  (residues 1 to 638)
  AUTHORS   Pfister,K.K., Salata,M.W., Dillman,J.F. 3rd, Torre,E. and Lye,R.J.
  TITLE     Identification and developmental regulation of a neuron-specific
            subunit of cytoplasmic dynein
  JOURNAL   Mol. Biol. Cell 7 (2), 331-343 (1996)
   PUBMED   8688562
  REMARK    IDENTIFICATION IN THE CYTOPLASMIC DYNEIN 1 COMPLEX.
REFERENCE   3  (residues 1 to 638)
  AUTHORS   King,S.M., Barbarese,E., Dillman,J.F. 3rd, Benashski,S.E., Do,K.T.,
            Patel-King,R.S. and Pfister,K.K.
  TITLE     Cytoplasmic dynein contains a family of differentially expressed
            light chains
  JOURNAL   Biochemistry 37 (43), 15033-15041 (1998)
   PUBMED   9790665
  REMARK    IDENTIFICATION IN THE CYTOPLASMIC DYNEIN 1 COMPLEX.
REFERENCE   4  (residues 1 to 638)
  AUTHORS   Tynan,S.H., Gee,M.A. and Vallee,R.B.
  TITLE     Distinct but overlapping sites within the cytoplasmic dynein heavy
            chain for dimerization and for intermediate chain and light
            intermediate chain binding
  JOURNAL   J. Biol. Chem. 275 (42), 32769-32774 (2000)
   PUBMED   10893223
  REMARK    INTERACTION WITH DYNC1H1.
REFERENCE   5  (residues 1 to 638)
  AUTHORS   Vaughan,P.S., Leszyk,J.D. and Vaughan,K.T.
  TITLE     Cytoplasmic dynein intermediate chain phosphorylation regulates
            binding to dynactin
  JOURNAL   J. Biol. Chem. 276 (28), 26171-26179 (2001)
   PUBMED   11340075
  REMARK    FUNCTION, PHOSPHORYLATION AT SER-90, SUBCELLULAR LOCATION,
            INTERACTION WITH DCTN1, MUTAGENESIS OF SER-90, AND IDENTIFICATION
            BY MASS SPECTROMETRY.
REFERENCE   6  (residues 1 to 638)
  AUTHORS   Lo,K.W., Kogoy,J.M., Rasoul,B.A., King,S.M. and Pfister,K.K.
  TITLE     Interaction of the DYNLT (TCTEX1/RP3) light chains and the
            intermediate chains reveals novel intersubunit regulation during
            assembly of the dynein complex
  JOURNAL   J. Biol. Chem. 282 (51), 36871-36878 (2007)
   PUBMED   17965411
  REMARK    INTERACTION WITH DYNLT1 AND DYNLT3.
REFERENCE   7  (residues 1 to 638)
  AUTHORS   Myers,K.R., Lo,K.W., Lye,R.J., Kogoy,J.M., Soura,V., Hafezparast,M.
            and Pfister,K.K.
  TITLE     Intermediate chain subunit as a probe for cytoplasmic dynein
            function: biochemical analyses and live cell imaging in PC12 cells
  JOURNAL   J. Neurosci. Res. 85 (12), 2640-2647 (2007)
   PUBMED   17279546
  REMARK    ALTERNATIVE SPLICING, AND INTERACTION WITH DYNC1H1.
REFERENCE   8  (residues 1 to 638)
  AUTHORS   Lundby,A., Secher,A., Lage,K., Nordsborg,N.B., Dmytriyev,A.,
            Lundby,C. and Olsen,J.V.
  TITLE     Quantitative maps of protein phosphorylation sites across 14
            different rat organs and tissues
  JOURNAL   Nat Commun 3, 876 (2012)
   PUBMED   22673903
  REMARK    PHOSPHORYLATION [LARGE SCALE ANALYSIS] AT SER-51 AND SER-104, AND
            IDENTIFICATION BY MASS SPECTROMETRY [LARGE SCALE ANALYSIS].
            Publication Status: Online-Only
REFERENCE   9  (residues 1 to 638)
  AUTHORS   Merino-Gracia,J., Zamora-Carreras,H., Bruix,M. and
            Rodriguez-Crespo,I.
  TITLE     Molecular Basis for the Protein Recognition Specificity of the
            Dynein Light Chain DYNLT1/Tctex1: CHARACTERIZATION OF THE
            INTERACTION WITH ACTIVIN RECEPTOR IIB
  JOURNAL   J. Biol. Chem. 291 (40), 20962-20975 (2016)
   PUBMED   27502274
  REMARK    INTERACTION WITH DYNLT1.
COMMENT     [FUNCTION] Acts as one of several non-catalytic accessory
            components of the cytoplasmic dynein 1 complex that are thought to
            be involved in linking dynein to cargos and to adapter proteins
            that regulate dynein function. Cytoplasmic dynein 1 acts as a motor
            for the intracellular retrograde motility of vesicles and
            organelles along microtubules. The intermediate chains mediate the
            binding of dynein to dynactin via its 150 kDa component
            (p150-glued) DCNT1. Involved in membrane-transport, such as Golgi
            apparatus, late endosomes and lysosomes.
            {ECO:0000269|PubMed:11340075}.
            [SUBUNIT] Homodimer (By similarity). The cytoplasmic dynein 1
            complex consists of two catalytic heavy chains (HCs) and a number
            of non-catalytic subunits presented by intermediate chains (ICs),
            light intermediate chains (LICs) and light chains (LCs); the
            composition seems to vary in respect to the IC, LIC and LC
            composition. The heavy chain homodimer serves as a scaffold for the
            probable homodimeric assembly of the respective non-catalytic
            subunits. The ICs and LICs bind directly to the HC dimer and the
            LCs assemble on the IC dimer. Isoform 1, isoform 2 and isoform 3
            interact with DYNLT3. Isoform 1, isoform 2 and isoform 3 interact
            with DYNLT1. Interacts (dephosphorylated at Ser-90) with DCTN1.
            Interacts with BICD2 (By similarity). Interacts with SPEF2 (By
            similarity). {ECO:0000250|UniProtKB:O88487,
            ECO:0000269|PubMed:10893223, ECO:0000269|PubMed:11340075,
            ECO:0000269|PubMed:17279546, ECO:0000269|PubMed:17965411,
            ECO:0000269|PubMed:8522607, ECO:0000269|PubMed:8688562,
            ECO:0000269|PubMed:9790665}.
            [SUBCELLULAR LOCATION] Cytoplasm, cytoskeleton
            {ECO:0000269|PubMed:11340075}. Cytoplasm
            {ECO:0000250|UniProtKB:O88487}. Note=Detected in the cytoplasm of
            pachytene spermatocytes. Localizes to the manchette in elongating
            spermatids. {ECO:0000250|UniProtKB:O88487}.
            [ALTERNATIVE PRODUCTS] Event=Alternative splicing; Named
            isoforms=3; Comment=Additional isoforms seem to exist.; Name=2A;
            IsoId=Q62871-1; Sequence=Displayed; Name=2B; IsoId=Q62871-2;
            Sequence=VSP_001339; Name=2C; IsoId=Q62871-3; Sequence=VSP_001339,
            VSP_001340.
```

```
            [TISSUE SPECIFICITY] Skeletal muscle, testis, kidney, brain, heart
            and spleen.
            [PTM] The phosphorylation status of Ser-90 appears to be involved
            in dynactin-dependent target binding.
            {ECO:0000269|PubMed:11340075}.
            [SIMILARITY] Belongs to the dynein intermediate chain family.
            {ECO:0000305}.
FEATURES             Location/Qualifiers
     source          1..638
                     /organism="Rattus norvegicus"
                     /db_xref="taxon:10116"
     gene            1..638
                     /gene="Dync1i2"
                     /gene_synonym="Dnci2"
                     /gene_synonym="Dncic2"
     Protein         1..638
                     /product="Cytoplasmic dynein 1 intermediate chain 2"
                     /note="Cytoplasmic dynein intermediate chain 2; Dynein
                     intermediate chain 2, cytosolic; DH IC-2"
                     /UniProtKB_evidence="Evidence at protein level"
     Region          2..638
                     /region_name="Mature chain"
                     /experiment="experimental evidence, no additional details
                     recorded"
                     /note="Cytoplasmic dynein 1 intermediate chain 2.
                     /FTId=PRO_0000114657."
     Site            2
                     /site_type="acetylation"
                     /experiment="experimental evidence, no additional details
                     recorded"
                     /note="N-acetylserine. {ECO:0000250|UniProtKB:Q13409}."
     Site            51
                     /site_type="phosphorylation"
                     /experiment="experimental evidence, no additional details
                     recorded"
                     /note="Phosphoserine. {ECO:0000244|PubMed:22673903}."
     Region          77..82
                     /region_name="Splicing variant"
                     /experiment="experimental evidence, no additional details
                     recorded"
                     /note="Missing (in isoform 2B and isoform 2C).
                     {ECO:0000303|PubMed:8522607}. /FTId=VSP_001339."
     Site            90
                     /site_type="phosphorylation"
                     /experiment="experimental evidence, no additional details
                     recorded"
                     /note="Phosphoserine. {ECO:0000269|PubMed:11340075}."
     Site            90
                     /site_type="mutagenized"
                     /experiment="experimental evidence, no additional details
                     recorded"
                     /note="S->A: No effect on interaction with DCTN1 (mimicks
                     dephosphorylated form). {ECO:0000269|PubMed:11340075}."
     Site            90
                     /site_type="mutagenized"
                     /experiment="experimental evidence, no additional details
                     recorded"
                     /note="S->D: Impairs interaction with DCTN1 (mimicks
                     phosphorylated form). {ECO:0000269|PubMed:11340075}."
     Site            95
                     /site_type="phosphorylation"
                     /experiment="experimental evidence, no additional details
                     recorded"
                     /note="Phosphothreonine. {ECO:0000250|UniProtKB:Q13409}."
     Site            97
                     /site_type="phosphorylation"
                     /experiment="experimental evidence, no additional details
                     recorded"
                     /note="Phosphoserine. {ECO:0000250|UniProtKB:Q13409}."
     Site            101
                     /site_type="phosphorylation"
                     /experiment="experimental evidence, no additional details
                     recorded"
                     /note="Phosphoserine. {ECO:0000250|UniProtKB:Q13409}."
     Site            104
                     /site_type="phosphorylation"
                     /experiment="experimental evidence, no additional details
                     recorded"
                     /note="Phosphoserine. {ECO:0000244|PubMed:22673903}."
     Region          113..132
                     /region_name="Splicing variant"
                     /experiment="experimental evidence, no additional details
                     recorded"
                     /note="Missing (in isoform 2C).
                     {ECO:0000303|PubMed:8522607}. /FTId=VSP_001340."
     Region          133..165
                     /region_name="Region of interest in the sequence"
                     /experiment="experimental evidence, no additional details
                     recorded"
                     /note="Interaction with DYNLT1.
                     {ECO:0000269|PubMed:27502274}."
     Region          135..163
                     /region_name="Dynein_IC2"
                     /note="Cytoplasmic dynein 1 intermediate chain 2;
                     pfam11540"
                     /db_xref="CDD:288403"
     Region          144..148
                     /region_name="Beta-strand region"
                     /experiment="experimental evidence, no additional details
                     recorded"
                     /note="{ECO:0000244|PDB:2PG1}."
     Region          155..161
                     /region_name="Beta-strand region"
                     /experiment="experimental evidence, no additional details
                     recorded"
                     /note="{ECO:0000244|PDB:2PG1}."
     Region          277..326
                     /region_name="Repetitive region"
                     /experiment="experimental evidence, no additional details
                     recorded"
                     /note="WD 1."
     Region          282..326
                     /region_name="WD40 repeat"
```

```
                     /note="WD40 repeat [structural motif]"
                     /db_xref="CDD:293791"
     Region          329..>599
                     /region_name="WD40"
                     /note="WD40 repeat [General function prediction only];
                     COG2319"
                     /db_xref="CDD:225201"
     Region          330..370
                     /region_name="Repetitive region"
                     /experiment="experimental evidence, no additional details
                     recorded"
                     /note="WD 2."
     Region          339..377
                     /region_name="WD40 repeat"
                     /note="WD40 repeat [structural motif]"
                     /db_xref="CDD:293791"
     Region          379..420
                     /region_name="Repetitive region"
                     /experiment="experimental evidence, no additional details
                     recorded"
                     /note="WD 3."
     Region          380..>605
                     /region_name="WD40"
                     /note="WD40 domain, found in a number of eukaryotic
                     proteins that cover a wide variety of functions including
                     adaptor/regulatory modules in signal transduction,
                     pre-mRNA processing and cytoskeleton assembly; typically
                     contains a GH dipeptide 11-24 residues from...; cl02567"
                     /db_xref="CDD:295369"
     Region          385..426
                     /region_name="WD40 repeat"
                     /note="WD40 repeat [structural motif]"
                     /db_xref="CDD:293791"
     Region          429..469
                     /region_name="Repetitive region"
                     /experiment="experimental evidence, no additional details
                     recorded"
                     /note="WD 4."
     Region          434..481
                     /region_name="WD40 repeat"
                     /note="WD40 repeat [structural motif]"
                     /db_xref="CDD:293791"
     Region          474..519
                     /region_name="Repetitive region"
                     /experiment="experimental evidence, no additional details
                     recorded"
                     /note="WD 5."
     Region          489..521
                     /region_name="WD40 repeat"
                     /note="WD40 repeat [structural motif]"
                     /db_xref="CDD:293791"
     Region          527..565
                     /region_name="WD40 repeat"
                     /note="WD40 repeat [structural motif]"
                     /db_xref="CDD:293791"
     Region          568..607
                     /region_name="Repetitive region"
                     /experiment="experimental evidence, no additional details
                     recorded"
                     /note="WD 6."
     Region          573..599
                     /region_name="WD40 repeat"
                     /note="WD40 repeat [structural motif]"
                     /db_xref="CDD:293791"
```

Mascot: http://www.matrixscience.com/

MASCOT Search Results

User : pbm
E-mail :
Search title : Submitted from MRosenberg Data2009 by Mascot Daemon on SCT-14-65A4
MS data file : G:\PRESTATIONS\Labos académiques\Internationaux\Etats Unis\Martina Rosenberg\Data pour publi M Rosenberg\Identifications Mascot\MR_1650.mgf
Database : NCBIprot 20171205 (139,213,787 sequences; 51,013,024,959 residues)
Taxonomy : Rattus (77,467 sequences)
Timestamp : 25 Jan 2019 at 10:24:45 GMT

Not what you expected? Try **the peptide summary**.

Search parameters

Score distribution

Modification statistics

Legend

Protein Family Summary

Significance threshold p<	0.05	Max. number of families	20
Display non-sig. matches		Dendrograms cut at	0
Preferred taxonomy	All entries		

Sensitivity

Protein families 1–10 (out of 10)

10 per page 1

1	NP_001008825.1	144	keratin, type II cytoskeletal cochleal [Rattus norvegicus]
2	P02651.2	96	RecName: Full=Apolipoprotein A-IV; Short=Apo-AIV; Short=ApoA-IV; AltName: Full=Ap...

		Score	Mass	Matches	Sequences	emPAI
2.1	P02651.2	96	44429	6 (6)	6 (6)	0.79

RecName: Full=Apolipoprotein A-IV; Short=Apo-AIV; Short=ApoA-IV; AltName: Full=Apolipoprotein A4; Flags: Precursor

1 sameset of P02651.2

6 peptide matches (6 non-duplicate, 0 duplicate)

Query	Dupes	Observed	Mr(expt)	Mr(calc)	Delta	M	Score	Expect	Rank	U	Peptide
143		573.3800	1144.7454	1144.6088	0.1367	0	39	0.014	1	U	K.VSTNIDQLQK.N
216		656.8500	1311.6854	1311.7034	-0.0180	0	36	0.0064	1	U	K.NLAPLVEDVQSK.L
249		696.0000	2084.9782	2085.0814	-0.1032	2	39	0.047	1	U	R.ANELKATIDQNLEDLRSR.L
324		789.3600	1576.7054	1577.7685	-1.0631	0	49	0.00082	1	U	K.LGNINTYADDLQNK.L
343		825.8500	1649.6854	1649.8261	-0.1406	0	55	0.0001	1	U	K.TDVTQQLNTLFQDK.L
424		1009.4200	3025.2382	3025.4372	-0.1991	1	40	0.035	1	U	R.MQTTIQDNVENLQSSMVPFANELKEK.F + 2 Oxidation (M)

3	NP_036767.1	92	anionic trypsin-1 precursor [Rattus norvegicus]
4	EDL86877.1	65	type II keratin Kb1 [Rattus norvegicus]
5	1 EDM06018.1	57	keratin complex 1, acidic, gene 19, isoform CRA_b [Rattus norvegicus]
	2 EDM06024.1	43	rCG33578, partial [Rattus norvegicus]

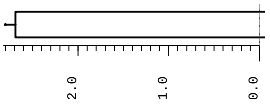

6	XP_006247453.1	37	PREDICTED: keratin, type I cytoskeletal 10 isoform X2 [Rattus norvegicus]
7	EDM04062.1	34	serine/threonine kinase 10, isoform CRA_c [Rattus norvegicus]
8	EDM04573.1	33	rCG63512, partial [Rattus norvegicus]
9	EDL93561.1	32	similar to Gene model 996 (predicted), isoform CRA_a, partial [Rattus norvegicus]
10	EDM18662.1	23	transcription factor AP-2 beta (predicted) [Rattus norvegicus]

10 per page 1

Not what you expected? Try **the peptide summary**.

Mascot: http://www.matrixscience.com/

MATRIX SCIENCE MASCOT Search Results

Protein View: P02651.2

RecName: Full=Apolipoprotein A-IV; Short=Apo-AIV; Short=ApoA-IV; AltName: Full=Apolipoprotein A4; Flags: Precursor

**Database:**	NCBIprot
**Score:**	96
**Monoisotopic mass (M_r):**	44429
**Calculated pI:**	5.12
**Taxonomy:**	Rattus norvegicus

This protein sequence matches the following other entries:

- AAA40747.1 from Rattus norvegicus
- AAA40748.1 from Rattus norvegicus
- AAH91159.1 from Rattus norvegicus
- EDL95397.1 from Rattus norvegicus

Sequence similarity is available as an NCBI BLAST search of P02651.2 against nr.

Search parameters

**MS data file:**	G:\PRESTATIONS\Labos académiques\Internationaux\Etats Unis\Martina Rosenberg\Data pour publi M Rosenberg\Identifications Mascot\MR_1650.mgf
**Enzyme:**	Trypsin: cuts C-term side of KR unless next residue is P.
**Variable modifications:**	Carbamidomethyl (C), Oxidation (M)

Protein sequence coverage: 24%

Matched peptides shown in ***bold red***.

```
  1 MFLKAVVLTV ALVAITGTQA EVTSDQVANV MWDYFTQLSN NAKEAVEQLQ
 51 KTDVTQQLNT LFQDKLGNIN TYADDLQNKL VPFAVQLSGH LTKETERVRE
101 EIQKELEDLR ANMMPHANKV SQMFGDNVQK LQEHLRPYAT DLQAQINAQT
151 QDMKRQLTPY IQRMQTTIQD NVENLQSSMV PFANELKEKF NQNMEGLKGQ
201 LTPRANELKA TIDQNLEDLR SRLAPLAEGV QEKLNHQMEG LAFQMKKNAE
251 ELQTKVSTNI DQLQKNLAPL VEDVQSKLKG NTEGLQKSLE DLNKQLDQQV
301 EVFRRAVEPL GDKFNMALVQ QMEKFRQQLG SDSGDVESHL SFLEKNLREK
351 VSSFMSTLQK KGSPDQPLAL PLPEQVQEQV QEQVQPKPLE S
```

Unformatted sequence string: 391 residues (for pasting into other applications).

Sort by ◉ residue number ○ increasing mass ○ decreasing mass
Show ◉ matched peptides only ○ predicted peptides also

Query	Start – End	Observed	Mr(expt)	Mr(calc)	Delta	M	Score	Expect	Rank	U	Peptide
343	52 – 65	825.8500	1649.6854	1649.8261	-0.1406	0	55	0.0001	1	U	K.TDVTQQLNTLFQDK.L
324	66 – 79	789.3600	1576.7054	1577.7685	-1.0631	0	49	0.00082	1	U	K.LGNINTYADDLQNK.L
424	164 – 189	1009.4200	3025.2382	3025.4372	-0.1991	1	40	0.035	1	U	R.MQTTIQDNVENLQSSMVPFANELKEK.F + 2 Oxidation (M)
249	205 – 222	696.0000	2084.9782	2085.0814	-0.1032	2	39	0.047	1	U	R.ANELKATIDQNLEDLRSR.L
143	256 – 265	573.3800	1144.7454	1144.6088	0.1367	0	39	0.014	1	U	K.VSTNIDQLQK.N
216	266 – 277	656.8500	1311.6854	1311.7034	-0.0180	0	36	0.0064	1	U	K.NLAPLVEDVQSK.L

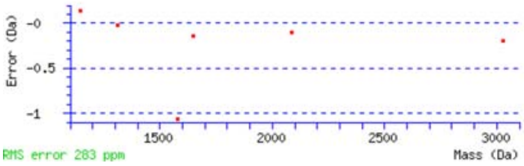

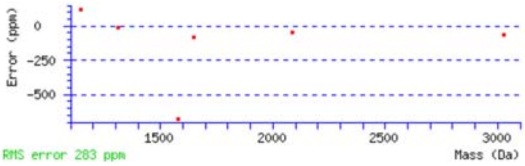


```
LOCUS       APOA4_RAT                391 aa            linear   ROD 16-JAN-2019
DEFINITION  RecName: Full=Apolipoprotein A-IV; Short=Apo-AIV; Short=ApoA-IV;
            AltName: Full=Apolipoprotein A4; Flags: Precursor.
ACCESSION   P02651
VERSION     P02651.2
DBSOURCE    UniProtKB: locus APOA4_RAT, accession P02651;
            class: standard.
            extra accessions:Q5BK92
            created: Jul 21, 1986.
            sequence updated: Jul 1, 1989.
            annotation updated: Jan 16, 2019.
            xrefs: M00002.1, AAA85909.1, J02588.1, AAA40747.1, M13508.1,
            AAA40748.1, BC091159.1, AAH91159.1, LPRTA4, NP_036869.1
            xrefs (non-sequence databases): UniGene:Rn.15739,
            ProteinModelPortal:P02651, SMR:P02651, IntAct:P02651,
            iPTMnet:P02651, PhosphoSitePlus:P02651, PRIDE:P02651, GeneID:25080,
            KEGG:rno:25080, UCSC:RGD:2132, CTD:337, RGD:2132,
            HOVERGEN:HBG105707, InParanoid:P02651, KO:K08760,
            OrthoDB:1299087at2759, PhylomeDB:P02651, PRO:PR:P02651,
            Proteomes:UP000002494, GO:0042627, GO:0034364, GO:0045202,
            GO:0015485, GO:0017127, GO:0031210, GO:0060228, GO:0005543,
            GO:0006695, GO:0033344, GO:0042632, GO:0034380, GO:0006869,
            GO:0042157, GO:0014012, GO:0046470, GO:0033700, GO:0010873,
            GO:0045723, GO:0046889, GO:0051006, GO:0010898, GO:0032374,
            GO:0030300, GO:0032094, GO:0034014, GO:0043691, GO:0019433,
            GO:0070328, GO:0034372, InterPro:IPR000074, Pfam:PF01442
KEYWORDS    Chylomicron; Complete proteome; HDL; Lipid transport;
            Phosphoprotein; Reference proteome; Repeat; Secreted; Signal;
            Transport.
SOURCE      Rattus norvegicus (Norway rat)
  ORGANISM  Rattus norvegicus
            Eukaryota; Metazoa; Chordata; Craniata; Vertebrata; Euteleostomi;
            Mammalia; Eutheria; Euarchontoglires; Glires; Rodentia; Myomorpha;
            Muroidea; Muridae; Murinae; Rattus.
```

```
REFERENCE   1  (residues 1 to 391)
  AUTHORS   Boguski,M.S., Birkenmeier,E.H., Elshourbagy,N.A., Taylor,J.M. and
            Gordon,J.I.
  TITLE     Evolution of the apolipoproteins. Structure of the rat apo-A-IV
            gene and its relationship to the human genes for apo-A-I, C-III,
            and E
  JOURNAL   J. Biol. Chem. 261 (14), 6398-6407 (1986)
   PUBMED   3009456
  REMARK    NUCLEOTIDE SEQUENCE [GENOMIC DNA].
REFERENCE   2  (residues 1 to 391)
  AUTHORS   Boguski,M.S., Elshourbagy,N., Taylor,J.M. and Gordon,J.I.
  TITLE     Rat apolipoprotein A-IV contains 13 tandem repetitions of a
            22-amino acid segment with amphipathic helical potential
  JOURNAL   Proc. Natl. Acad. Sci. U.S.A. 81 (16), 5021-5025 (1984)
   PUBMED   6591177
  REMARK    NUCLEOTIDE SEQUENCE [MRNA].
REFERENCE   3  (residues 1 to 391)
  AUTHORS   Haddad,I.A., Ordovas,J.M., Fitzpatrick,T. and Karathanasis,S.K.
  TITLE     Linkage, evolution, and expression of the rat apolipoprotein A-I,
            C-III, and A-IV genes
  JOURNAL   J. Biol. Chem. 261 (28), 13268-13277 (1986)
   PUBMED   3020028
  REMARK    NUCLEOTIDE SEQUENCE [GENOMIC DNA].
REFERENCE   4  (residues 1 to 391)
  AUTHORS   Gerhard,D.S., Wagner,L., Feingold,E.A., Shenmen,C.M., Grouse,L.H.,
            Schuler,G., Klein,S.L., Old,S., Rasooly,R., Good,P., Guyer,M.,
            Peck,A.M., Derge,J.G., Lipman,D., Collins,F.S., Jang,W., Sherry,S.,
            Feolo,M., Misquitta,L., Lee,E., Rotmistrovsky,K., Greenhut,S.F.,
            Schaefer,C.F., Buetow,K., Bonner,T.I., Haussler,D., Kent,J.,
            Kiekhaus,M., Furey,T., Brent,M., Prange,C., Schreiber,K.,
            Shapiro,N., Bhat,N.K., Hopkins,R.F., Hsie,F., Driscoll,T.,
            Soares,M.B., Casavant,T.L., Scheetz,T.E., Brown-stein,M.J.,
            Usdin,T.B., Toshiyuki,S., Carninci,P., Piao,Y., Dudekula,D.B.,
            Ko,M.S., Kawakami,K., Suzuki,Y., Sugano,S., Gruber,C.E.,
            Smith,M.R., Simmons,B., Moore,T., Waterman,R., Johnson,S.L.,
            Ruan,Y., Wei,C.L., Mathavan,S., Gunaratne,P.H., Wu,J., Garcia,A.M.,
            Hulyk,S.W., Fuh,E., Yuan,Y., Sneed,A., Kowis,C., Hodgson,A.,
            Muzny,D.M., McPherson,J., Gibbs,R.A., Fahey,J., Helton,E.,
            Ketteman,M., Madan,A., Rodrigues,S., Sanchez,A., Whiting,M.,
            Madari,A., Young,A.C., Wetherby,K.D., Granite,S.J., Kwong,P.N.,
            Brinkley,C.P., Pearson,R.L., Bouffard,G.G., Blakesly,R.W.,
            Green,E.D., Dickson,M.C., Rodriguez,A.C., Grimwood,J., Schmutz,J.,
            Myers,R.M., Butterfield,Y.S., Griffith,M., Griffith,O.L.,
            Krzywinski,M.I., Liao,N., Morin,R., Palmquist,D., Petrescu,A.S.,
            Skalska,U., Smailus,D.E., Stott,J.M., Schnerch,A., Schein,J.E.,
            Jones,S.J., Holt,R.A., Baross,A., Marra,M.A., Clifton,S.,
            Makowski,K.A., Bosak,S. and Malek,J.
  CONSRTM   MGC Project Team
  TITLE     The status, quality, and expansion of the NIH full-length cDNA
            project: the Mammalian Gene Collection (MGC)
  JOURNAL   Genome Res. 14 (10B), 2121-2127 (2004)
   PUBMED   15489334
  REMARK    NUCLEOTIDE SEQUENCE [LARGE SCALE MRNA].;
            TISSUE=Liver
            Erratum:[Genome Res. 2006 Jun;16(6):804. Morrin, Ryan [corrected to
            Morin, Ryan]]
REFERENCE   5  (residues 1 to 391)
  AUTHORS   Lundby,A., Secher,A., Lage,K., Nordsborg,N.B., Dmytriyev,A.,
            Lundby,C. and Olsen,J.V.
  TITLE     Quantitative maps of protein phosphorylation sites across 14
            different rat organs and tissues
  JOURNAL   Nat Commun 3, 876 (2012)
   PUBMED   22673903
  REMARK    PHOSPHORYLATION [LARGE SCALE ANALYSIS] AT SER-333, AND
            IDENTIFICATION BY MASS SPECTROMETRY [LARGE SCALE ANALYSIS].
            Publication Status: Online-Only
COMMENT     On or before May 9, 2007 this sequence version replaced
            gi:81882616, gi:71798.
            [FUNCTION] May have a role in chylomicrons and VLDL secretion and
            catabolism. Required for efficient activation of lipoprotein lipase
            by ApoC-II; potent activator of LCAT. Apoa-IV is a major component
            of HDL and chylomicrons.
            [SUBUNIT] Homodimer. {ECO:0000250|UniProtKB:P06727}.
            [SUBCELLULAR LOCATION] Secreted.
            [TISSUE SPECIFICITY] Secreted in plasma.
            [DOMAIN] Nine of the thirteen 22-amino acid tandem repeats (each
            22-mer is actually a tandem array of two, A and B, related 11-mers)
            occurring in this sequence are predicted to be highly
            alpha-helical, and many of these helices are amphipathic. They may
            therefore serve as lipid-binding domains with lecithin:cholesterol
            acyltransferase (LCAT) activating abilities.
            [SIMILARITY] Belongs to the apolipoprotein A1/A4/E family.
            {ECO:0000305}.
FEATURES             Location/Qualifiers
     source          1..391
                     /organism="Rattus norvegicus"
                     /db_xref="taxon:10116"
     gene            1..391
                     /gene="Apoa4"
     Protein         1..391
                     /product="Apolipoprotein A-IV"
                     /note="Apo-AIV; ApoA-IV; Apolipoprotein A4"
                     /UniProtKB_evidence="Evidence at protein level"
     Region          1..20
                     /region_name="Signal"
                     /experiment="experimental evidence, no additional details
                     recorded"
     Region          21..391
                     /region_name="Mature chain"
                     /experiment="experimental evidence, no additional details
                     recorded"
                     /note="Apolipoprotein A-IV. /FTId=PRO_0000001980."
     Region          33..330
                     /region_name="Region of interest in the sequence"
                     /experiment="experimental evidence, no additional details
                     recorded"
                     /note="13 X 22 AA approximate tandem repeats."
     Region          33..54
                     /region_name="Repetitive region"
                     /experiment="experimental evidence, no additional details
                     recorded"
                     /note="1."
     Region          60..81
```

```
                /region_name="Repetitive region"
                /experiment="experimental evidence, no additional details
                recorded"
                /note="2."
Region          66..235
                /region_name="Apolipoprotein"
                /note="Apolipoprotein A1/A4/E domain; pfam01442"
                /db_xref="CDD:279749"
Region          82..103
                /region_name="Repetitive region"
                /experiment="experimental evidence, no additional details
                recorded"
                /note="3."
Region          115..136
                /region_name="Repetitive region"
                /experiment="experimental evidence, no additional details
                recorded"
                /note="4."
Region          137..158
                /region_name="Repetitive region"
                /experiment="experimental evidence, no additional details
                recorded"
                /note="5."
Region          159..180
                /region_name="Repetitive region"
                /experiment="experimental evidence, no additional details
                recorded"
                /note="6."
Region          181..202
                /region_name="Repetitive region"
                /experiment="experimental evidence, no additional details
                recorded"
                /note="7."
Region          184..329
                /region_name="DUF4795"
                /note="Domain of unknown function (DUF4795); pfam16043"
                /db_xref="CDD:292662"
Region          203..224
                /region_name="Repetitive region"
                /experiment="experimental evidence, no additional details
                recorded"
                /note="8."
Region          225..246
                /region_name="Repetitive region"
                /experiment="experimental evidence, no additional details
                recorded"
                /note="9."
Region          247..268
                /region_name="Repetitive region"
                /experiment="experimental evidence, no additional details
                recorded"
                /note="10."
Region          253
                /region_name="Variant"
                /experiment="experimental evidence, no additional details
                recorded"
                /note="Q -> H."
Region          255..>361
                /region_name="Apolipoprotein"
                /note="Apolipoprotein A1/A4/E domain; pfam01442"
                /db_xref="CDD:279749"
Region          269..286
                /region_name="Repetitive region"
                /experiment="experimental evidence, no additional details
                recorded"
                /note="11."
Region          287..308
                /region_name="Repetitive region"
                /experiment="experimental evidence, no additional details
                recorded"
                /note="12."
Region          309..330
                /region_name="Repetitive region"
                /experiment="experimental evidence, no additional details
                recorded"
                /note="13."
Site            333
                /site_type="phosphorylation"
                /experiment="experimental evidence, no additional details
                recorded"
                /note="Phosphoserine. {ECO:0000244|PubMed:22673903}."
Region          374..385
                /region_name="Compositionally biased region"
                /experiment="experimental evidence, no additional details
                recorded"
                /note="Gln/Glu-rich."
```

Mascot: http://www.matrixscience.com/

MATRIX SCIENCE MASCOT Search Results

User : pbm
E-mail :
Search title : Submitted from MRosenberg Data2009 by Mascot Daemon on SCT-14-65A4
MS data file : G:\PRESTATIONS\Labos académiques\Internationaux\Etats Unis\Martina Rosenberg\Data pour publi M Rosenberg\Identifications Mascot\MR_1409.mgf
Database : NCBIprot 20171205 (139,213,787 sequences; 51,013,024,959 residues)
Taxonomy : Rattus (77,467 sequences)
Timestamp : 25 Jan 2019 at 10:21:39 GMT

Not what you expected? Try **the peptide summary**.

Search parameters

Score distribution

Modification statistics

Legend

Protein Family Summary

Significance threshold p<	0.05	Max. number of families	20
Display non-sig. matches	☐	Dendrograms cut at	0
Preferred taxonomy	All entries		

Sensitivity

Protein families 1–9 (out of 9)

10 per page 1

1

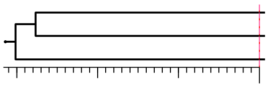

1	XP_003754401.1	355	PREDICTED: keratin, type II cytoskeletal 7 [Rattus norvegicus]
3	NP_001008802.2	91	keratin, type II cytoskeletal 1 [Rattus norvegicus]
2	EDL86881.1	93	rCG50775 [Rattus norvegicus]

Threshold (**0**): 0

		Score	Mass	Matches	Sequences	emPAI
1.1	XP_003754401.1	355	50775	13 (13)	11 (11)	1.55
	PREDICTED: keratin, type II cytoskeletal 7 [Rattus norvegicus]					
1.2	EDL86881.1	93	56740	2 (2)	2 (2)	0.16
	rCG50775 [Rattus norvegicus]					
	**13 samesets of EDL86881.1**					
	Q6P6Q2.1	93	61788	2 (2)	2 (2)	
	RecName: Full=Keratin, type II cytoskeletal 5; AltName: Full=Cytokeratin-5; Short=CK-5; AltName: Full=Keratin-5; Short=K5; AltName: Full=Type-II keratin Kb5					
	NP_899162.1	93	61889	2 (2)	2 (2)	
	keratin, type II cytoskeletal 5 [Rattus norvegicus]					
	NP_001008825.1	93	57574	2 (2)	2 (2)	
	keratin, type II cytoskeletal cochleal [Rattus norvegicus]					
	NP_001094477.1	93	59213	2 (2)	2 (2)	
	keratin, type II cytoskeletal 6A [Rattus norvegicus]					
	XP_002727023.1	93	59390	2 (2)	2 (2)	
	PREDICTED: keratin, type II cytoskeletal 6A [Rattus norvegicus]					
	XP_003750458.1	93	57274	2 (2)	2 (2)	
	PREDICTED: LOW QUALITY PROTEIN: keratin, type II cytoskeletal 79 isoform X1 [Rattus norvegicus]					
	XP_006242387.1	93	58991	2 (2)	2 (2)	
	PREDICTED: keratin, type II cytoskeletal 75 isoform X1 [Rattus norvegicus]					
	XP_006242409.1	93	58060	2 (2)	2 (2)	
	PREDICTED: keratin, type II cytoskeletal cochleal isoform X1 [Rattus norvegicus]					
	XP_008774828.1	93	59343	2 (2)	2 (2)	
	PREDICTED: keratin, type II cytoskeletal 6A-like isoform X1 [Rattus norvegicus]					
	XP_008774829.1	93	60145	2 (2)	2 (2)	
	PREDICTED: keratin, type II cytoskeletal 6A-like isoform X2 [Rattus norvegicus]					
	XP_008774848.1	93	59315	2 (2)	2 (2)	
	PREDICTED: LOW QUALITY PROTEIN: keratin, type II cytoskeletal 6A-like [Rattus norvegicus]					
	XP_008774849.2	93	54499	2 (2)	2 (2)	
	PREDICTED: keratin, type II cytoskeletal 79 isoform X2 [Rattus norvegicus]					
	XP_017459006.1	93	60272	2 (2)	2 (2)	
	PREDICTED: keratin, type II cytoskeletal 6A-like isoform X4 [Rattus norvegicus]					
1.3	NP_001008802.2	91	64717	3 (3)	3 (3)	0.22
	keratin, type II cytoskeletal 1 [Rattus norvegicus]					

16 peptide matches (15 non-duplicate, 1 duplicate)

Query	Dupes	Observed	Mr(expt)	Mr(calc)	Delta	M	Score	Expect	Rank	U	1	2	3	Peptide
81		455.0500	1362.1282	1361.7554	0.3727	1	39	0.028	1	U	■			R.TAAENEFVLLKK.D
113		508.8500	1015.6854	1015.5550	0.1305	0	42	0.019	1	U	■			K.LQAEIDTVK.N
134		541.7800	1081.5454	1081.5920	-0.0466	1	70	0.00018	1		■	■	■	K.FASFIDKVR.F
139		546.7300	1091.4454	1091.5611	-0.1157	0	39	0.021	1	U	■			K.FETLQAQAGK.H
144	1	552.8400	1103.6654	1103.5724	0.0931	0	63	0.00018	1	U	■			R.SAYGGPVGAGIR.E
148		559.4700	1116.9254	1116.6291	0.2963	0	61	0.00051	1	U	■			R.IFEAQIAGLR.Q
196		630.1500	1258.2854	1257.6816	0.6038	0	86	7.6e-07	1	U	■			R.SLDLDGIIADVK.A
209		651.9200	1301.8254	1301.7078	0.1176	0	60	4.9e-05	1	U		■		R.SLDLDSIIAEVK.A
221		664.7800	1327.5454	1327.7347	-0.1892	1	56	8.4e-05	1	U	■			R.AKLAEVEEALQK.A
233		681.8400	1361.6654	1361.7554	-0.0900	1	78	2.3e-06	1	U	■			R.TAAENEFVLLKK.D

Query	Dupes	Observed	Mr(expt)	Mr(calc)	Delta	M	Score	Expect	Rank	U	1	2	3	Peptide
243		707.8400	1413.6654	1413.7576	-0.0921	1	85	7.2e-06	1	U	■			K.LQAEIDTVKNQR.A
253		719.9300	2156.7682	2156.0723	0.6959	2	57	0.00013	1	U	■			K.FETLQAQAGKHGDDLRNTR.N
262		738.3600	1474.7054	1474.7780	-0.0725	0	58	0.0029	1	U			■	R.FLEQQNQVLQTK.W
291		786.6800	2357.0182	2357.2074	-0.1892	2	62	8.6e-05	1	U	■			R.AKLESSIAEAEEQGELALKDAR.A
316		819.8800	1637.7454	1638.8729	-1.1275	2	40	0.0018	1	U			■	K.SLNDKFASFIDKVR.F

6 subsets and intersections (20 subset proteins in total)

2	NP_071976.1	78	desmin [Rattus norvegicus]
3	NP_001008751.1	62	keratin, type I cytoskeletal 14 [Rattus norvegicus]
4	XP_006247453.1	53	PREDICTED: keratin, type I cytoskeletal 10 isoform X2 [Rattus norvegicus]
5	NP_036767.1	45	anionic trypsin-1 precursor [Rattus norvegicus]
6	EDL86466.1	35	rCG45115 [Rattus norvegicus]
7	NP_001014791.1	32	RING finger protein 113A [Rattus norvegicus]
8	XP_008773732.1	29	PREDICTED: LOW QUALITY PROTEIN: C-1-tetrahydrofolate synthase, cytoplasmic-like [R...
9	EDL96990.1	29	dynein, axonemal, heavy polypeptide 8, partial [Rattus norvegicus]

10 per page 1

Not what you expected? Try the peptide summary.

Mascot: http://www.matrixscience.com/

MATRIX SCIENCE MASCOT Search Results

Protein View: XP_003754401.1

PREDICTED: keratin, type II cytoskeletal 7 [Rattus norvegicus]

**Database:**	NCBIprot
**Score:**	355
**Monoisotopic mass (M_r):**	50775
**Calculated pI:**	5.67
**Taxonomy:**	Rattus norvegicus

This protein sequence matches the following other entries:

- XP_003750455.1 from Rattus norvegicus
- XP_017459005.1 from Rattus norvegicus
- XP_017450823.1 from Rattus norvegicus
- EDL86895.1 from Rattus norvegicus

Sequence similarity is available as an NCBI BLAST search of XP_003754401.1 against nr.

Search parameters

**MS data file:**	G:\PRESTATIONS\Labos académiques\Internationaux\Etats Unis\Martina Rosenberg\Data pour publi M Rosenberg\Identifications Mascot\MR_1409.mgf
**Enzyme:**	Trypsin: cuts C-term side of KR unless next residue is P.
**Variable modifications:**	Carbamidomethyl (C), Oxidation (M)

Protein sequence coverage: 26%

Matched peptides shown in ***bold red***.

1 MSIHFSSRST AYPGRGAQVR LSSGRAGFGS RSLYGLGTSR PRVAVR**SAYG**
51 **GPVGAGIR**EI TINQNLLAPL SVDIDPTIQQ VRQEEREQIK TLNNK**FASFI**
101 **DKVR**FLEQQN KMLETKWALL QDQKSAKSSQ LPR**IFEAQIA** **GLR**QQLEALQ
151 LDGGRLEVEL RSMQDVVEDF KNKYEEEINR R**TAAENEFVL** **LKK**DVDAAYT
201 NKVELEAKAD SLQDKINFLK TLHETELAEL QSQISDTCVV LSMDNSR**SLD**
251 **LDGIIADVK**A QYEEMANHSQ AEAEAWYQTK **FETLQAQAGK** **HGDDLRNTR**N
301 EIAEMNRSIQ K**LQAEIDTVK** **NQRAKLESSI** **AEAEEQGELA** **LKDARAKLAE**
351 **VEEALQK**AKQ DLARLLREYQ ELMNVKLGLD IEIATYRKLL EGEESRLSGD
401 GMEPVNISVV NSTGANGSRL TIGGTMGSNA LSFSGGPGVL RTYSIKTTST
451 ARRGTHH

Unformatted sequence string: 457 residues (for pasting into other applications).

Sort by ◉ residue number ○ increasing mass ○ decreasing mass
Show ◉ matched peptides only ○ predicted peptides also

Query	Start – End	Observed	Mr(expt)	Mr(calc)	Delta	M	Score	Expect	Rank	U	Peptide
143	47 – 58	552.8400	1103.6654	1103.5724	0.0931	0	60	0.0003	1	U	R.SAYGGPVGAGIR.E
144	47 – 58	552.8400	1103.6654	1103.5724	0.0931	0	63	0.00018	1	U	R.SAYGGPVGAGIR.E
134	96 – 104	541.7800	1081.5454	1081.5920	-0.0466	1	70	0.00018	1		K.FASFIDKVR.F
148	134 – 143	559.4700	1116.9254	1116.6291	0.2963	0	61	0.00051	1	U	R.IFEAQIAGLR.Q
233	182 – 193	681.8400	1361.6654	1361.7554	-0.0900	1	78	2.3e-06	1	U	R.TAAENEFVLLKK.D
81	182 – 193	455.0500	1362.1282	1361.7554	0.3727	1	39	0.028	1	U	R.TAAENEFVLLKK.D
196	248 – 259	630.1500	1258.2854	1257.6816	0.6038	0	86	7.6e-07	1	U	R.SLDLDGIIADVK.A
139	281 – 290	546.7300	1091.4454	1091.5611	-0.1157	0	39	0.021	1	U	K.FETLQAQAGK.H
253	281 – 299	719.9300	2156.7682	2156.0723	0.6959	2	57	0.00013	1	U	K.FETLQAQAGKHGDDLRNTR.N
113	312 – 320	508.8500	1015.6854	1015.5550	0.1305	0	42	0.019	1	U	K.LQAEIDTVK.N
243	312 – 323	707.8400	1413.6654	1413.7576	-0.0921	1	85	7.2e-06	1	U	K.LQAEIDTVKNQR.A
291	324 – 345	786.6800	2357.0182	2357.2074	-0.1892	2	62	8.6e-05	1	U	R.AKLESSIAEAEEQGELALKDAR.A
221	346 – 357	664.7800	1327.5454	1327.7347	-0.1892	1	56	8.4e-05	1	U	R.AKLAEVEEALQK.A

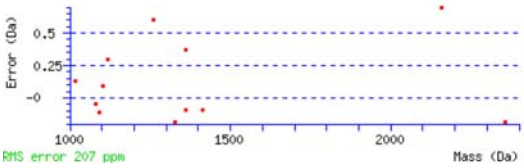

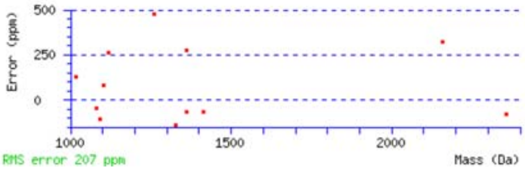


```
LOCUS       XP_003754401             457 aa            linear   ROD 26-JUL-2016
DEFINITION  PREDICTED: keratin, type II cytoskeletal 7 [Rattus norvegicus].
ACCESSION   XP_003754401
VERSION     XP_003754401.1
DBLINK      BioProject: PRJNA16219
DBSOURCE    REFSEQ: accession XM_003754353.4
KEYWORDS    RefSeq.
SOURCE      Rattus norvegicus (Norway rat)
  ORGANISM  Rattus norvegicus
            Eukaryota; Metazoa; Chordata; Craniata; Vertebrata; Euteleostomi;
            Mammalia; Eutheria; Euarchontoglires; Glires; Rodentia; Myomorpha;
            Muroidea; Muridae; Murinae; Rattus.
COMMENT     MODEL REFSEQ:  This record is predicted by automated computational
            analysis. This record is derived from a genomic sequence
            (AC_000075.1) annotated using gene prediction method: Gnomon,
            supported by EST evidence.
            Also see:
                Documentation of NCBI's Annotation Process

            ##Genome-Annotation-Data-START##
            Annotation Provider         :: NCBI
            Annotation Status           :: Full annotation
            Annotation Version          :: Rattus norvegicus Annotation Release
                                           106
```

```
            Annotation Pipeline         :: NCBI eukaryotic genome annotation
                                           pipeline
            Annotation Software Version :: 7.1
            Annotation Method           :: Best-placed RefSeq; Gnomon
            Features Annotated          :: Gene; mRNA; CDS; ncRNA
            ##Genome-Annotation-Data-END##
            COMPLETENESS: full length.
FEATURES             Location/Qualifiers
     source          1..457
                     /organism="Rattus norvegicus"
                     /strain="BN; Sprague-Dawley"
                     /db_xref="taxon:10116"
                     /chromosome="7"
     Protein         1..457
                     /product="keratin, type II cytoskeletal 7"
                     /calculated_mol_wt=50675
     Region          5..81
                     /region_name="Keratin_2_head"
                     /note="Keratin type II head; pfam16208"
                     /db_xref="CDD:292825"
     Region          84..396
                     /region_name="Filament"
                     /note="Intermediate filament protein; pfam00038"
                     /db_xref="CDD:278467"
     Region          <104..220
                     /region_name="GBP_C"
                     /note="Guanylate-binding protein, C-terminal domain;
                     cl20817"
                     /db_xref="CDD:303769"
     Region          189..200
                     /region_name="coiled coil"
                     /note="coiled coil [structural motif]"
                     /db_xref="CDD:293879"
     Region          209..220
                     /region_name="coiled coil"
                     /note="coiled coil [structural motif]"
                     /db_xref="CDD:293879"
     CDS             1..457
                     /gene="Krt7"
                     /coded_by="XM_003754353.4:565..1938"
                     /db_xref="GeneID:300242"
                     /db_xref="RGD:1310865"
```

Mascot: http://www.matrixscience.com/

MATRIX SCIENCE MASCOT Search Results

User : pbm
E-mail :
Search title : Submitted from MRosenberg Data2009 by Mascot Daemon on SCT-14-65A4
MS data file : G:\PRESTATIONS\Labos académiques\Internationaux\Etats Unis\Martina Rosenberg\Data pour publi M Rosenberg\Identifications Mascot\MR_1069B.mgf
Database : NCBIprot 20171205 (139,213,787 sequences; 51,013,024,959 residues)
Taxonomy : Rattus (77,467 sequences)
Timestamp : 25 Jan 2019 at 10:15:33 GMT

Not what you expected? Try **the peptide summary**.

Search parameters
Score distribution
Modification statistics
Legend

Protein Family Summary

Significance threshold p<	0.05	Max. number of families	20
Display non-sig. matches	☐	Dendrograms cut at	0
Preferred taxonomy	All entries		

Sensitivity

Protein families 1–5 (out of 5)

10 per page 1

1 **pir||S31716** 125 dnaK-type molecular chaperone hsp72-ps1 - rat

		Score	Mass	Matches	Sequences	emPAI
1.1	pir||S31716	125	70884	6 (6)	5 (5)	0.36

dnaK-type molecular chaperone hsp72-ps1 - rat

3 samesets of pir||S31716

	Score	Mass	Matches	Sequences
NP_077327.1	125	70827	6 (6)	5 (5)
XP_008762842.1	125	70998	6 (6)	5 (5)
XP_008774440.1	125	71032	6 (6)	5 (5)

NP_077327.1: heat shock cognate 71 kDa protein [Rattus norvegicus]
XP_008762842.1: PREDICTED: LOW QUALITY PROTEIN: heat shock cognate 71 kDa protein-like [Rattus norvegicus]
XP_008774440.1: PREDICTED: LOW QUALITY PROTEIN: heat shock cognate 71 kDa protein-like [Rattus norvegicus]

6 peptide matches (5 non-duplicate, 1 duplicate)

Query	Dupes	Observed	Mr(expt)	Mr(calc)	Delta	M	Score	Expect	Rank	U	Peptide
198	1	600.3600	1198.7054	1198.6670	0.0385	0	49	0.0044	1	U	K.DAGTIAGLNVLR.I
232		659.6000	1317.1854	1318.5863	-1.4009	0	52	0.00024	1	U	K.NSLESYAFNMK.A + Oxidation (M)
343		833.7900	1665.5654	1664.7828	0.7826	0	40	0.0018	1	U	K.NQVAMNPTNTVFDAK.R + Oxidation (M)
400		947.4500	2839.3282	2839.5443	-0.2161	1	42	0.0056	1	U	K.DAGTIAGLNVLRIINEPTAAAIAYGLDK.K
425		990.2700	2967.7882	2967.6393	0.1489	2	52	0.00012	1	U	K.DAGTIAGLNVLRIINEPTAAAIAYGLDKK.V

2 subsets and intersections (2 subset proteins in total)

2	XP_008763925.1	58	PREDICTED: keratin, type II cytoskeletal 73 isoform X1 [Rattus norvegicus]
3	Q6P6Q2.1	57	RecName: Full=Keratin, type II cytoskeletal 5; AltName: Full=Cytokeratin-5; Short=CK-5; AltName: Full=Keratin-5; Short=K5; AltName...
4	NP_036767.1	54	anionic trypsin-1 precursor [Rattus norvegicus]
5	CAB94693.1	36	immunoglobulin heavy chain variable region, partial [Rattus norvegicus]

10 per page 1

Not what you expected? Try **the peptide summary**.

Mascot: http://www.matrixscience.com/

MATRIX SCIENCE MASCOT Search Results

Protein View: pir||S31716

dnaK-type molecular chaperone hsp72-ps1 - rat

**Database:**	NCBIprot
**Score:**	125
**Monoisotopic mass (M_r):**	70884
**Calculated pI:**	5.43
**Taxonomy:**	

This protein sequence matches the following other entries:

- CAA49670.1 from **Rattus norvegicus**

Sequence similarity is available as **an NCBI BLAST search of pir||S31716 against nr**.

Search parameters

**MS data file:**	G:\PRESTATIONS\Labos académiques\Internationaux\Etats Unis\Martina Rosenberg\Data pour publi M Rosenberg\Identifications Mascot\MR_1069B.mgf
**Enzyme:**	Trypsin: cuts C-term side of KR unless next residue is P.
**Variable modifications:**	**Carbamidomethyl (C)**, **Oxidation (M)**

Protein sequence coverage: 8%

Matched peptides shown in ***bold red***.

```
  1 MSKGPAVGID LGTTYSCVGV FQHGKVEIIA NDQGNRTTPS YVAFTDTERL
 51 IGDAAKNQVA MNPTNTVFDA KRLIGRRFDD AVVQSDMKHW PFMVVNDAGR
101 PKVQVEYKGE TKSFYPEEVS SMVLTKMKEI AEAYLGKTVT NAVVTVPAYF
151 NDSQRQAAKD AGTIAGLNVL RIINEPTAAA IAYGLDKKVR AERNVLIFDL
201 GGGTFDVSIL TTEDGIFEVK STAGDTHLGG EDFDNRMVNH FIAEFKRKHK
251 KDISENKRAV RRLRTACERA KRTLSSSTQA SIEIDSLYEG IDFYTSITRA
301 RFEELNADLF RGTLDPVEKA LRDAKLDKSQ IHDIVLVGGS TRIPKIQKLL
351 QDFFNGKELN KSINPDEAVA YGAAVQAAIL SGDKSENVQD LLLLDVTPLS
401 LGIETAGGVM TVLIKRNTTI PTKQTQTFTT YSDNQPGVLI QVYEGERAMT
451 KDNNLLGKFE LTGIPPAPRG VPQIEVTFDI DANGILNVSA VDKSTGKENK
501 ITITNDKGRL SKEDIERMVQ EAEKYKAEDE KQRDKVSSKN SLESYAFNMK
551 ATVEDEKLQG KINDEDKQKI LDKCNEIISW LDKNQTAEKE EFEHQQKELE
601 KVCNPIITKL YQSAGGMPGG MPGGFPGGGA PPSGGASSGP TIEEVD
```

Unformatted sequence string: **646 residues** (for pasting into other applications).

Sort by ◉ residue number ○ increasing mass ○ decreasing mass
Show ◉ matched peptides only ○ predicted peptides also

Query	Start – End	Observed	Mr(expt)	Mr(calc)	Delta	M	Score	Expect	Rank	U	Peptide
343	57 – 71	833.7900	1665.5654	1664.7828	0.7826	0	40	0.0018	1	U	K.NQVAMNPTNTVFDAK.R + Oxidation (M)
197	160 – 171	599.7600	1197.5054	1198.6670	-1.1615	0	48	0.013	1	U	K.DAGTIAGLNVLR.I
198	160 – 171	600.3600	1198.7054	1198.6670	0.0385	0	49	0.0044	1	U	K.DAGTIAGLNVLR.I
400	160 – 187	947.4500	2839.3282	2839.5443	-0.2161	1	42	0.0056	1	U	K.DAGTIAGLNVLRIINEPTAAAIAYGLDK.K
425	160 – 188	990.2700	2967.7882	2967.6393	0.1489	2	52	0.00012	1	U	K.DAGTIAGLNVLRIINEPTAAAIAYGLDKK.V
232	540 – 550	659.6000	1317.1854	1318.5863	-1.4009	0	52	0.00024	1	U	K.NSLESYAFNMK.A + Oxidation (M)

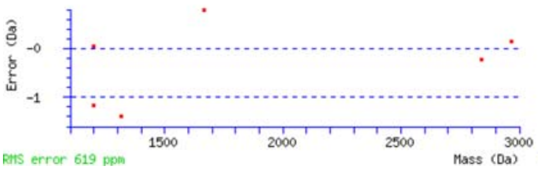

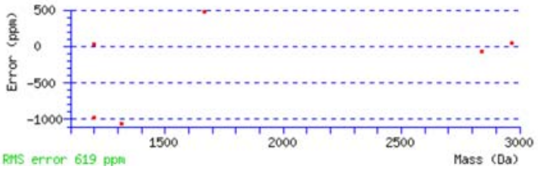


```
LOCUS       S31716                    646 aa            linear   ROD 20-AUG-1999
DEFINITION  dnaK-type molecular chaperone hsp72-ps1 - rat.
ACCESSION   S31716
VERSION     S31716
DBSOURCE    UniProtKB: locus S31716;

            summary: #length 646 #molecular-weight 70928 #checksum 4074
            ;
            genetic: #gene hsp72-ps1
            ;
            superfamily: heat shock protein 70
            ;
            PIR dates: 13-Jan-1995 #sequence_revision 13-Jan-1995 #text_change
            20-Aug-1999
            .
KEYWORDS    ATP; molecular chaperone.
SOURCE      Rattus norvegicus (Norway rat)
  ORGANISM  Rattus norvegicus
            Eukaryota; Metazoa; Chordata; Craniata; Vertebrata; Euteleostomi;
            Mammalia; Eutheria; Euarchontoglires; Glires; Rodentia; Myomorpha;
            Muroidea; Muridae; Murinae; Rattus.
REFERENCE   1  (residues 1 to 646)
  AUTHORS   Walter,L., Heine,L. and Gunther,E.
  TITLE     Sequence, expression, and mapping of a rat Mhc class Ib gene
  JOURNAL   Immunogenetics 39 (5), 351-354 (1994)
   PUBMED   7545923
FEATURES             Location/Qualifiers
     source          1..646
                     /organism="Rattus norvegicus"
                     /db_xref="taxon:10116"
     Protein         1..646
```

```
                     /product="dnaK-type molecular chaperone hsp72-ps1"
     Region          1..613
                     /region_name="PTZ00009"
                     /note="heat shock 70 kDa protein; Provisional"
                     /db_xref="CDD:240227"
     Region          6..381
                     /region_name="HSPA1-2_6-8-like_NBD"
                     /note="Nucleotide-binding domain of HSPA1-A, -B, -L,
                     HSPA-2, -6, -7, -8, and similar proteins; cd10233"
                     /db_xref="CDD:212675"
     Site            order(10,12..15,71,147,175,199..204,206,230..231,268,
                     271..272,275,338..340,342..343,366)
                     /site_type="other"
                     /note="nucleotide binding site [chemical binding]"
                     /db_xref="CDD:212675"
     Site            order(23,25,27,32..34,36,50,54,57,133..134,258,262,269,
                     272..273,276,281,283,285..286,290,342)
                     /site_type="other"
                     /note="NEF/HSP70 interaction site [polypeptide binding]"
                     /db_xref="CDD:212675"
     Site            order(33,35,57,60..61,257..258,261..262,265..266,269,
                     283..286,292,294)
                     /site_type="other"
                     /note="BAG/HSP70 interaction site [polypeptide binding]"
                     /db_xref="CDD:212675"
     Site            order(152,192,213,215..217,219..220,325)
                     /site_type="other"
                     /note="SBD interface [polypeptide binding]"
                     /db_xref="CDD:212675"
```

Mascot: http://www.matrixscience.com/

MATRIX SCIENCE MASCOT Search Results

User : pbm
E-mail :
Search title : Submitted from MRosenberg Data2009 by Mascot Daemon on SCT-14-65A4
MS data file : G:\PRESTATIONS\Labos académiques\Internationaux\Etats Unis\Martina Rosenberg\Data pour publi M Rosenberg\Identifications Mascot\MR_1233.mgf
Database : NCBIprot 20171205 (139,213,787 sequences; 51,013,024,959 residues)
Taxonomy : Rattus (77,467 sequences)
Timestamp : 25 Jan 2019 at 10:18:33 GMT

Not what you expected? Try **the peptide summary**.

Search parameters
Score distribution
Modification statistics
Legend

Protein Family Summary

Significance threshold p<	0.05	Max. number of families	20
Display non-sig. matches	☐	Dendrograms cut at	0
Preferred taxonomy	All entries		

Sensitivity

Protein families 1–5 (out of 5)

10 per page 1

1 NP_034607.3 91 60 kDa heat shock protein, mitochondrial [Mus musculus]

		Score	Mass	Matches	Sequences	emPAI
1.1	NP_034607.3<br>60 kDa heat shock protein, mitochondrial [Mus musculus]	91	60917	3 (3)	3 (3)	0.24
	**3 samesets of NP_034607.3**					
	CAA37654.1<br>unnamed protein product, partial [Rattus norvegicus]	91	57890	3 (3)	3 (3)	
	CAA38564.1<br>heat shock protein (hsp60) precursor [Rattus norvegicus]	91	60927	3 (3)	3 (3)	
	AAC53362.1<br>chaperonin 60 [Rattus norvegicus]	91	60858	3 (3)	3 (3)	

3 peptide matches (3 non-duplicate, 0 duplicate)

Query	Dupes	Observed	Mr(expt)	Mr(calc)	Delta	M	Score	Expect	Rank	U	Peptide
342		921.5800	2761.7182	2761.4143	0.3039	1	35	0.016	1	U	K.FGADARALMLQGVDLLADAVAVTMGPK.G + 2 Oxidation (M)
407		1035.4500	3103.3282	3103.6046	-0.2764	2	59	0.00013	1	U	K.DVKFGADARALMLQGVDLLADAVAVTMGPK.G + 2 Oxidation (M)
430		1073.5800	2145.1454	2144.1221	1.0233	0	57	6e-05	1	U	R.ALMLQGVDLLADAVAVTMGPK.G + 2 Oxidation (M)

**2**	NP_036767.1	85	anionic trypsin-1 precursor [Rattus norvegicus]
**3**	XP_006247453.1	54	PREDICTED: keratin, type I cytoskeletal 10 isoform X2 [Rattus norvegicus]
**4**	EDL86877.1	52	type II keratin Kb1 [Rattus norvegicus]
**5**	Q6P6Q2.1	45	RecName: Full=Keratin, type II cytoskeletal 5; AltName: Full=Cytokeratin-5; Short=CK-5; AltName: Full=Keratin-5; Short=K5; AltName...

10 per page 1

Not what you expected? Try **the peptide summary**.

Mascot: http://www.matrixscience.com/

MATRIX SCIENCE MASCOT Search Results

Protein View: CAA38564.1

heat shock protein (hsp60) precursor [Rattus norvegicus]

**Database:**	NCBIprot
**Score:**	91
**Monoisotopic mass (M_r):**	60927
**Calculated pI:**	5.91
**Taxonomy:**	Rattus norvegicus

Sequence similarity is available as an NCBI BLAST search of CAA38564.1 against nr.

Search parameters

**MS data file:**	G:\PRESTATIONS\Labos académiques\Internationaux\Etats Unis\Martina Rosenberg\Data pour publi M Rosenberg\Identifications Mascot\MR_1233.mgf
**Enzyme:**	Trypsin: cuts C-term side of KR unless next residue is P.
**Variable modifications:**	Carbamidomethyl (C), Oxidation (M)

Protein sequence coverage: 5%

Matched peptides shown in ***bold red***.

```
  1 MLRLPTVLRQ MRPVSRALAP HLTRAYAKDV KFGADARALM LQGVDLLADA
 51 VAVTMGPKGR TVIIEQSWGS PKVTKDGVTV AKSIDLKDKY KNIGAKLVQD
101 VANNTNEEAG DGTTTATVLA RSIAKEGFEK ISKGANPVEI RRGVMLAVDA
151 VIAELKKQSK PVTTPEEIAQ VATISANGDK DIGNIISDAM KKVGRKGVIT
201 VKDGKTLNDE LEIIEGMKFD RGYISPYFIN TSKGQKCEFQ DAYVLLSEKK
251 ISSVQSIVPA LEIANAHRKP LVIIAEDVDG EALSTLVLNR LKVGLQVVAV
301 KAPGFGDNRK NQLKDMAIAT GGAVFGEEGL NLNLEDVQAH DLGKVGEVIV
351 TKDDAMLLKG KGDKAHIEKR IQEITEQLDI TTSEYEKEKL NERLAKLSDG
401 VAVLKVGGTS DVEVNEKKDR VTDALNATRA AVEEGIVLGG GCALLRCIPA
451 LDSLKPANED QKIGIEIIKR ALKIPAMTIA KNAGVEGSLI VEKILQSSSE
501 VGYDAMLGDF VNMVEKGIID PTKVVRTALL DAAGVAPLLT TAEAVVTEIP
551 KEEKDPGMGA MGGMGGGMGG GMF
```

Unformatted sequence string: 573 residues (for pasting into other applications).

Sort by ◉ residue number ○ increasing mass ○ decreasing mass
Show ◉ matched peptides only ○ predicted peptides also

Query	Start – End	Observed	Mr(expt)	Mr(calc)	Delta	M	Score	Expect	Rank	U	Peptide
407	29 – 58	1035.4500	3103.3282	3103.6046	-0.2764	2	59	0.00013	1	U	K.DVKFGADARALMLQGVDLLADAVAVTMGPK.G + 2 Oxidation (
342	32 – 58	921.5800	2761.7182	2761.4143	0.3039	1	35	0.016	1	U	K.FGADARALMLQGVDLLADAVAVTMGPK.G + 2 Oxidation (M)
430	38 – 58	1073.5800	2145.1454	2144.1221	1.0233	0	57	6e-05	1	U	R.ALMLQGVDLLADAVAVTMGPK.G + 2 Oxidation (M)

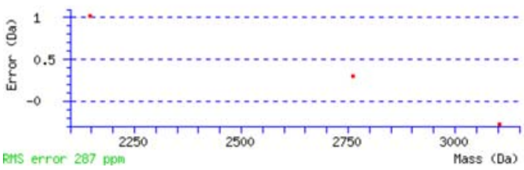

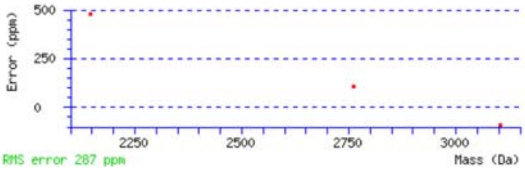

```
LOCUS       CAA38564                 573 aa            linear   ROD 06-MAY-1992
DEFINITION  heat shock protein (hsp60) precursor [Rattus norvegicus].
ACCESSION   CAA38564
VERSION     CAA38564.1
DBSOURCE    embl accession X54793.1
KEYWORDS    .
SOURCE      Rattus norvegicus (Norway rat)
  ORGANISM  Rattus norvegicus
            Eukaryota; Metazoa; Chordata; Craniata; Vertebrata; Euteleostomi;
            Mammalia; Eutheria; Euarchontoglires; Glires; Rodentia; Myomorpha;
            Muroidea; Muridae; Murinae; Rattus.
REFERENCE   1
  AUTHORS   Peralta,D., Hartman,D.J., McIntosh,A.M., Hoogenraad,N.J. and
            Hoj,P.B.
  TITLE     cDNA and deduced amino acid sequence of rat liver prehsp60
            (chaperonin-60)
  JOURNAL   Nucleic Acids Res. 18 (23), 7162 (1990)
   PUBMED   1979858
REFERENCE   2  (residues 1 to 573)
  AUTHORS   Hoej,P.B.
  TITLE     Direct Submission
  JOURNAL   Submitted (05-OCT-1990) Hoej P.B., Department of Biochemistry, La
            Trobe University, Bundoora, Victoria, 3083, AUSTRALIA
COMMENT     See X53585 for Rat kidney mRNA for heat shock protein (hsp60).
FEATURES             Location/Qualifiers
     source          1..573
                     /organism="Rattus norvegicus"
                     /strain="Wistar"
                     /db_xref="taxon:10116"
                     /cell_type="hepatocyte"
                     /tissue_type="liver"
                     /clone_lib="lambda gt10"
     Protein         1..573
                     /product="heat shock protein (hsp60) precursor"
     Region          24..555
                     /region_name="PTZ00114"
                     /note="Heat shock protein 60; Provisional"
                     /db_xref="CDD:185455"
     Region          28..548
```

```
                     /region_name="GroEL"
                     /note="GroEL_like type I chaperonin. Chaperonins are
                     involved in productive folding of proteins. They share a
                     common general morphology, a double toroid of 2 stacked
                     rings, each composed of 7-9 subunits. The symmetry of type
                     I is seven-fold and they are found...; cd03344"
                     /db_xref="CDD:239460"
     Site            order(28,32,49,60..63,65,70..71,73,83,85,93,97,100,221,
                     253,281,409,411,484,538,541..547)
                     /site_type="other"
                     /note="ring oligomerisation interface [polypeptide
                     binding]"
                     /db_xref="CDD:239460"
     Site            order(55..57,111,115,174,423,440,479,518,520)
                     /site_type="other"
                     /note="ATP/Mg binding site [chemical binding]"
                     /db_xref="CDD:239460"
     Site            order(133,459,477,486,488..489,492)
                     /site_type="other"
                     /note="stacking interactions"
                     /db_xref="CDD:239460"
     Site            order(165,210,217,400,434..435)
                     /site_type="other"
                     /note="hinge regions"
                     /db_xref="CDD:239460"
     CDS             1..573
                     /coded_by="X54793.1:7..1728"
                     /db_xref="GOA:P63039"
                     /db_xref="InterPro:IPR001844"
                     /db_xref="InterPro:IPR002423"
                     /db_xref="InterPro:IPR018370"
                     /db_xref="UniProtKB/Swiss-Prot:P63039"
```

Mascot: http://www.matrixscience.com/

MATRIX SCIENCE MASCOT Search Results

User : pbm
E-mail :
Search title : Submitted from MRosenberg Data2009 by Mascot Daemon on SCT-14-65A4
MS data file : G:\PRESTATIONS\Labos académiques\Internationaux\Etats Unis\Martina Rosenberg\Data pour publi M Rosenberg\Identifications Mascot\MR_1272.mgf
Database : NCBIprot 20171205 (139,213,787 sequences; 51,013,024,959 residues)
Taxonomy : Rattus (77,467 sequences)
Timestamp : 25 Jan 2019 at 10:20:08 GMT

Not what you expected? Try **the peptide summary**.

▶ **Search parameters**
▶ **Score distribution**
▶ **Modification statistics**
▶ **Legend**

Protein Family Summary

Significance threshold p<	0.05	Max. number of families	20
Display non-sig. matches	☐	Dendrograms cut at	0
Preferred taxonomy	All entries		

▶ **Sensitivity**

Protein families 1–5 (out of 5)

10 per page 1

▼**1** **NP_059015.1** 92 protein disulfide-isomerase A3 precursor [Rattus norvegicus]

		Score	Mass	Matches	Sequences	emPAI
1.1	**NP_059015.1**	92	56554	6 (6)	6 (6)	0.59

protein disulfide-isomerase A3 precursor [Rattus norvegicus]

▼**4 samesets of NP_059015.1**

	Score	Mass	Matches	Sequences
**P11598.2**	92	56588	6 (6)	6 (6)
**EDL79991.1**	92	53553	6 (6)	6 (6)
**EDL79992.1**	92	57043	6 (6)	6 (6)
**CAA30916.1**	92	56588	6 (6)	6 (6)

P11598.2: RecName: Full=Protein disulfide-isomerase A3; AltName: Full=58 kDa glucose-regulated protein; AltName: Full=58 kDa microsomal protein; Short=p58; AltName: Full=Disulfide isomerase E...
EDL79991.1: protein disulfide isomerase associated 3, isoform CRA_a [Rattus norvegicus]
EDL79992.1: protein disulfide isomerase associated 3, isoform CRA_b [Rattus norvegicus]
CAA30916.1: unnamed protein product [Rattus norvegicus]

▼**6 peptide matches (6 non-duplicate, 0 duplicate)**

Query	Dupes	Observed	Mr(expt)	Mr(calc)	Delta	M	Score	Expect	Rank	U	Peptide
166		551.6100	1651.8082	1651.7590	0.0491	1	50	0.0011	1	U	K.IFRDGEEAGAYDGPR.T
177		582.9700	1745.8882	1745.9213	-0.0331	1	37	0.012	1	U	K.TFLDAGHKLNFAVASR.K
190		594.7800	1187.5454	1187.5281	0.0173	0	38	0.0099	1	U	K.FVMQEEFSR.D + Oxidation (M)
191		596.2900	1190.5654	1190.5931	-0.0277	0	50	0.0017	1	U	R.LAPEYEAAATR.L
197		599.3500	1196.6854	1196.7128	-0.0274	1	42	0.011	1	U	K.LSKDPNIVIAK.M
271		698.8200	1395.6254	1395.6882	-0.0627	0	42	0.024	1	U	K.SEPIPETNEGPVK.V

▶**2**

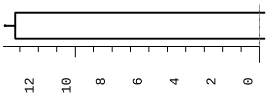

1	**NP_001008825.1**	85	keratin, type II cytoskeletal cochleal [Rattus norvegicus]
2	**NP_001008802.2**	51	keratin, type II cytoskeletal 1 [Rattus norvegicus]

▶**3** **NP_036767.1** 62 anionic trypsin-1 precursor [Rattus norvegicus]

▶**4** **XP_008770740.1** 36 PREDICTED: nuclear factor of activated T-cells, cytoplasmic 3 isoform X1 [Rattus norvegi...

▶**5** **NP_001101017.1** 34 suppression of tumorigenicity 5 protein [Rattus norvegicus]

10 per page 1

Not what you expected? Try **the peptide summary**.

Mascot: http://www.matrixscience.com/

MATRIX SCIENCE MASCOT Search Results

Protein View: P11598.2

RecName: Full=Protein disulfide-isomerase A3; AltName: Full=58 kDa glucose-regulated protein; AltName: Full=58 kDa microsomal protein; Short=p58; AltName: Full=Disulfide isomerase ER-60; AltName: Full=Endoplasmic reticulum resident protein 57; Short

**Database:**	NCBIprot
**Score:**	92
**Monoisotopic mass (M_r):**	56588
**Calculated pI:**	5.88
**Taxonomy:**	Rattus norvegicus

This protein sequence matches the following other entries:

- AAH62393.1 from **Rattus norvegicus**

Sequence similarity is available as **an NCBI BLAST search of P11598.2 against nr**.

Search parameters

**MS data file:**	G:\PRESTATIONS\Labos académiques\Internationaux\Etats Unis\Martina Rosenberg\Data pour publi M Rosenberg\Identifications Mascot\MR_1272.mgf
**Enzyme:**	Trypsin: cuts C-term side of KR unless next residue is P.
**Variable modifications:**	Carbamidomethyl (C), Oxidation (M)

Protein sequence coverage: 14%

Matched peptides shown in ***bold red***.

```
  1 MRFSCLALLP GVALLLASAL LASASDVLEL TDENFESRVS DTGSAGLMLV
 51 EFFAPWCGHC KRLAPEYEAA ATRLKGIVPL AKVDCTANTN TCNKYGVSGY
101 PTLKIFRDGE EAGAYDGPRT ADGIVSHLKK QAGPASVPLR TEDEFKKFIS
151 DKDASVVGFF RDLFSDGHSE FLKAASNLRD NYRFAHTNVE SLVKEYDDNG
201 EGITIFRPLH LANKFEDKIV AYTEKKMTSG KIKKFIQESI FGLCPHMTED
251 NKDLIQGKDL LTAYYDVDYE KNTKGSNYWR NRVMMVAKTF LDAGHKLNFA
301 VASRKTFSHE LSDFGLESTT GEIPVVAIRT AKGEKFVMQE EFSRDGKALE
351 RFLQEYFDGN LKRYLKSEPI PETNEGPVKV VVAESFDDIV NAEDKDVLIE
401 FYAPWCGHCK NLEPKYKELG EKLSKDPNIV IAKMDATAND VPSPYEVKGF
451 PTIYFSPANK KLTPKKYEGG RELNDFISYL QREATNPPII QEEKPKKKKK
501 AQEDL
```

Unformatted sequence string: **505 residues** (for pasting into other applications).

Sort by ◉ residue number ○ increasing mass ○ decreasing mass
Show ◉ matched peptides only ○ predicted peptides also

Query	Start – End	Observed	Mr(expt)	Mr(calc)	Delta	M	Score	Expect	Rank	U	Peptide
191	63 – 73	596.2900	1190.5654	1190.5931	-0.0277	0	50	0.0017	1	U	R.LAPEYEAAATR.L
166	105 – 119	551.6100	1651.8082	1651.7590	0.0491	1	50	0.0011	1	U	K.IFRDGEEAGAYDGPR.T
177	289 – 304	582.9700	1745.8882	1745.9213	-0.0331	1	37	0.012	1	U	K.TFLDAGHKLNFAVASR.K
190	336 – 344	594.7800	1187.5454	1187.5281	0.0173	0	38	0.0099	1	U	K.FVMQEEFSR.D + Oxidation (M)
271	367 – 379	698.8200	1395.6254	1395.6882	-0.0627	0	42	0.024	1	U	K.SEPIPETNEGPVK.V
197	423 – 433	599.3500	1196.6854	1196.7128	-0.0274	1	42	0.011	1	U	K.LSKDPNIVIAK.M

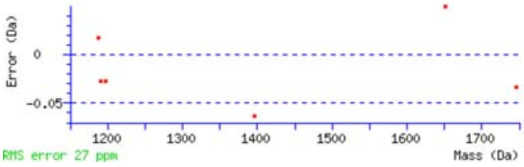

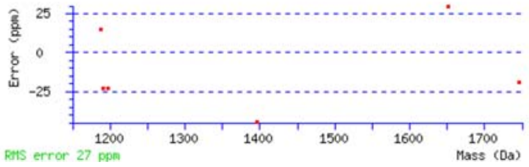


```
LOCUS       PDIA3_RAT                 505 aa            linear   ROD 16-JAN-2019
DEFINITION  RecName: Full=Protein disulfide-isomerase A3; AltName: Full=58 kDa
            glucose-regulated protein; AltName: Full=58 kDa microsomal protein;
            Short=p58; AltName: Full=Disulfide isomerase ER-60; AltName:
            Full=Endoplasmic reticulum resident protein 57; Short=ER protein
            57; Short=ERp57; AltName: Full=Endoplasmic reticulum resident
            protein 60; Short=ER protein 60; Short=ERp60; AltName: Full=HIP-70;
            AltName: Full=Q-2; Flags: Precursor.
ACCESSION   P11598
VERSION     P11598.2
DBSOURCE    UniProtKB: locus PDIA3_RAT, accession P11598;
            class: standard.
            created: Oct 1, 1989.
            sequence updated: Feb 1, 1996.
            annotation updated: Jan 16, 2019.
            xrefs: X12355.1, CAA30916.1, D63378.1, BAA09695.1, BC062393.1,
            AAH62393.1, A28807, A61354, NP_059015.1
            xrefs (non-sequence databases): UniGene:Rn.11527,
            ProteinModelPortal:P11598, SMR:P11598, BioGrid:248111,
            IntAct:P11598, MINT:P11598, STRING:10116.ENSRNOP00000020478,
            CarbonylDB:P11598, iPTMnet:P11598, PhosphoSitePlus:P11598,
            SwissPalm:P11598, World-2DPAGE:0004:P11598, jPOST:P11598,
            PaxDb:P11598, PRIDE:P11598, GeneID:29468, KEGG:rno:29468,
            UCSC:RGD:68430, CTD:2923, RGD:68430, eggNOG:KOG0190,
            eggNOG:COG0526, HOGENOM:HOG000162459, HOVERGEN:HBG005920,
            InParanoid:P11598, KO:K08056, OrthoDB:462118at2759,
            PhylomeDB:P11598, TreeFam:TF106382, PRO:PR:P11598,
            Proteomes:UP000002494, Genevisible:P11598, GO:0001669, GO:0016324,
            GO:0009986, GO:0005737, GO:0005783, GO:0005788, GO:0005576,
            GO:0005615, GO:0042470, GO:0042824, GO:0005758, GO:0005739,
            GO:0005634, GO:0005886, GO:0005790, GO:0042825, GO:0042288,
```

```
            GO:0008233, GO:0003756, GO:0019153, GO:0045454, GO:1904148,
            GO:0071560, GO:0071305, GO:0007623, GO:1903334, GO:0006457,
            GO:1901423, GO:0034976, GO:0045471, GO:0033595, GO:0055093,
            GO:0001666, GO:0002931, GO:0044321, GO:0031667, InterPro:IPR005788,
            InterPro:IPR005792, InterPro:IPR036249, InterPro:IPR017937,
            InterPro:IPR013766, Pfam:PF00085, SUPFAM:SSF52833,
            TIGRFAMs:TIGR01130, TIGRFAMs:TIGR01126, PROSITE:PS00194,
            PROSITE:PS51352
KEYWORDS    Acetylation; Complete proteome; Direct protein sequencing;
            Disulfide bond; Endoplasmic reticulum; Isomerase; Methylation;
            Phosphoprotein; Redox-active center; Reference proteome; Repeat;
            Signal.
SOURCE      Rattus norvegicus (Norway rat)
  ORGANISM  Rattus norvegicus
            Eukaryota; Metazoa; Chordata; Craniata; Vertebrata; Euteleostomi;
            Mammalia; Eutheria; Euarchontoglires; Glires; Rodentia; Myomorpha;
            Muroidea; Muridae; Murinae; Rattus.
REFERENCE   1  (residues 1 to 505)
  AUTHORS   Bennett,C.F., Balcarek,J.M., Varrichio,A. and Crooke,S.T.
  TITLE     Molecular cloning and complete amino-acid sequence of form-I
            phosphoinositide-specific phospholipase C
  JOURNAL   Nature 334 (6179), 268-270 (1988)
   PUBMED   3398923
  REMARK    NUCLEOTIDE SEQUENCE [MRNA].
REFERENCE   2  (residues 1 to 505)
  AUTHORS   Kito,M. and Urade,R.
  TITLE     Role of novel microsomal cysteine proteases
  JOURNAL   Proc. Jpn. Acad., B, Phys. Biol. Sci. 71, 189-192 (1995)
  REMARK    NUCLEOTIDE SEQUENCE [MRNA].
REFERENCE   3  (residues 1 to 505)
  AUTHORS   Gerhard,D.S., Wagner,L., Feingold,E.A., Shenmen,C.M., Grouse,L.H.,
            Schuler,G., Klein,S.L., Old,S., Rasooly,R., Good,P., Guyer,M.,
            Peck,A.M., Derge,J.G., Lipman,D., Collins,F.S., Jang,W., Sherry,S.,
            Feolo,M., Misquitta,L., Lee,E., Rotmistrovsky,K., Greenhut,S.F.,
            Schaefer,C.F., Buetow,K., Bonner,T.I., Haussler,D., Kent,J.,
            Kiekhaus,M., Furey,T., Brent,M., Prange,C., Schreiber,K.,
            Shapiro,N., Bhat,N.K., Hopkins,R.F., Hsie,F., Driscoll,T.,
            Soares,M.B., Casavant,T.L., Scheetz,T.E., Brown-stein,M.J.,
            Usdin,T.B., Toshiyuki,S., Carninci,P., Piao,Y., Dudekula,D.B.,
            Ko,M.S., Kawakami,K., Suzuki,Y., Sugano,S., Gruber,C.E.,
            Smith,M.R., Simmons,B., Moore,T., Waterman,R., Johnson,S.L.,
            Ruan,Y., Wei,C.L., Mathavan,S., Gunaratne,P.H., Wu,J., Garcia,A.M.,
            Hulyk,S.W., Fuh,E., Yuan,Y., Sneed,A., Kowis,C., Hodgson,A.,
            Muzny,D.M., McPherson,J., Gibbs,R.A., Fahey,J., Helton,E.,
            Ketteman,M., Madan,A., Rodrigues,S., Sanchez,A., Whiting,M.,
            Madari,A., Young,A.C., Wetherby,K.D., Granite,S.J., Kwong,P.N.,
            Brinkley,C.P., Pearson,R.L., Bouffard,G.G., Blakesly,R.W.,
            Green,E.D., Dickson,M.C., Rodriguez,A.C., Grimwood,J., Schmutz,J.,
            Myers,R.M., Butterfield,Y.S., Griffith,M., Griffith,O.L.,
            Krzywinski,M.I., Liao,N., Morin,R., Palmquist,D., Petrescu,A.S.,
            Skalska,U., Smailus,D.E., Stott,J.M., Schnerch,A., Schein,J.E.,
            Jones,S.J., Holt,R.A., Baross,A., Marra,M.A., Clifton,S.,
            Makowski,K.A., Bosak,S. and Malek,J.
  CONSRTM   MGC Project Team
  TITLE     The status, quality, and expansion of the NIH full-length cDNA
            project: the Mammalian Gene Collection (MGC)
  JOURNAL   Genome Res. 14 (10B), 2121-2127 (2004)
   PUBMED   15489334
  REMARK    NUCLEOTIDE SEQUENCE [LARGE SCALE MRNA].;
            TISSUE=Prostate
            Erratum:[Genome Res. 2006 Jun;16(6):804. Morrin, Ryan [corrected to
            Morin, Ryan]]
REFERENCE   4  (residues 1 to 505)
  AUTHORS   Martin,J.L., Pumford,N.R., LaRosa,A.C., Martin,B.M., Gonzaga,H.M.,
            Beaven,M.A. and Pohl,L.R.
  TITLE     A metabolite of halothane covalently binds to an endoplasmic
            reticulum protein that is highly homologous to
            phosphatidylinositol-specific phospholipase C-alpha but has no
            activity
  JOURNAL   Biochem. Biophys. Res. Commun. 178 (2), 679-685 (1991)
   PUBMED   1650195
  REMARK    PARTIAL PROTEIN SEQUENCE.;
            TISSUE=Liver
REFERENCE   5  (residues 1 to 505)
  AUTHORS   Mobbs,C.V., Fink,G. and Pfaff,D.W.
  TITLE     HIP-70: a protein induced by estrogen in the brain and LH-RH in the
            pituitary
  JOURNAL   Science 247 (4949 Pt 1), 1477-1479 (1990)
   PUBMED   2181662
  REMARK    PROTEIN SEQUENCE OF 26-43.;
            TISSUE=Brain, and Pituitary
REFERENCE   6  (residues 1 to 505)
  AUTHORS   Srivastava,S.P., Chen,N.Q., Liu,Y.X. and Holtzman,J.L.
  TITLE     Purification and characterization of a new isozyme of
            thiol:protein-disulfide oxidoreductase from rat hepatic microsomes.
            Relationship of this isozyme to cytosolic
            phosphatidylinositol-specific phospholipase C form 1A
  JOURNAL   J. Biol. Chem. 266 (30), 20337-20344 (1991)
   PUBMED   1657921
  REMARK    PROTEIN SEQUENCE OF 25-54; 258-269; 285-310; 347-350; 412-419 AND
            434-463.;
            TISSUE=Liver
REFERENCE   7  (residues 1 to 505)
  AUTHORS   Urade,R., Nasu,M., Moriyama,T., Wada,K. and Kito,M.
  TITLE     Protein degradation by the phosphoinositide-specific phospholipase
            C-alpha family from rat liver endoplasmic reticulum
  JOURNAL   J. Biol. Chem. 267 (21), 15152-15159 (1992)
   PUBMED   1321829
  REMARK    PROTEIN SEQUENCE OF 26-34; 174-193; 433-446 AND 448-458.
REFERENCE   8  (residues 1 to 505)
  AUTHORS   Lubec,G. and Afjehi-Sadat,L.
  TITLE     Direct Submission
  JOURNAL   Submitted (??-DEC-2006) to UniProtKB
  REMARK    PROTEIN SEQUENCE OF 105-119; 148-161; 184-214; 259-271; 306-329;
            336-344; 352-363; 449-460 AND 472-482, AND IDENTIFICATION BY MASS
            SPECTROMETRY.;
            STRAIN=Sprague-Dawley; TISSUE=Spinal cord
REFERENCE   9  (residues 1 to 505)
  AUTHORS   Urade,R., Oda,T., Ito,H., Moriyama,T., Utsumi,S. and Kito,M.
  TITLE     Functions of characteristic Cys-Gly-His-Cys (CGHC) and
            Gln-Glu-Asp-Leu (QEDL) motifs of microsomal ER-60 protease
  JOURNAL   J. Biochem. 122 (4), 834-842 (1997)
   PUBMED   9399589
```

```
  REMARK    MUTAGENESIS OF 502-GLN--LEU-505.
REFERENCE   10 (residues 1 to 505)
  AUTHORS   Urade,R. and Kito,M.
  TITLE     Inhibition by acidic phospholipids of protein degradation by ER-60
            protease, a novel cysteine protease, of endoplasmic reticulum
  JOURNAL   FEBS Lett. 312 (1), 83-86 (1992)
   PUBMED   1330685
  REMARK    INHIBITION BY PHOSPHOLIPIDS.
REFERENCE   11 (residues 1 to 505)
  AUTHORS   Kameshwari,D.B., Bhande,S., Sundaram,C.S., Kota,V., Siva,A.B. and
            Shivaji,S.
  TITLE     Glucose-regulated protein precursor (GRP78) and tumor rejection
            antigen (GP96) are unique to hamster caput epididymal spermatozoa
  JOURNAL   Asian J. Androl. 12 (3), 344-355 (2010)
   PUBMED   20400973
  REMARK    TISSUE SPECIFICITY.
COMMENT     On or before Mar 15, 2005 this sequence version replaced gi:91897,
            gi:130233.
            [CATALYTIC ACTIVITY] Reaction=Catalyzes the rearrangement of -S-S-
            bonds in proteins.; EC=5.3.4.1.
            ACTIVITY REGULATION: Seems to be inhibited by acidic phospholipids.
            [SUBUNIT] Subunit of the TAP complex, also known as the peptide
            loading complex (PLC). Can form disulfide-linked heterodimers with
            TAPBP. Interacts with ERP27 and CANX (By similarity). Interacts
            with MZB1 in a calcium-dependent manner (By similarity). Interacts
            with SERPINA2 and with the S and Z variants of SERPINA1. Interacts
            with ATP2A2 (By similarity). {ECO:0000250|UniProtKB:P27773,
            ECO:0000250|UniProtKB:P30101}.
            [SUBCELLULAR LOCATION] Endoplasmic reticulum
            {ECO:0000250|UniProtKB:P30101}. Endoplasmic reticulum lumen
            {ECO:0000250}. Melanosome {ECO:0000250|UniProtKB:P30101}.
            [TISSUE SPECIFICITY] In caput epididymal spermatozoa, detected in
            the head, mid and principal pieces. In cauda epididymal spermatozoa
            detected only in the acrosome (at protein level).
            {ECO:0000269|PubMed:20400973}.
            [SIMILARITY] Belongs to the protein disulfide isomerase family.
            {ECO:0000305}.
            [CAUTION] Was originally thought to be a phosphatidyl-inositol
            4,5-bisphosphate phosphodiesterase type I (phospholipase C-alpha)
            then was thought (PubMed:1321829 and PubMed:1330685) to be a thiol
            protease. {ECO:0000305|PubMed:3398923}.
FEATURES             Location/Qualifiers
     source          1..505
                     /organism="Rattus norvegicus"
                     /db_xref="taxon:10116"
     gene            1..505
                     /gene="Pdia3"
                     /gene_synonym="Erp60"
                     /gene_synonym="Grp58"
     Protein         1..505
                     /product="Protein disulfide-isomerase A3"
                     /EC_number="5.3.4.1"
                     /note="58 kDa glucose-regulated protein; 58 kDa microsomal
                     protein; Disulfide isomerase ER-60; Endoplasmic reticulum
                     resident protein 57; Endoplasmic reticulum resident
                     protein 60; HIP-70; Q-2; p58; ER protein 57; ERp57; ER
                     protein 60; ERp60"
                     /UniProtKB_evidence="Evidence at protein level"
     Region          1..24
                     /region_name="Signal"
                     /experiment="experimental evidence, no additional details
                     recorded"
                     /note="{ECO:0000269|PubMed:1657921}."
     Region          1..13
                     /region_name="Conflict"
                     /experiment="experimental evidence, no additional details
                     recorded"
                     /note="MRFSCLALLPGVA -> MPSAALRCSRAWR (in Ref. 1).
                     {ECO:0000305}."
     Region          25..505
                     /region_name="Mature chain"
                     /experiment="experimental evidence, no additional details
                     recorded"
                     /note="Protein disulfide-isomerase A3.
                     /FTId=PRO_0000034227."
     Region          25..133
                     /region_name="Domain"
                     /experiment="experimental evidence, no additional details
                     recorded"
                     /note="Thioredoxin 1.
                     {ECO:0000255|PROSITE-ProRule:PRU00691}."
     Region          26..487
                     /region_name="ER_PDI_fam"
                     /note="protein disulfide isomerase, eukaryotic; TIGR01130"
                     /db_xref="CDD:273457"
     Region          27..131
                     /region_name="Thioredoxin"
                     /note="Thioredoxin; pfam00085"
                     /db_xref="CDD:278513"
     Bond            bond(57,60)
                     /bond_type="disulfide"
                     /experiment="experimental evidence, no additional details
                     recorded"
                     /note="Redox-active.
                     {ECO:0000255|PROSITE-ProRule:PRU00691}."
     Bond            bond(57)
                     /bond_type="disulfide"
                     /experiment="experimental evidence, no additional details
                     recorded"
                     /note="Interchain (with C-115 in TAPBP); in linked form.
                     {ECO:0000250}."
     Site            57
                     /site_type="active"
                     /experiment="experimental evidence, no additional details
                     recorded"
                     /note="Nucleophile. {ECO:0000250}."
     Site            58
                     /site_type="other"
                     /experiment="experimental evidence, no additional details
                     recorded"
                     /note="Contributes to redox potential value.
                     {ECO:0000250}."
     Site            59
```

```
                     /site_type="other"
                     /experiment="experimental evidence, no additional details
                     recorded"
                     /note="Contributes to redox potential value.
                     {ECO:0000250}."
     Site            60
                     /site_type="active"
                     /experiment="experimental evidence, no additional details
                     recorded"
                     /note="Nucleophile. {ECO:0000250}."
     Site            61
                     /site_type="methylation"
                     /experiment="experimental evidence, no additional details
                     recorded"
                     /note="N6-methyllysine. {ECO:0000250|UniProtKB:P30101}."
     Bond            bond(85,92)
                     /bond_type="disulfide"
                     /experiment="experimental evidence, no additional details
                     recorded"
                     /note="{ECO:0000250}."
     Region          98
                     /region_name="Conflict"
                     /experiment="experimental evidence, no additional details
                     recorded"
                     /note="S -> T (in Ref. 1; CAA30916). {ECO:0000305}."
     Site            119
                     /site_type="other"
                     /experiment="experimental evidence, no additional details
                     recorded"
                     /note="Lowers pKa of C-terminal Cys of first active site.
                     {ECO:0000250}."
     Site            129
                     /site_type="modified"
                     /experiment="experimental evidence, no additional details
                     recorded"
                     /note="N6-succinyllysine. {ECO:0000250|UniProtKB:P27773}."
     Region          135..240
                     /region_name="PDI_b_ERp57"
                     /note="PDIb family, ERp57 subfamily, first redox inactive
                     TRX-like domain b; ERp57 (or ERp60) exhibits both
                     disulfide oxidase and reductase functions like PDI, by
                     catalyzing the formation of disulfide bonds of newly
                     synthesized polypeptides in the ER and acting...; cd03069"
                     /db_xref="CDD:239367"
     Site            152
                     /site_type="acetylation"
                     /experiment="experimental evidence, no additional details
                     recorded"
                     /note="N6-acetyllysine. {ECO:0000250|UniProtKB:P27773}."
     Site            218
                     /site_type="modified"
                     /experiment="experimental evidence, no additional details
                     recorded"
                     /note="N6-succinyllysine. {ECO:0000250|UniProtKB:P27773}."
     Region          232..240
                     /region_name="Conflict"
                     /experiment="experimental evidence, no additional details
                     recorded"
                     /note="IKKFIQESI -> SRSLFRKA (in Ref. 1; CAA30916).
                     {ECO:0000305}."
     Region          244..357
                     /region_name="PDI_b'_ERp72_ERp57"
                     /note="PDIb' family, ERp72 and ERp57 subfamily, second
                     redox inactive TRX-like domain b'; ERp72 and ER57 are
                     involved in oxidative protein folding in the ER, like PDI.
                     They exhibit both disulfide oxidase and reductase
                     functions, by catalyzing the formation of...; cd03073"
                     /db_xref="CDD:239371"
     Site            252
                     /site_type="acetylation"
                     /experiment="experimental evidence, no additional details
                     recorded"
                     /note="N6-acetyllysine. {ECO:0000250|UniProtKB:P27773}."
     Site            319
                     /site_type="phosphorylation"
                     /experiment="experimental evidence, no additional details
                     recorded"
                     /note="Phosphothreonine. {ECO:0000250|UniProtKB:P30101}."
     Region          343..485
                     /region_name="Domain"
                     /experiment="experimental evidence, no additional details
                     recorded"
                     /note="Thioredoxin 2.
                     {ECO:0000255|PROSITE-ProRule:PRU00691}."
     Site            362
                     /site_type="acetylation"
                     /experiment="experimental evidence, no additional details
                     recorded"
                     /note="N6-acetyllysine. {ECO:0000250|UniProtKB:P27773}."
     Region          377..480
                     /region_name="PDI_a_PDI_a'_C"
                     /note="PDIa family, C-terminal TRX domain (a') subfamily;
                     composed of the C-terminal redox active a' domains of PDI,
                     ERp72, ERp57 (or ERp60) and EFP1. PDI, ERp72 and ERp57 are
                     endoplasmic reticulum (ER)-resident eukaryotic proteins
                     involved in oxidative protein...; cd02995"
                     /db_xref="CDD:239293"
     Site            order(406,409,471)
                     /site_type="active"
                     /note="catalytic residues [active]"
                     /db_xref="CDD:239293"
     Bond            bond(406,409)
                     /bond_type="disulfide"
                     /experiment="experimental evidence, no additional details
                     recorded"
                     /note="Redox-active.
                     {ECO:0000255|PROSITE-ProRule:PRU00691}."
     Site            406
                     /site_type="active"
                     /experiment="experimental evidence, no additional details
                     recorded"
                     /note="Nucleophile. {ECO:0000250}."
     Site            407
```

```
                    /site_type="other"
                    /experiment="experimental evidence, no additional details
                    recorded"
                    /note="Contributes to redox potential value.
                    {ECO:0000250}."
Site                408
                    /site_type="other"
                    /experiment="experimental evidence, no additional details
                    recorded"
                    /note="Contributes to redox potential value.
                    {ECO:0000250}."
Site                409
                    /site_type="active"
                    /experiment="experimental evidence, no additional details
                    recorded"
                    /note="Nucleophile. {ECO:0000250}."
Site                471
                    /site_type="other"
                    /experiment="experimental evidence, no additional details
                    recorded"
                    /note="Lowers pKa of C-terminal Cys of second active site.
                    {ECO:0000250}."
Region              476
                    /region_name="Conflict"
                    /experiment="experimental evidence, no additional details
                    recorded"
                    /note="F -> L (in Ref. 2; BAA09695). {ECO:0000305}."
Site                494
                    /site_type="acetylation"
                    /experiment="experimental evidence, no additional details
                    recorded"
                    /note="N6-acetyllysine. {ECO:0000250|UniProtKB:P27773}."
Region              502..505
                    /region_name="Short sequence motif of biological interest"
                    /experiment="experimental evidence, no additional details
                    recorded"
                    /note="Prevents secretion from ER."
Site                502..505
                    /site_type="mutagenized"
                    /experiment="experimental evidence, no additional details
                    recorded"
                    /note="Missing: Failure to prevent secretion from ER.
                    {ECO:0000269|PubMed:9399589}."
Site                502..505
                    /site_type="mutagenized"
                    /experiment="experimental evidence, no additional details
                    recorded"
                    /note="QEDL->AAGL: Failure to prevent secretion from ER.
                    {ECO:0000269|PubMed:9399589}."
```

Mascot: http://www.matrixscience.com/

MATRIX SCIENCE MASCOT Search Results

User : pbm
E-mail :
Search title : Submitted from MRosenberg Data2009 by Mascot Daemon on SCT-14-65A4
MS data file : G:\PRESTATIONS\Labos académiques\Internationaux\Etats Unis\Martina Rosenberg\Data pour publi M Rosenberg\Identifications Mascot\MR_1193.mgf
Database : NCBIprot 20171205 (139,213,787 sequences; 51,013,024,959 residues)
Taxonomy : Rattus (77,467 sequences)
Timestamp : 25 Jan 2019 at 10:17:00 GMT

Not what you expected? Try **the peptide summary**.

Search parameters
Score distribution
Modification statistics
Legend

Protein Family Summary

Significance threshold p<	0.05	Max. number of families	20
Display non-sig. matches	☐	Dendrograms cut at	0
Preferred taxonomy	All entries		

Sensitivity

Protein families 1–6 (out of 6)

10 per page 1

1 **NP_034249.1** 76 EH domain-containing protein 1 [Mus musculus]

		Score	Mass	Matches	Sequences	emPAI
1.1	NP_034249.1	76	60565	2 (2)	2 (2)	0.15

EH domain-containing protein 1 [Mus musculus]

1 sameset of NP_034249.1

2 peptide matches (2 non-duplicate, 0 duplicate)

Query	Dupes	Observed	Mr(expt)	Mr(calc)	Delta	M	Score	Expect	Rank	U	Peptide
302		742.9700	1483.9254	1482.7024	1.2230	0	46	0.0073	1	U	K.MQELLQTQDFSK.F + Oxidation (M)
366		845.8300	1689.6454	1689.8243	-0.1789	0	67	7e-05	1	U	K.LLDTVDDMLANDIAR.L + Oxidation (M)

2	NP_001008825.1	59	keratin, type II cytoskeletal cochleal [Rattus norvegicus]
3	NP_036767.1	57	anionic trypsin-1 precursor [Rattus norvegicus]
4	XP_008763925.1	44	PREDICTED: keratin, type II cytoskeletal 73 isoform X1 [Rattus norvegicus]
5	AAI28704.1	41	Pgm1 protein, partial [Rattus norvegicus]
6	EDM00707.1	29	lamin A, isoform CRA_a [Rattus norvegicus]

10 per page 1

Not what you expected? Try **the peptide summary**.

Mascot: http://www.matrixscience.com/

MATRIX SCIENCE MASCOT Search Results

Protein View: NP_034249.1

EH domain-containing protein 1 [Mus musculus]

**Database:**	NCBIprot
**Score:**	76
**Monoisotopic mass (M_r):**	60565
**Calculated pI:**	6.35
**Taxonomy:**	**Mus musculus**

This protein sequence matches the following other entries:

- NP_001011939.1 from **Rattus norvegicus**
- XP_021008206.1 from **Mus caroli**
- Q9WVK4.1 from **Mus musculus**
- Q641Z6.1 from **Rattus norvegicus**
- AAD45423.1 from **Mus musculus**
- AAF24223.1 from **Mus musculus**
- BAB28540.1 from **Mus musculus**
- BAC40684.1 from **Mus musculus**
- AAH43332.2 from **Mus musculus**
- AAH82030.1 from **Rattus norvegicus**
- BAE38956.1 from **Mus musculus**
- BAE32742.1 from **Mus musculus**
- BAE43057.1 from **Mus musculus**
- BAE35499.1 from **Mus musculus**
- AAH54828.2 from **Mus musculus**
- EDL33232.1 from **Mus musculus**
- EDM12590.1 from **Rattus norvegicus**

Sequence similarity is available as **an NCBI BLAST search of NP_034249.1 against nr**.

Search parameters

**MS data file:**	G:\PRESTATIONS\Labos académiques\Internationaux\Etats Unis\Martina Rosenberg\Data pour publi M Rosenberg\Identifications Mascot\MR_1193.mgf
**Enzyme:**	Trypsin: cuts C-term side of KR unless next residue is P.
**Variable modifications:**	**Carbamidomethyl (C)**, **Oxidation (M)**

Protein sequence coverage: 5%

Matched peptides shown in ***bold red***.

```
  1 MFSWVSKDAR RKKEPELFQT VAEGLRQLYA QKLLPLEEHY RFHEFHSPAL
 51 EDADFDNKPM VLLVGQYSTG KTTFIRHLIE QDFPGMRIGP EPTTDSFIAV
101 MHGPTEGVVP GNALVVDPRR PFRKLNAFGN AFLNRFMCAQ LPNPVLDSIS
151 IIDTPGILSG EKQRISRGYD FAAVLEWFAE RVDRIILLFD AHKLDISDEF
201 SEVIKALKNH EDKIRVVLNK ADQIETQQLM RVYGALMWSL GKIINTPEVV
251 RVYIGSFWSH PLLIPDNRKL FEAEEQDLFK DIQSLPRNAA LRKLNDLIKR
301 ARLAKVHAYI ISSLKKEMPN VFGKESKKKE LVNNLGEIYQ KIEREHQISS
351 GDFPSLRKMQ ELLQTQDFSK FQALKPKLLD TVDDMLANDI ARLMVMVRQE
401 ESLMPSQAVK GGAFDGTMNG PFGHGYGEGA GEGIDDVEWV VGKDKPTYDE
451 IFYTLSPVNG KITGANAKKE MVKSKLPNTV LGKIWKLADV DKDGLLDDEE
501 FALANHLIKV KLEGHELPAD LPPHLIPPSK RRHE
```

Unformatted sequence string: **534 residues** (for pasting into other applications).

Sort by ◉ residue number ○ increasing mass ○ decreasing mass
Show ◉ matched peptides only ○ predicted peptides also

Query	Start – End	Observed	Mr(expt)	Mr(calc)	Delta	M	Score	Expect	Rank	U	Peptide
302	359 – 370	742.9700	1483.9254	1482.7024	1.2230	0	46	0.0073	1	U	K.MQELLQTQDFSK.F + Oxidation (M)
366	378 – 392	845.8300	1689.6454	1689.8243	-0.1789	0	67	7e-05	1	U	K.LLDTVDDMLANDIAR.L + Oxidation (M)

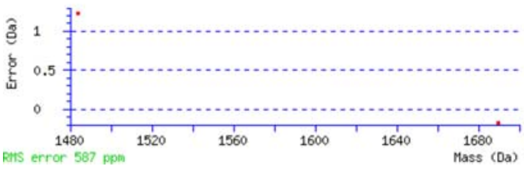

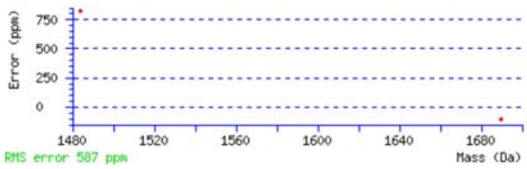


```
LOCUS       NP_034249                534 aa            linear   ROD 15-DEC-2018
DEFINITION  EH domain-containing protein 1 [Mus musculus].
ACCESSION   NP_034249
VERSION     NP_034249.1
DBSOURCE    REFSEQ: accession NM_010119.5
KEYWORDS    RefSeq.
SOURCE      Mus musculus (house mouse)
  ORGANISM  Mus musculus
            Eukaryota; Metazoa; Chordata; Craniata; Vertebrata; Euteleostomi;
            Mammalia; Eutheria; Euarchontoglires; Glires; Rodentia; Myomorpha;
            Muroidea; Muridae; Murinae; Mus; Mus.
REFERENCE   1  (residues 1 to 534)
  AUTHORS   Iseka FM, Goetz BT, Mushtaq I, An W, Cypher LR, Bielecki TA, Tom
            EC, Arya P, Bhattacharyya S, Storck MD, Semerad CL, Talmadge JE,
            Mosley RL, Band V and Band H.
  TITLE     Role of the EHD Family of Endocytic Recycling Regulators for TCR
            Recycling and T Cell Function
  JOURNAL   J. Immunol. 200 (2), 483-499 (2018)
   PUBMED   29212907
REFERENCE   2  (residues 1 to 534)
```

```
  AUTHORS   Yeow I, Howard G, Chadwick J, Mendoza-Topaz C, Hansen CG, Nichols
            BJ and Shvets E.
  TITLE     EHD Proteins Cooperate to Generate Caveolar Clusters and to
            Maintain Caveolae during Repeated Mechanical Stress
  JOURNAL   Curr. Biol. 27 (19), 2951-2962 (2017)
   PUBMED   28943089
  REMARK    GeneRIF: EHD1, EHD2, and EHD4 are recruited to caveolae.
            Recruitment of the other EHDs increases markedly when EHD2, which
            has been previously detected at caveolae, is absent. Construction
            of knockout cell lines lacking EHDs 1, 2, and 4 confirms this
            apparent functional redundancy.
REFERENCE   3  (residues 1 to 534)
  AUTHORS   Cypher LR, Bielecki TA, Adepegba O, Huang L, An W, Iseka F, Luan H,
            Tom E, Storck MD, Hoppe AD, Band V and Band H.
  TITLE     CSF-1 receptor signalling is governed by pre-requisite EHD1
            mediated receptor display on the macrophage cell surface
  JOURNAL   Cell. Signal. 28 (9), 1325-1335 (2016)
   PUBMED   27224507
  REMARK    GeneRIF: These findings reveal a novel and functionally important
            role for EHD1 in governing CSF-1R signalling via regulation of
            anterograde transport of CSF-1R to the macrophage cell surface.
            Erratum:[Cell Signal. 2016 Dec;28(12):1933. PMID: 27510742]
REFERENCE   4  (residues 1 to 534)
  AUTHORS   Bhattacharyya S, Rainey MA, Arya P, Mohapatra BC, Mushtaq I, Dutta
            S, George M, Storck MD, McComb RD, Muirhead D, Todd GL, Gould K,
            Datta K, Gelineau-van Waes J, Band V and Band H.
  TITLE     Endocytic recycling protein EHD1 regulates primary cilia
            morphogenesis and SHH signaling during neural tube development
  JOURNAL   Sci Rep 6, 20727 (2016)
   PUBMED   26884322
  REMARK    Erratum:[Sci Rep. 2017 Mar 23;7:42320. PMID: 28332491]
            Publication Status: Online-Only
REFERENCE   5  (residues 1 to 534)
  AUTHORS   Arya P, Rainey MA, Bhattacharyya S, Mohapatra BC, George M, Kuracha
            MR, Storck MD, Band V, Govindarajan V and Band H.
  TITLE     The endocytic recycling regulatory protein EHD1 Is required for
            ocular lens development
  JOURNAL   Dev. Biol. 408 (1), 41-55 (2015)
   PUBMED   26455409
  REMARK    GeneRIF: these data reveal a unique role for EHD1 in early lens
            development.
REFERENCE   6  (residues 1 to 534)
  AUTHORS   Guilherme A, Soriano NA, Furcinitti PS and Czech MP.
  TITLE     Role of EHD1 and EHBP1 in perinuclear sorting and insulin-regulated
            GLUT4 recycling in 3T3-L1 adipocytes
  JOURNAL   J. Biol. Chem. 279 (38), 40062-40075 (2004)
   PUBMED   15247266
  REMARK    GeneRIF: EHD1 and EHBP1, but not EHD2, are required for perinuclear
            localization of GLUT4 and reveal that loss of EHBP1 disrupts
            insulin-regulated GLUT4 recycling in cultured adipocytes.
REFERENCE   7  (residues 1 to 534)
  AUTHORS   Galperin E, Benjamin S, Rapaport D, Rotem-Yehudar R, Tolchinsky S
            and Horowitz M.
  TITLE     EHD3: a protein that resides in recycling tubular and vesicular
            membrane structures and interacts with EHD1
  JOURNAL   Traffic 3 (8), 575-589 (2002)
   PUBMED   12121420
  REMARK    GeneRIF: EHD3: a protein that resides in recycling tubular and
            vesicular membrane structures and interacts with EHD1.
REFERENCE   8  (residues 1 to 534)
  AUTHORS   Lin SX, Grant B, Hirsh D and Maxfield FR.
  TITLE     Rme-1 regulates the distribution and function of the endocytic
            recycling compartment in mammalian cells
  JOURNAL   Nat. Cell Biol. 3 (6), 567-572 (2001)
   PUBMED   11389441
REFERENCE   9  (residues 1 to 534)
  AUTHORS   Pohl U, Smith JS, Tachibana I, Ueki K, Lee HK, Ramaswamy S, Wu Q,
            Mohrenweiser HW, Jenkins RB and Louis DN.
  TITLE     EHD2, EHD3, and EHD4 encode novel members of a highly conserved
            family of EH domain-containing proteins
  JOURNAL   Genomics 63 (2), 255-262 (2000)
   PUBMED   10673336
REFERENCE   10 (residues 1 to 534)
  AUTHORS   Mintz L, Galperin E, Pasmanik-Chor M, Tulzinsky S, Bromberg Y,
            Kozak CA, Joyner A, Fein A and Horowitz M.
  TITLE     EHD1--an EH-domain-containing protein with a specific expression
            pattern
  JOURNAL   Genomics 59 (1), 66-76 (1999)
   PUBMED   10395801
COMMENT     PROVISIONAL REFSEQ: This record has not yet been subject to final
            NCBI review. The reference sequence was derived from AC127556.4.

            Sequence Note: The RefSeq transcript and protein were derived from
            genomic sequence to make the sequence consistent with the reference
            genome assembly. The genomic coordinates used for the transcript
            record were based on alignments.

            Publication Note:  This RefSeq record includes a subset of the
            publications that are available for this gene. Please see the Gene
            record to access additional publications.

            ##Evidence-Data-START##
            Transcript exon combination :: AF099186.1, AK154651.1 [ECO:0000332]
            RNAseq introns              :: single sample supports all introns
                                           SAMN00849374, SAMN00849375
                                           [ECO:0000348]
            ##Evidence-Data-END##
FEATURES             Location/Qualifiers
     source          1..534
                     /organism="Mus musculus"
                     /strain="C57BL/6"
                     /db_xref="taxon:10090"
                     /chromosome="19"
                     /map="19 4.4 cM"
     Protein         1..534
                     /product="EH domain-containing protein 1"
                     /note="mPAST1; PAST homolog 1"
                     /calculated_mol_wt=60472
     Site            1
                     /site_type="other"
                     /experiment="experimental evidence, no additional details
                     recorded"
                     /note="N-acetylmethionine. {ECO:0000250|UniProtKB:Q9H4M9};
```

```
                 propagated from UniProtKB/Swiss-Prot (Q9WVK4.1)"
Region           25..56
                 /region_name="EHD_N"
                 /note="N-terminal EH-domain containing protein; pfam16880"
                 /db_xref="CDD:318972"
Region           60..300
                 /region_name="EHD"
                 /note="Eps15 homology domain (EHD), C-terminal domain;
                 cd09913"
                 /db_xref="CDD:206740"
Region           65..72
                 /region_name="G1 motif.
                 {ECO:0000255|PROSITE-ProRule:PRU01055}"
                 /experiment="experimental evidence, no additional details
                 recorded"
                 /note="propagated from UniProtKB/Swiss-Prot (Q9WVK4.1)"
Site             65..72
                 /site_type="other"
                 /note="G1 box"
                 /db_xref="CDD:206740"
Site             order(70..73,153,220,222,256..258)
                 /site_type="other"
                 /note="GTP/Mg2+ binding site [chemical binding]"
                 /db_xref="CDD:206740"
Region           91..92
                 /region_name="G2 motif.
                 {ECO:0000255|PROSITE-ProRule:PRU01055}"
                 /experiment="experimental evidence, no additional details
                 recorded"
                 /note="propagated from UniProtKB/Swiss-Prot (Q9WVK4.1)"
Site             94
                 /site_type="other"
                 /note="G2 box"
                 /db_xref="CDD:206740"
Site             98..100
                 /site_type="other"
                 /note="Switch I region"
                 /db_xref="CDD:206740"
Region           153..156
                 /region_name="G3 motif.
                 {ECO:0000255|PROSITE-ProRule:PRU01055}"
                 /experiment="experimental evidence, no additional details
                 recorded"
                 /note="propagated from UniProtKB/Swiss-Prot (Q9WVK4.1)"
Site             153..156
                 /site_type="other"
                 /note="G3 box"
                 /db_xref="CDD:206740"
Site             order(155..156,183..184)
                 /site_type="other"
                 /note="Switch II region"
                 /db_xref="CDD:206740"
Region           219..222
                 /region_name="G4 motif.
                 {ECO:0000255|PROSITE-ProRule:PRU01055}"
                 /experiment="experimental evidence, no additional details
                 recorded"
                 /note="propagated from UniProtKB/Swiss-Prot (Q9WVK4.1)"
Site             219..222
                 /site_type="other"
                 /note="G4 box"
                 /db_xref="CDD:206740"
Region           243
                 /region_name="G5 motif.
                 {ECO:0000255|PROSITE-ProRule:PRU01055}"
                 /experiment="experimental evidence, no additional details
                 recorded"
                 /note="propagated from UniProtKB/Swiss-Prot (Q9WVK4.1)"
Site             256..258
                 /site_type="other"
                 /note="G5 box"
                 /db_xref="CDD:206740"
Site             355
                 /site_type="other"
                 /experiment="experimental evidence, no additional details
                 recorded"
                 /note="Phosphoserine. {ECO:0000244|PubMed:21183079};
                 propagated from UniProtKB/Swiss-Prot (Q9WVK4.1)"
Region           438..531
                 /region_name="EH"
                 /note="Eps15 homology domain; smart00027"
                 /db_xref="CDD:197477"
Site             456
                 /site_type="other"
                 /experiment="experimental evidence, no additional details
                 recorded"
                 /note="Phosphoserine. {ECO:0000244|PubMed:21183079};
                 propagated from UniProtKB/Swiss-Prot (Q9WVK4.1)"
CDS              1..534
                 /gene="Ehd1"
                 /gene_synonym="AA409636; Past1; RME-1"
                 /coded_by="NM_010119.5:80..1684"
                 /db_xref="CCDS:CCDS29501.1"
                 /db_xref="GeneID:13660"
                 /db_xref="MGI:MGI:1341878"
```

Mascot: http://www.matrixscience.com/

Mascot Search Results

```
User            : Mascot Daemon
Email           : laurent.coquet@univ-rouen.fr
Search title    : Submitted from Martina Rosenberg PC180908 by Mascot Daemon on PBM-IBBR-PERM2
MS data file    : F:\Electrophorese\Martina Rosenberg\LCMSMS\090504_COQUET\MR__1412.mgf
Database        : NCBInr 20090430 (8483808 sequences; 2914572939 residues)
Taxonomy        : Rattus (68467 sequences)
Timestamp       : 11 May 2009 at 12:36:51 GMT
Protein hits    : gi|109482941 PREDICTED: similar to keratin complex 2, basic, gene 6a isoform 1 [Rattus norvegicus]
                  gi|114145409 type II keratin Kb15 [Rattus norvegicus]
                  gi|81891716  RecName: Full=Keratin, type II cytoskeletal 1; AltName: Full=Cytokeratin-1; Short=CK-1; AltName: Full=Keratin-1; Shor
                  gi|47087085  keratin 17 [Rattus norvegicus]
                  gi|14861854  keratin 7 [Mus musculus]
                  gi|57012436  keratin 10 [Rattus norvegicus]
                  gi|56912233  keratin 14 [Rattus norvegicus]
                  gi|6981420   protease, serine, 2 [Rattus norvegicus]
                  gi|56847618  keratin 16 [Rattus norvegicus]
                  gi|57012360  keratin 4 [Rattus norvegicus]
                  gi|109482972 PREDICTED: similar to Keratin, type II cytoskeletal 6G (Cytokeratin-6G) (CK 6G) (K6g keratin) (Keratin-K6irs) (mK6irs
                  gi|57012362  keratin 72 [Rattus norvegicus]
                  gi|57012366  keratin 84 [Rattus norvegicus]
                  gi|42409519  keratin 19 [Rattus norvegicus]
                  gi|109477984 PREDICTED: similar to Growth/differentiation factor 7 precursor (GDF-7) isoform 1 [Rattus norvegicus]
                  gi|18266720  protein phosphatase 4, regulatory subunit 1 [Rattus norvegicus]
                  gi|58865808  chromobox homolog 6 [Rattus norvegicus]
                  gi|109460009 PREDICTED: similar to Salivary gland secretion 1 CG3047-PA [Rattus norvegicus]
                  gi|109466098 PREDICTED: similar to complement component 7 precursor [Rattus norvegicus]
                  gi|109498009 PREDICTED: similar to hemicentin 1 [Rattus norvegicus]
```

Probability Based Mowse Score

Ions score is -10*Log(P), where P is the probability that the observed match is a random event.
Individual ions scores > 43 indicate identity or extensive homology (p<0.05).
Protein scores are derived from ions scores as a non-probabilistic basis for ranking protein hits.

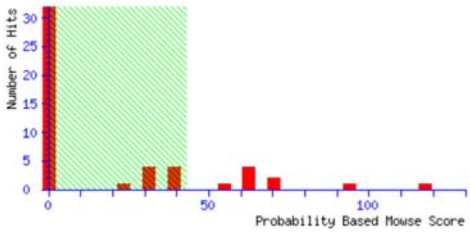

Peptide Summary Report

Format As: Peptide Summary — Help
Significance threshold p< 0.05 — Max. number of hits 20
Standard scoring ○ MudPIT scoring ○ Ions score or expect cut-off 0 — Show sub-sets 0
Show pop-ups ● Suppress pop-ups ○ Sort unassigned Decreasing Score — Require bold red ☐

Select All | Select None | Search Selected | ☐ Error tolerant | Archive Report

1. gi|109482941 **Mass:** 59393 **Score:** 117 **Queries matched:** 4 **emPAI:** 0.18
PREDICTED: similar to keratin complex 2, basic, gene 6a isoform 1 [Rattus norvegicus]
☐ Check to include this hit in error tolerant search or archive report

	Query	Observed	Mr(expt)	Mr(calc)	Delta	Miss	Score	Expect	Rank	Peptide
☑	134	542.2800	1082.5454	1081.5920	0.9534	1	41	0.075	1	K.FASFIDKVR.F
	143	554.5500	1107.0854	1106.5720	0.5135	1	32	0.8	2	K.AQYEEIAKR.S
☑	169	590.3400	1178.6654	1178.5931	0.0723	0	61	0.00084	1	K.YEELQITAGR.H
☑	217	652.3500	1302.6854	1301.7078	0.9776	0	87	1.9e-06	1	R.SLDLDSIIAEVK.A

Proteins matching the same set of peptides:
gi|109482943 **Mass:** 59343 **Score:** 117 **Queries matched:** 4
PREDICTED: similar to keratin complex 2, basic, gene 6a isoform 2 [Rattus norvegicus]
gi|155369696 **Mass:** 59213 **Score:** 117 **Queries matched:** 4
hypothetical protein LOC683313 [Rattus norvegicus]

---

2. gi|114145409 **Mass:** 57574 **Score:** 92 **Queries matched:** 3 **emPAI:** 0.12
type II keratin Kb15 [Rattus norvegicus]
☐ Check to include this hit in error tolerant search or archive report

Query	Observed	Mr(expt)	Mr(calc)	Delta	Miss	Score	Expect	Rank	Peptide
134	542.2800	1082.5454	1081.5920	0.9534	1	41	0.075	1	K.FASFIDKVR.F
217	652.3500	1302.6854	1301.7078	0.9776	0	87	1.9e-06	1	R.SLDLDSIIAEVK.A
285	738.4100	1474.8054	1475.7984	-0.9929	1	34	0.43	2	R.FLEQQNKVLETK.W

Proteins matching the same set of peptides:
gi|149031961 **Mass:** 54499 **Score:** 92 **Queries matched:** 3
rCG50520 [Rattus norvegicus]
gi|149031974 **Mass:** 58060 **Score:** 92 **Queries matched:** 3
type II keratin Kb15 [Rattus norvegicus]
gi|57012352 **Mass:** 62130 **Score:** 92 **Queries matched:** 3
keratin 75 [Rattus norvegicus]

gi|166218811 **Mass:** 58991 **Score:** 92 **Queries matched:** 3
RecName: Full=Keratin, type II cytoskeletal 75; AltName: Full=Cytokeratin-75; Short=CK-75; AltName: Full=Keratin-75; Short=K75; AltName: Full=

3. gi|81891716 **Mass:** 64791 **Score:** 71 **Queries matched:** 5 **emPAI:** 0.16
RecName: Full=Keratin, type II cytoskeletal 1; AltName: Full=Cytokeratin-1; Short=CK-1; AltName: Full=Keratin-1; Short=K1; AltName: Full=Type
Check to include this hit in error tolerant search or archive report

Query	Observed	Mr(expt)	Mr(calc)	Delta	Miss	Score	Expect	Rank	Peptide
134	542.2800	1082.5454	1081.5920	0.9534	1	41	0.075	1	K.FASFIDKVR.F
247	**692.5200**	**1383.0254**	**1383.7034**	**-0.6780**	**1**	**19**	**14**	**2**	**K.SLNDKFASFIDK.V**
252	**697.3300**	**1392.6454**	**1392.7249**	**-0.0794**	**1**	**46**	**0.025**	**1**	**R.TNAENEFVTIKK.D**
84	465.6400	1393.8982	1392.7249	1.1733	1	(34)	0.45	1	R.TNAENEFVTIKK.D
285	738.4100	1474.8054	1474.7780	0.0275	0	58	0.0015	1	R.FLEQQNQVLQTK.W

Proteins matching the same set of peptides:
gi|120474989 **Mass:** 64717 **Score:** 71 **Queries matched:** 5
keratin 1 [Rattus norvegicus]

4. gi|47087085 **Mass:** 48093 **Score:** 70 **Queries matched:** 3 **emPAI:** 0.14
keratin 17 [Rattus norvegicus]
Check to include this hit in error tolerant search or archive report

Query	Observed	Mr(expt)	Mr(calc)	Delta	Miss	Score	Expect	Rank	Peptide
116	**515.4200**	**1028.8254**	**1028.5866**	**0.2389**	**0**	**21**	**9.5**	**1**	**R.VLDELTLAR.A**
127	**532.9700**	**1063.9254**	**1063.6026**	**0.3229**	**1**	**34**	**0.41**	**1**	**R.LASYLDKVR.A**
240	**681.4100**	**1360.8054**	**1360.6834**	**0.1220**	**0**	**64**	**0.00044**	**1**	**R.EVATNSELVQSGK.S**

5. gi|14861854 **Mass:** 50678 **Score:** 66 **Queries matched:** 3 **emPAI:** 0.21
keratin 7 [Mus musculus]
Check to include this hit in error tolerant search or archive report

Query	Observed	Mr(expt)	Mr(calc)	Delta	Miss	Score	Expect	Rank	Peptide
134	542.2800	1082.5454	1081.5920	0.9534	1	41	0.075	1	K.FASFIDKVR.F
142	**552.9000**	**1103.7854**	**1103.5724**	**0.2131**	**0**	**61**	**0.00092**	**1**	**R.SAYGGPVGAGIR.E**
147	**559.4200**	**1116.8254**	**1116.6291**	**0.1963**	**0**	**44**	**0.043**	**1**	**R.IFEAQIAGLR.Q**

Proteins matching the same set of peptides:
gi|149031983 **Mass:** 50775 **Score:** 66 **Queries matched:** 3
keratin complex 2, basic, gene 7, isoform CRA_a [Rattus norvegicus]

6. gi|57012436 **Mass:** 56470 **Score:** 66 **Queries matched:** 3 **emPAI:** 0.12
keratin 10 [Rattus norvegicus]
Check to include this hit in error tolerant search or archive report

Query	Observed	Mr(expt)	Mr(calc)	Delta	Miss	Score	Expect	Rank	Peptide
160	**583.3100**	**1164.6054**	**1164.5775**	**0.0280**	**0**	**49**	**0.012**	**1**	**R.LENEIQTYR.S**
161	**583.3100**	**1164.6054**	**1164.5775**	**0.0280**	**0**	**(46)**	**0.024**	**1**	**R.LENEIQTYR.S**
188	**617.8200**	**1233.6254**	**1233.6717**	**-0.0462**	**1**	**42**	**0.066**	**1**	**R.LKYENEVALR.Q**

7. gi|56912233 **Mass:** 52651 **Score:** 64 **Queries matched:** 3 **emPAI:** 0.06
keratin 14 [Rattus norvegicus]
Check to include this hit in error tolerant search or archive report

Query	Observed	Mr(expt)	Mr(calc)	Delta	Miss	Score	Expect	Rank	Peptide
116	515.4200	1028.8254	1028.5866	0.2389	0	21	9.5	1	R.VLDELTLAR.A
201	**632.8500**	**1263.6854**	**1264.4812**	**-0.7958**	**0**	**8**	**1.5e+02**	**5**	**K.NHEEEMASMR.G + 2 Oxidation (M)**
240	681.4100	1360.8054	1360.6834	0.1220	0	64	0.00044	1	R.EVATNSELVQSGK.S

8. gi|6981420 **Mass:** 25943 **Score:** 61 **Queries matched:** 3 **emPAI:** 0.13
protease, serine, 2 [Rattus norvegicus]
Check to include this hit in error tolerant search or archive report

Query	Observed	Mr(expt)	Mr(calc)	Delta	Miss	Score	Expect	Rank	Peptide
479	**1105.4100**	**2208.8054**	**2210.0967**	**-1.2913**	**0**	**50**	**0.0077**	**1**	**R.LGEHNINVLEGDEQFINAAK.I**
284	**737.4000**	**2209.1782**	**2210.0967**	**-0.9186**	**0**	**(9)**	**1.1e+02**	**3**	**R.LGEHNINVLEGDEQFINAAK.I**
480	**1106.0900**	**2210.1654**	**2210.0967**	**0.0687**	**0**	**(41)**	**0.067**	**1**	**R.LGEHNINVLEGDEQFINAAK.I**

9. gi|56847618 **Mass:** 50746 **Score:** 52 **Queries matched:** 2 **emPAI:** 0.06
keratin 16 [Rattus norvegicus]
Check to include this hit in error tolerant search or archive report

Query	Observed	Mr(expt)	Mr(calc)	Delta	Miss	Score	Expect	Rank	Peptide
211	**644.1100**	**1929.3082**	**1928.8608**	**0.4474**	**1**	**19**	**14**	**1**	**R.ILNEMRDQYEQMAEK.N + 2 Oxidation (M)**
459	**1052.3400**	**2102.6654**	**2102.9539**	**-0.2884**	**0**	**52**	**0.0058**	**1**	**R.GQTGGDVNVEMDAAPGVDLSR.I + Oxidation (M)**

Proteins matching the same set of peptides:
gi|149054207 **Mass:** 35106 **Score:** 52 **Queries matched:** 2
rCG33578 [Rattus norvegicus]
gi|57012432 **Mass:** 52564 **Score:** 52 **Queries matched:** 2
type I keratin KA11 [Rattus norvegicus]

10. gi|57012360 **Mass:** 57631 **Score:** 41 **Queries matched:** 3 **emPAI:** 0.06
keratin 4 [Rattus norvegicus]
Check to include this hit in error tolerant search or archive report

Query	Observed	Mr(expt)	Mr(calc)	Delta	Miss	Score	Expect	Rank	Peptide

	Query	Observed	Mr(expt)	Mr(calc)	Delta	Miss	Score	Expect	Rank	Peptide
	134	542.2800	1082.5454	1081.5920	0.9534	1	41	0.075	1	K.FASFIDKVR.F
☑	143	554.5500	1107.0854	1106.5720	0.5135	1	34	0.48	1	R.AQYEEIARK.S
	285	738.4100	1474.8054	1475.7984	-0.9929	1	34	0.43	2	R.FLEQQNKVLETK.W

---

11. gi|109482972 **Mass:** 57400 **Score:** 41 **Queries matched:** 3 **emPAI:** 0.06
PREDICTED: similar to Keratin, type II cytoskeletal 6G (Cytokeratin-6G) (CK 6G) (K6g keratin) (Keratin-K6irs) (mK6irs1/Krt2-6g) [Rattus norveg
☐ Check to include this hit in error tolerant search or archive report

Query	Observed	Mr(expt)	Mr(calc)	Delta	Miss	Score	Expect	Rank	Peptide
134	542.2800	1082.5454	1081.5920	0.9534	1	41	0.075	1	K.FASFIDKVR.F
27	**388.2700**	**1161.7882**	**1162.5652**	**-0.7770**	**1**	**7**	**2.6e+02**	**8**	**R.KLLESEECR.M + Carbamidomethyl (C)**
285	738.4100	1474.8054	1475.7620	-0.9565	0	34	0.43	2	R.FLEQQNQVLETK.W

---

12. gi|57012362 **Mass:** 60398 **Score:** 41 **Queries matched:** 3 **emPAI:** 0.05
keratin 72 [Rattus norvegicus]
☐ Check to include this hit in error tolerant search or archive report

Query	Observed	Mr(expt)	Mr(calc)	Delta	Miss	Score	Expect	Rank	Peptide
134	542.2800	1082.5454	1081.5920	0.9534	1	41	0.075	1	K.FASFIDKVR.F
285	738.4100	1474.8054	1475.7620	-0.9565	0	34	0.43	2	R.FLEQQNQVLETK.W
292	**746.8800**	**2237.6182**	**2239.0804**	**-1.4622**	**1**	**5**	**3.1e+02**	**7**	**R.LGFSGCSAVISGRFSGSHASVR.A + Carboxymethyl (C)**

Proteins matching the same set of peptides:
gi|166218810 **Mass:** 56816 **Score:** 41 **Queries matched:** 3
RecName: Full=Keratin, type II cytoskeletal 72; AltName: Full=Cytokeratin-72; Short=CK-72; AltName: Full=Keratin-72; Short=K72; AltName: Full=

---

13. gi|57012366 **Mass:** 61163 **Score:** 41 **Queries matched:** 2 **emPAI:** 0.05
keratin 84 [Rattus norvegicus]
☐ Check to include this hit in error tolerant search or archive report

Query	Observed	Mr(expt)	Mr(calc)	Delta	Miss	Score	Expect	Rank	Peptide
134	542.2800	1082.5454	1081.5920	0.9534	1	41	0.075	1	K.FASFIDKVR.F
220	**657.1500**	**1312.2854**	**1312.7649**	**-0.4795**	**1**	**8**	**1.9e+02**	**8**	**R.AISVPRLCVLSR.G**

---

14. gi|42409519 **Mass:** 44609 **Score:** 34 **Queries matched:** 3 **emPAI:** 0.07
keratin 19 [Rattus norvegicus]
☐ Check to include this hit in error tolerant search or archive report

	Query	Observed	Mr(expt)	Mr(calc)	Delta	Miss	Score	Expect	Rank	Peptide
	116	515.4200	1028.8254	1028.5866	0.2389	0	21	9.5	1	R.VLDELTLAR.T
	127	532.9700	1063.9254	1063.6026	0.3229	1	34	0.41	1	R.LASYLDKVR.A
☑	216	**651.3500**	**1300.6854**	**1299.6670**	**1.0184**	**0**	**28**	**1.6**	**1**	**R.ALEQANGELEVK.I**

Proteins matching the same set of peptides:
gi|56788780 **Mass:** 46670 **Score:** 34 **Queries matched:** 3
Krt19 protein [Rattus norvegicus]
gi|149054201 **Mass:** 32406 **Score:** 34 **Queries matched:** 3
keratin complex 1, acidic, gene 19, isoform CRA_b [Rattus norvegicus]
gi|149054202 **Mass:** 25547 **Score:** 34 **Queries matched:** 3
keratin complex 1, acidic, gene 19, isoform CRA_c [Rattus norvegicus]

---

15. gi|109477984 **Mass:** 47558 **Score:** 34 **Queries matched:** 1 **emPAI:** 0.07
PREDICTED: similar to Growth/differentiation factor 7 precursor (GDF-7) isoform 1 [Rattus norvegicus]
☐ Check to include this hit in error tolerant search or archive report

	Query	Observed	Mr(expt)	Mr(calc)	Delta	Miss	Score	Expect	Rank	Peptide
☑	304	**762.7000**	**2285.0782**	**2285.0606**	**0.0175**	**1**	**34**	**0.42**	**1**	**R.TALAGTRGAQGSGGGGGGGGGGGGGGGGAGR.G**

Proteins matching the same set of peptides:
gi|109477986 **Mass:** 46845 **Score:** 34 **Queries matched:** 1
PREDICTED: similar to growth differentiation factor 7 isoform 2 [Rattus norvegicus]

---

16. gi|18266720 **Mass:** 105548 **Score:** 32 **Queries matched:** 2 **emPAI:** 0.03
protein phosphatase 4, regulatory subunit 1 [Rattus norvegicus]
☐ Check to include this hit in error tolerant search or archive report

	Query	Observed	Mr(expt)	Mr(calc)	Delta	Miss	Score	Expect	Rank	Peptide
	211	644.1100	1929.3082	1928.8188	0.4894	1	14	50	5	R.LILPRFCEMCCDCR.M + 4 Carbamidomethyl (C)
☑	262	**712.4500**	**2134.3282**	**2135.8930**	**-1.5649**	**1**	**32**	**0.61**	**1**	**R.KACAECFMAVSCATCQEIR.R + Carbamidomethyl (C); Oxidation (M)**

Proteins matching the same set of peptides:
gi|149037380 **Mass:** 104155 **Score:** 32 **Queries matched:** 2
protein phosphatase 4, regulatory subunit 1, isoform CRA_c [Rattus norvegicus]
gi|149037381 **Mass:** 100931 **Score:** 32 **Queries matched:** 2
protein phosphatase 4, regulatory subunit 1, isoform CRA_d [Rattus norvegicus]

---

17. gi|58865808 **Mass:** 42488 **Score:** 29 **Queries matched:** 1 **emPAI:** 0.08
chromobox homolog 6 [Rattus norvegicus]
☐ Check to include this hit in error tolerant search or archive report

	Query	Observed	Mr(expt)	Mr(calc)	Delta	Miss	Score	Expect	Rank	Peptide
☑	359	**857.7000**	**2570.0782**	**2570.3719**	**-0.2938**	**1**	**29**	**1.3**	**1**	**R.HMKFGTFALYKPPPAPLVPSTAGK.A + Oxidation (M)**

Proteins matching the same set of peptides:
gi|149065905 **Mass:** 44437 **Score:** 29 **Queries matched:** 1
rCG59919 [Rattus norvegicus]

---

18. gi|109460009 **Mass:** 32605 **Score:** 26 **Queries matched:** 1 **emPAI:** 0.10
PREDICTED: similar to Salivary gland secretion 1 CG3047-PA [Rattus norvegicus]
Check to include this hit in error tolerant search or archive report

Query	Observed	Mr(expt)	Mr(calc)	Delta	Miss	Score	Expect	Rank	Peptide
291	744.8400	2231.4982	2231.1178	0.3804	1	26	2.4	1	-.MSEAGGGPGLKAIVETDMPIVK.G + 2 Oxidation (M)

---

19. gi|109466098 **Score:** 0 **Queries matched:** 4
PREDICTED: similar to complement component 7 precursor [Rattus norvegicus]
Check to include this hit in error tolerant search or archive report

Query	Observed	Mr(expt)	Mr(calc)	Delta	Miss	Score	Expect	Rank	Peptide
86	466.7100	1397.1082	1397.5486	-0.4404	1	9	1.7e+02	4	K.CDAQNSKCVCR.E + 2 Carbamidomethyl (C); Carboxymethyl (C)
244	690.2300	2067.6682	2066.0951	1.5730	1	23	5	2	R.ILPLTICKMHVLQCQGR.N + 2 Carbamidomethyl (C)
273	718.3800	2152.1182	2150.8563	1.2618	1	10	91	5	K.CVCKMPYECGSSLDVCAR.D + 2 Carbamidomethyl (C); Carboxymethyl (C); Oxi
324	795.5600	2383.6582	2382.0865	1.5717	1	18	18	2	R.ICVEVNGKEETMSECEAGILR.C + Carbamidomethyl (C); Oxidation (M)

---

20. gi|109498009 **Mass:** 611324 **Score:** 0 **Queries matched:** 6
PREDICTED: similar to hemicentin 1 [Rattus norvegicus]
Check to include this hit in error tolerant search or archive report

Query	Observed	Mr(expt)	Mr(calc)	Delta	Miss	Score	Expect	Rank	Peptide
27	388.2700	1161.7882	1162.5329	-0.7447	0	10	1.1e+02	2	K.EPEAGMWTVK.T + Oxidation (M)
125	532.4000	1594.1782	1593.8475	0.3307	1	13	56	2	R.VRVTTDGSTLNIYR.A
221	657.3100	1968.9082	1967.8969	1.0113	0	8	1.5e+02	4	R.ELYVQGGGDCPEMSVGAIK.I + Oxidation (M)
334	814.0200	2439.0382	2438.1868	0.8513	1	12	62	1	R.MLRLMQTRPEDAGQYTCIVR.N + Carboxymethyl (C)
355	851.6600	2551.9582	2553.1561	-1.1979	1	6	2.5e+02	8	R.SRLCNNPPPANGGRPCQGSESEAR.Q + Carbamidomethyl (C)
466	1068.4800	3202.4182	3202.3689	0.0493	1	10	75	4	R.TRLCNSPPPSFGGAYCNGAETQMQVCNER.H + Carbamidomethyl (C); Oxidation (M

Proteins matching the same set of peptides:
gi|109498802 **Mass:** 611184 **Score:** 0 **Queries matched:** 6
PREDICTED: similar to High Incidence of Males (increased X chromosome loss) family member (him-4) [Rattus norvegicus]

Peptide matches not assigned to protein hits: (no details means no match)

Query	Observed	Mr(expt)	Mr(calc)	Delta	Miss	Score	Expect	Rank	Peptide
57	429.8400	857.6654	856.5130	1.1524	1	29	1.3	1	ASGAAKKPK
300	755.2100	2262.6082	2264.1331	-1.5250	1	28	1.5	1	QQEEVKAPQMSQERPPQVR
247	692.5200	1383.0254	1381.7129	1.3125	0	27	2.2	1	AELEFLASIFDK
7	320.4500	638.8854	639.2799	-0.3944	0	26	2.4	1	CFSQR
3	309.0400	924.0982	925.5960	-1.4979	0	26	3.3	1	LLAGLLAGAK
56	429.5400	1285.5982	1285.6561	-0.0579	0	25	3.6	1	CQNALQQVVAR + Carbamidomethyl (C)
343	831.6400	2491.8982	2493.2798	-1.3816	1	25	3.2	1	KTSNGENHLSFSHLCLVGNPVLK
308	767.3900	2299.1482	2299.0137	0.1345	1	25	3	1	TLYMSDMFSTNFGNPESAKK + 2 Oxidation (M)
61	436.1800	870.3454	870.5650	-0.2196	1	25	3.4	1	KASLALIR
409	954.7200	2861.1382	2861.3115	-0.1733	1	24	3.6	1	VMGCPVCSASHPCSSTAKRPSMLLPR + Carbamidomethyl (C); Carboxymethyl (C);
244	690.2300	2067.6682	2068.0484	-0.3802	1	24	4.3	1	LGGPGGTAGGTGGGRVLECPSIR
81	459.4200	1375.2382	1373.6926	1.5456	0	23	5.9	1	LDVDSILDELDK
42	416.4500	830.8854	830.4498	0.4357	0	23	6.2	1	ASGQLIDK
196	625.4200	1873.2382	1872.9047	0.3335	0	23	5	1	WQFSSDGADIGFGVFLK
74	447.3000	1338.8782	1339.6554	-0.7772	1	23	5.4	1	VEEAMFSSLRR + Oxidation (M)
70	445.2400	1332.6982	1331.5551	1.1431	0	23	5.8	1	ACTSLYSEEDSK
429	993.6400	2977.8982	2977.5075	0.3906	0	23	4.6	1	LACCSLDGSISLCQLVPAPPTVLHVLR + Carbamidomethyl (C); 2 Carboxymethyl (C
179	602.4500	1804.3282	1804.8890	-0.5608	1	23	6.2	1	ERADIEGLMARPEYR
46	421.4700	1261.3882	1261.5431	-0.1549	0	23	6.4	1	DLELSDMHMR + Oxidation (M)
99	491.8400	1472.4982	1473.7286	-1.2304	0	22	5.9	1	GASWMPVTGNTLPK + Oxidation (M)
33	404.5200	807.0254	806.4286	0.5968	0	22	6.9	1	ELSGVFR
95	488.3900	974.7654	974.5073	0.2582	0	22	7.7	1	EFNLLDPK
69	444.5500	1330.6282	1331.5962	-0.9680	1	22	8.6	1	GQTKSSCYISCR
219	656.5400	1966.5982	1967.9054	-1.3072	1	21	8.1	1	DACPAARPTGPCGDAAPRSR
102	495.0300	1482.0682	1483.6622	-1.5940	0	21	9.8	1	MFGSIPMQACQQK + Oxidation (M)
58	431.9500	1292.8282	1292.7088	0.1193	1	21	10	1	RDTGQYVLTLK
53	427.4700	1279.3882	1280.5377	-1.1495	0	21	8.7	1	SSADCTSLVPQCA
281	733.4100	2197.2082	2195.9432	1.2650	0	21	7.2	1	LQLASASHLCSLTCQCCSGR + 2 Carboxymethyl (C)
178	598.1700	1791.4882	1790.9163	0.5719	0	21	8.3	1	DATAGATPTPPAPPVSSVR
138	547.3500	1092.6854	1091.4964	1.1890	0	21	8	1	RPSNSGLCCR
163	584.7600	1751.2582	1749.8178	1.4404	1	21	8.7	1	ISRIPGAPCELCGYR + 2 Carboxymethyl (C)
385	907.6500	2719.9282	2719.4838	0.4443	1	21	7.6	1	REVCLLSGLVFAAGLCAILAAMLALK + Carboxymethyl (C); Oxidation (M)
34	404.7800	807.5454	806.3658	1.1797	0	21	8.6	1	DLSSEEK
30	399.4800	1195.4182	1196.5972	-1.1790	0	21	8.6	1	HMEAAALLGER
320	790.2400	2367.6982	2368.1127	-0.4145	0	21	8.2	1	SQGFLCDCTVMVGSIQFLAHR + Carbamidomethyl (C)
12	344.2800	1029.8182	1030.5018	-0.6836	0	21	10	1	WQAPASCLR
460	1053.2600	2104.5054	2104.0189	0.4866	1	20	8.1	1	METIMLSPHLCLKMAQGK + Carboxymethyl (C); Oxidation (M)
230	669.3400	2004.9982	2003.8863	1.1119	1	20	9	1	MLAGIDDCYTSARGCTATR
5	317.0500	632.0854	633.3333	-1.2479	0	20	13	1	LTSDAK
491	1143.9600	3428.8582	3427.7131	1.1451	0	20	7.1	1	VTCFMLHHIEEPCSLGAHAAVIVPPTWIIK + 2 Carboxymethyl (C)
91	479.2100	1434.6082	1433.7739	0.8342	1	20	10	1	VHTTKTSGHPTLR
233	670.7800	1339.5454	1339.6772	-0.1318	0	20	10	1	YEAFISDVLQR
253	700.6900	2099.0482	2099.9477	-0.8995	1	20	10	1	NAKTGSNGEKPHQCQVCGK + Carbamidomethyl (C); Carboxymethyl (C)
327	801.6100	1601.2054	1601.8698	-0.6644	0	20	11	1	ALLQAILQTEDMLK + Oxidation (M)
75	448.7700	1343.2882	1344.4679	-1.1797	1	20	11	1	MCGECEACRR + 2 Carbamidomethyl (C); Carboxymethyl (C); Oxidation (M)
111	507.0200	1518.0382	1518.6378	-0.5996	0	20	12	1	HSAVMLSCSCSGHK + Carbamidomethyl (C); Oxidation (M)
225	665.4900	1993.4482	1993.8218	-0.3736	1	20	12	1	DESNPCGNGGDRAGATCVSK + Carbamidomethyl (C)
68	444.1800	1329.5182	1329.7405	-0.2223	0	20	13	1	LRPGFGGAVTEVK
369	876.4500	2626.3282	2627.2440	-0.9159	1	19	9.8	1	VCAQNCAPKQCKPMAGDAILPSPR + 2 Carbamidomethyl (C); Oxidation (M)
410	957.4600	2869.3582	2869.2366	0.1216	1	19	9.1	1	MPEDKLLFCPSGSCPHECLNGAFCSK + Carbamidomethyl (C)
324	795.5600	2383.6582	2385.1167	-1.4585	1	19	12	1	LIDDMVAQAMKSEGGFIWACK + Carbamidomethyl (C); Oxidation (M)
121	525.0700	1572.1882	1570.7912	1.3969	1	19	15	1	IQLSTYLMKSEDK + Oxidation (M)
35	405.6500	1213.9282	1212.6727	1.2554	0	19	13	1	THVPHLSLGPR
21	371.2200	1110.6382	1111.6125	-0.9743	0	19	11	1	ETTDLVPIPK
40	415.6400	1243.8982	1243.5941	0.3041	0	19	14	1	MSLLGGYCISK + Oxidation (M)
403	935.6400	2803.8982	2804.1285	-0.2304	0	19	12	1	MCSTVTCMDTSSPGPPASPTSPSEYR + 2 Carboxymethyl (C)
101	494.2400	986.4654	987.5349	-1.0694	0	19	13	1	QNSLATINK
301	758.0100	2271.0082	2270.2118	0.7964	1	19	11	1	SVEDLTSGPVGLTSIEERILR
139	550.2900	1647.8482	1648.8461	-0.9979	1	19	13	1	FSKPLYLKSHDEVS

☑	317	786.6900	2357.0482	2357.2334	-0.1853	0	19	13	1	QALQSLTELVMAKPTCVPVQK + Carboxymethyl (C); Oxidation (M)
☑	203	635.1300	1268.2454	1268.5377	-0.2922	1	19	15	1	LDKCSEAESCK + Carbamidomethyl (C)
☑	182	610.2700	1827.7882	1826.9389	0.8493	1	19	14	1	KWMYLESIFIGGDIR
☑	197	626.2400	1875.6982	1874.7597	0.9385	1	18	14	1	MGCPGEEGYGRASCISK + Carbamidomethyl (C); Carboxymethyl (C); Oxidation
☑	458	1048.6500	3142.9282	3142.2830	0.6451	1	18	12	1	ACVVRFWAEEDGPFPCPECADDCWQR + 2 Carbamidomethyl (C)
☑	159	581.8600	1161.7054	1161.4617	0.2438	0	18	16	1	TNGMCSVCYK + Carbamidomethyl (C)
☑	132	537.5000	1072.9854	1073.5406	-0.5552	0	18	18	1	SAWLAAWGGR
☑	16	356.5800	711.1454	712.5211	-1.3756	1	18	14	1	KVLLLK
☑	60	433.6700	1297.9882	1299.4947	-1.5065	0	18	16	1	CNWTCFSMHR + Oxidation (M)
☑	314	781.7000	2342.0782	2341.1808	0.8973	1	18	15	1	HAQVISSSGIMSLGIGDRNIDR + Oxidation (M)
☑	475	1097.1400	3288.3982	3288.8356	-0.4374	1	18	12	1	VRTLNTCVSHILAVLVFWIPVIGVSMIHR + Oxidation (M)
☑	311	772.2200	2313.6382	2314.1814	-0.5432	0	18	16	1	AAMVGGSFVACAAALTFSASVVIR + Oxidation (M)
☑	326	800.0700	2397.1882	2397.0658	0.1224	1	18	14	1	CCQAGCGSVCSKPNGLSEGKLSR + 2 Carbamidomethyl (C)
☑	277	729.3300	2184.9682	2184.0885	0.8797	0	18	15	1	LSTLSSPSAILWAPDSPPCSR
☑	305	763.5400	2287.5982	2287.0725	0.5256	1	18	16	1	KAADMGNPVGQSGLGMAYLYGR + 2 Oxidation (M)
☑	156	577.6600	1729.9582	1728.8254	1.1328	1	18	16	1	THWTLDKCGVQADAK + Carbamidomethyl (C)
☑	144	554.6100	1107.2054	1108.5005	-1.2950	1	18	17	1	MKQLQECR + Carboxymethyl (C); Oxidation (M)
☑	136	547.2500	1638.7282	1637.9213	0.8069	1	18	17	1	AKLSRPQRPVEETK
☑	51	426.2100	1275.6082	1274.6805	0.9276	1	18	18	1	RVLISSLGMHF + Oxidation (M)
☑	222	662.2900	1983.8482	1983.0248	0.8234	1	18	17	1	AAMPPQIIQFPEDQKVR + Oxidation (M)
☑	468	1071.0800	2140.1454	2140.0809	0.0645	0	18	14	1	GVVGGVMIVTPNNIMFDPHK + Oxidation (M)
☑	331	806.5800	2416.7182	2417.0169	-0.2987	0	18	17	1	LFNMCHVCHVCQMICTFLK + 2 Carboxymethyl (C); 2 Oxidation (M)
☑	153	573.8900	1718.6482	1719.9164	-1.2682	1	18	19	1	MMAGAFILGGLADKLGR
☑	431	995.3100	2982.9082	2982.3310	0.5772	1	18	16	1	MNSLQTEDTAMYFCARSQLPGYNLR + Carboxymethyl (C); Oxidation (M)
☑	181	610.2100	1218.4054	1219.6165	-1.2111	1	17	19	1	AQVSKLGCCVR + Carbamidomethyl (C)
☑	408	948.1700	1894.3254	1894.9385	-0.6130	0	17	16	1	GLDPVTELDSSKPHSGTR
☑	443	1008.2900	3021.8482	3021.7949	0.0533	1	17	16	1	FWFHWGLLLLLLGAPLRGLALPHIR
☑	15	354.8400	1061.4982	1060.5560	0.9422	1	17	21	1	RTMTRPSGR
☑	298	753.8300	2258.4682	2258.1154	0.3528	0	17	18	1	NYLPAINGIVFLVDCADHER
☑	437	1001.0800	3000.2182	2999.4407	0.7775	0	17	15	1	MPSSAHLQDPPPHLSGTLTQDEGQTSLR
☑	322	793.0100	2376.0082	2376.9193	-0.9111	1	17	17	1	SRSCGLCDLYYPCCLCDYK + Carbamidomethyl (C); 2 Carboxymethyl (C)
☑	110	506.8000	1517.3782	1518.6377	-1.2596	1	17	20	1	MVQSCSAYGCKNR + Carbamidomethyl (C); Oxidation (M)
☑	243	689.9500	2066.8282	2066.9248	-0.0966	1	17	19	1	TAEGQEMLNQMEELREK + 2 Oxidation (M)
☑	28	389.6800	1166.0182	1167.5740	-1.5558	1	17	26	1	SITCMTTGKAR
☑	128	533.2900	1596.8482	1595.6920	1.1562	1	17	20	1	SLTMCGSVDKDPNR + Carboxymethyl (C); Oxidation (M)
☑	383	902.7100	2705.1082	2704.1567	0.9514	1	17	19	1	VNGDGCSLFCKQEVSFNCIDEPSR + Carboxymethyl (C)
☑	273	718.3800	2152.1182	2153.0173	-0.8991	1	17	18	1	YIFSDKTGTLTCNVMQFK + Carboxymethyl (C)
☑	17	357.6300	1069.8682	1068.4514	1.4167	1	17	21	1	GKCCGLCTAR + Carboxymethyl (C)
☑	261	711.4700	2131.3882	2129.9787	1.4095	0	17	20	1	DESSPPQLGQDCLILNSEK + Carboxymethyl (C)
☑	413	960.1400	2877.3982	2876.1691	1.2290	1	17	17	1	MYMVMEYCVCGMQEMLDSVPEKR + Carbamidomethyl (C); 3 Oxidation (M)
☑	328	802.6500	2404.9282	2406.1712	-1.2430	1	17	22	1	MYLLFSSSLKNCIQPVNGYR + Carboxymethyl (C); Oxidation (M)
☑	133	542.2300	1082.4454	1082.4558	-0.0104	0	17	21	1	ALLMCEACR + Carboxymethyl (C); Oxidation (M)
☑	129	534.8300	1067.6454	1067.4818	0.1637	1	17	21	1	NLGREYCR + Carboxymethyl (C)
☑	362	860.2800	2577.8182	2579.2070	-1.3888	1	17	20	1	NFVSGIKLACTSCTFATSVGDAMAK + Carboxymethyl (C)
☑	396	930.2500	1858.4854	1859.9928	-1.5073	0	17	20	1	IPEDVVTGLPLCHAALR + Carbamidomethyl (C)
☑	266	715.5200	2143.5382	2143.9206	-0.3824	1	17	22	1	VCCAVGSCVRSACAAAWAMGR + Carbamidomethyl (C); Oxidation (M)
☑	286	738.4900	2212.4482	2210.9996	1.4485	1	17	22	1	VVVMVMVMKEEEEEEEEK + Oxidation (M)
☑	453	1037.0200	3108.0382	3107.4045	0.6337	1	17	17	1	QRFPEIPEGVVSQCMLQNNNNLEACCR + Oxidation (M)
☑	198	626.9000	1877.6782	1877.8789	-0.2008	1	17	21	1	VSSGTPTMNGGKVVNQDST
☑	119	520.3300	1038.6454	1039.6390	-0.9935	0	17	21	1	GLAGIDLILR
☑	363	860.3800	2578.1182	2579.1133	-0.9951	1	17	19	1	MCGPPELRECEPLSMLSCVCPR + 2 Carbamidomethyl (C); Oxidation (M)
☑	50	425.4200	1273.2382	1274.5457	-1.3076	1	17	30	1	FMKGEMTMER + Oxidation (M)
☑	148	564.2100	1126.4054	1126.5917	-0.1862	1	17	23	1	MEPSRAPALR
☑	393	925.9100	2774.7082	2773.1638	1.5444	1	16	20	1	CKPCPVAQSAMVCGSDGHTYTSKCK + Carbamidomethyl (C); 2 Carboxymethyl (C)
☑	265	715.2200	2142.6382	2141.9102	0.7280	1	16	22	1	KLNSSEDGTQGCMGLPCVVM + Carboxymethyl (C); Oxidation (M)
☑	366	867.1000	2598.2782	2598.3112	-0.0330	0	16	21	1	DSWKPLTAGVQVCQALESPGGSQIK
☑	376	891.7500	2672.2282	2672.1348	0.0934	1	16	21	1	MRHDNTCPVLMALCVMCDIYR + Carbamidomethyl (C); 2 Carboxymethyl (C); Oxi
☑	65	442.5200	883.0254	882.4494	0.5761	0	16	25	1	AICNHLR + Carbamidomethyl (C)
☑	166	586.3000	1170.5854	1171.6673	-1.0818	1	16	23	1	ALTRAVEASVR
☑	208	643.2200	1926.6382	1927.0601	-0.4219	1	16	23	1	VMEGIHKAIELGYKPVK + Oxidation (M)
☑	43	418.1700	1251.4882	1252.7251	-1.2370	1	16	27	1	RQGNLTLPLNK
☑	418	968.6500	2902.9282	2901.4001	1.5281	0	16	22	1	WLTVEMMQDGHQVSLLSGELTVEQR + Oxidation (M)
☑	117	516.3600	1546.0582	1546.7344	-0.6763	1	16	27	1	MGKSQLCSAGTVHGR + Oxidation (M)
☑	97	489.5800	1465.7182	1466.7850	-1.0668	1	16	27	1	MSRVPCGPLAPLAR
☑	104	498.6700	1492.9882	1493.6068	-0.6186	0	16	24	1	ESYGPPMHPFCR + Carboxymethyl (C); Oxidation (M)
☑	420	969.1100	2904.3082	2903.3061	1.0021	1	16	20	1	DLLSDRCVLSCPSMDLVTCLLDFR + 3 Carboxymethyl (C); Oxidation (M)
☑	213	648.0400	1294.0654	1292.4892	1.5763	0	16	26	1	GEEEEGEEEEK
☑	232	670.7800	1339.5454	1339.7347	-0.1893	0	16	25	1	ELTLSANPGITPK
☑	14	346.5300	1036.5682	1037.5692	-1.0010	1	16	29	1	RPKMSIYK + Oxidation (M)
☑	500	1281.9500	3842.8282	3842.5297	0.2985	1	16	17	1	YEVCDIPQCSEVECMTCNGESYRGLMDHTESGK + 2 Carbamidomethyl (C); Oxidation
☑	157	578.4100	1732.2082	1732.6491	-0.4409	0	16	27	1	DCDLLQDTCYNCGR + Carbamidomethyl (C); Carboxymethyl (C)
☑	371	880.5300	2638.5682	2639.4183	-0.8501	1	16	22	1	SLEPQALLGLQNLYYLHLERNR
☑	172	592.3800	1774.1182	1773.8502	0.2680	1	16	26	1	RDMLAVNASHPMTTSK + Oxidation (M)
☑	171	591.2700	1770.7882	1770.6912	0.0969	0	16	26	1	CPNPTCENMNFSWR + Carbamidomethyl (C); Oxidation (M)
☑	141	552.1500	1653.4282	1654.7443	-1.3161	1	16	29	1	FTPCSKNCEATQIR + Carboxymethyl (C)
☑	303	761.2800	1520.5454	1520.6963	-0.1509	1	16	24	1	PCDTMKVGGNLDSK + Carbamidomethyl (C)
☑	6	318.7200	953.1382	953.5406	-0.4024	1	16	28	1	AARAPELAR
☑	13	346.4800	1036.4182	1036.5223	-0.1041	1	16	34	1	MASEKASVAK + Oxidation (M)
☑	257	705.9300	1409.8454	1410.6119	-0.7665	0	16	25	1	VCDCVSANSSELK + Carbamidomethyl (C)
☑	372	886.4400	2656.2982	2655.4233	0.8748	1	16	24	1	LLAGFCKLLLYGVLELDAASDVFK + Carboxymethyl (C)
☑	47	422.1900	1263.5482	1262.6289	0.9193	1	16	28	1	ANKVTGTAGMADK
☑	333	811.5400	2431.5982	2430.1447	1.4535	0	16	26	1	MSILGIIFIQGNSTSEEDMWK + 2 Oxidation (M)
☑	124	528.4500	1582.3282	1580.7947	1.5335	1	16	31	1	HEFPELAPRETQK
☑	44	419.4700	1255.3882	1256.5893	-1.2011	1	16	33	1	MEKSISQCFK + Carbamidomethyl (C)
☑	164	585.4200	1753.2382	1752.7633	0.4749	1	16	29	1	CHTGKKPYECLECGK + Carboxymethyl (C)
☑	218	654.5000	1960.4782	1958.9515	1.5267	1	16	30	1	MEEVPAQIPMMKSPLDK + Oxidation (M)
☑	130	535.0600	1602.1582	1602.8213	-0.6631	1	16	32	1	SIRITATDAEDQGVK
☑	192	622.7800	1865.3182	1863.9737	1.3445	1	16	30	1	QLAQNNQLCVALKHER
☑	339	819.8900	1637.7654	1638.7130	-0.9476	0	16	27	1	NHLEEHMDTCLPK + Carbamidomethyl (C); Oxidation (M)
☑	52	427.4500	1279.3282	1278.7071	0.6211	0	16	31	1	ITYEPITTTLK
☑	337	815.1100	2442.3082	2441.1362	1.1720	1	16	27	1	TERPGAGCAGLSGCAPAPRSPCGLR + Carboxymethyl (C)
☑	425	988.0200	2961.0382	2962.3992	-1.3610	1	16	22	1	RTAIPSWSANSSVAGSTSSSLCEHWVSR
☑	20	363.8900	725.7654	725.3860	0.3794	0	16	29	1	FAYLGR
☑	41	415.9100	829.8054	830.4498	-0.6443	0	16	34	1	AAQVLGVSS
☑	241	683.5800	1365.1454	1363.7684	1.3770	1	15	31	1	IQALRASGAGAPPR
☑	335	814.1200	2439.3382	2439.9965	-0.6583	0	15	29	1	SGDYYLFETDSEEEEEEELK
☑	373	887.6000	1773.1854	1773.9738	-0.7883	1	15	28	1	DIIVLQRGGTGFAGSGVK
☑	414	962.6500	2884.9282	2884.4173	0.5109	1	15	27	1	AVICRCTTYDGIDGLPLPSMLQDFLK + Oxidation (M)
☑	463	1059.1100	3174.3082	3173.3603	0.9479	1	15	23	1	MGFGPDPTFSSCVPDPPMISEEKGPCYR + 2 Carbamidomethyl (C); Oxidation (M)
☑	120	523.5000	1044.9854	1044.5788	0.4066	1	15	37	1	SISRPRSSR
☑	195	624.2700	1869.7882	1868.9342	0.8540	1	15	30	1	LKGFDVFNALDLMENK + Oxidation (M)
☑	11	343.8300	1028.4682	1027.4328	1.0354	0	15	33	1	MGHAHSGMGK + Oxidation (M)
☑	282	733.9300	2198.7682	2199.9954	-1.2272	0	15	29	1	MEGESVKPSPQPTEQAGDGEK
☑	455	1040.2700	3117.7882	3116.6024	1.1858	1	15	28	1	SPATMSTAPKSAPQTRPSSPATVTTVTTVTK + Oxidation (M)
☑	426	989.3900	1976.7654	1977.0387	-0.2733	1	15	25	1	DMAAVQRTLMALGSVAVTK + Oxidation (M)
☑	72	445.5500	1333.6282	1332.6279	1.0003	1	15	39	1	VCGDTCVPAAGKR + Carbamidomethyl (C)
☑	416	967.2200	2898.6382	2899.2319	-0.5937	0	15	29	1	ATCCMPGVGYTIIPAQCCEDQGAATPK + 2 Carbamidomethyl (C); Carboxymethyl (C
☑	77	452.1300	1353.3682	1352.6871	0.6811	1	15	36	1	MPGERGAAGLPGPK + Oxidation (M)
☑	149	564.3500	1690.0282	1690.7596	-0.7314	1	15	33	1	YCYICHSLQYKR + Carbamidomethyl (C); Carboxymethyl (C)
☑	109	506.5800	1011.1454	1012.5162	-1.3708	1	15	34	1	AGTTTHNRR
☑	345	834.2300	2499.6682	2501.0639	-1.3957	1	15	32	1	KAGCPCVCVCVCVCVCVHTYK + 5 Carbamidomethyl (C)

☑	39	413.2100	1236.6082	1237.6462	-1.0380	1	15	32	1	VHAARCGLQGAR
☑	210	643.7000	1285.3854	1286.7459	-1.3604	1	15	32	1	ALARGLSPAYLR
☑	73	446.3900	1336.1482	1337.7278	-1.5796	0	15	43	1	YMAIIHPLQPR
☑	38	413.1300	1236.3682	1236.6826	-0.3145	0	15	33	1	EPQLQAVTVPR
☑	190	621.2700	1860.7882	1862.1353	-1.3471	1	15	32	1	ARPLVPSSQKALLLELK
☑	467	1069.1400	3204.3982	3204.7517	-0.3535	1	15	26	1	TMVYGVAAGAFVLLVFIVSMIYLACKKPK + Carbamidomethyl (C); Oxidation (M)
☑	187	617.7400	1850.1982	1850.9198	-0.7216	0	15	35	1	LVPYLSFMTYCLISK + Carboxymethyl (C); Oxidation (M)
☑	54	427.5500	1279.6282	1279.7612	-0.1330	1	15	34	1	ANIRDPLQIIK
☑	268	716.4000	2146.1782	2144.9176	1.2605	1	15	31	1	SCLVKYLEENNTCPTCR + Carbamidomethyl (C); 2 Carboxymethyl (C)
☑	151	566.7200	1697.1382	1696.9559	0.1823	1	15	37	1	MAVPNHTPHLILAKR
☑	32	402.5900	1204.7482	1203.4900	1.2582	0	15	41	1	FSSLQCSSCDK
☑	249	694.3000	1386.5854	1385.7159	0.8695	0	15	32	1	AHIACLGVLSAMK + Carbamidomethyl (C); Oxidation (M)
☑	256	705.4300	2113.2682	2112.0345	1.2336	0	15	33	1	CLEAVILGMYPLSFCAVPL + Carboxymethyl (C); Oxidation (M)
☑	78	457.5500	1369.6282	1368.7799	0.8483	1	15	37	1	VPLEQLCLRIK + Carboxymethyl (C)
☑	395	929.8600	2786.5582	2787.4564	-0.8982	1	15	29	1	MLLLPPRPPHPRSSSPEVMDPPPPK + Oxidation (M)
☑	83	461.9200	1382.7382	1383.6275	-0.8893	0	15	39	1	SPTMAGGLFAMNR + 2 Oxidation (M)
☑	137	547.2600	1638.7582	1639.8603	-1.1021	0	15	36	1	MPILIPSPSGLSAEGR + Oxidation (M)
☑	342	831.3300	2490.9682	2490.3112	0.6570	1	15	31	1	TSRMTVLNTEKPTVTVDIGGTVR + Oxidation (M)
☑	55	429.5200	1285.5382	1286.4730	-0.9348	0	15	43	1	YCHSLNCDMK + Carboxymethyl (C); Oxidation (M)
☑	76	449.3200	1344.9382	1344.5690	0.3692	0	14	37	1	CFIMGADNVSSK + Carboxymethyl (C); Oxidation (M)
☑	427	991.8800	1981.7454	1981.0190	0.7264	1	14	29	1	KCVPEGIEDPQALLEGVK + Carbamidomethyl (C)
☑	177	597.3100	1788.9082	1788.8134	0.0947	0	14	36	1	GLADPGCCAAATAAAAAAEK + Carbamidomethyl (C)
☑	449	1020.0200	3057.0382	3056.4226	0.6156	1	14	30	1	AAASPVCGSQASAACASTPATAPAPAAGLGRCR + Carbamidomethyl (C); Carboxymethy
☑	193	623.3400	1866.9982	1865.7706	1.2275	1	14	37	1	MVVDCCTDPDGRAVDR + Carbamidomethyl (C); Carboxymethyl (C)
☑	357	856.7200	2567.1382	2567.3128	-0.1746	1	14	35	1	VKTSVFLLSWLTALPESSLCGCR + Carboxymethyl (C)
☑	346	836.4500	2506.3282	2506.0650	0.2632	0	14	32	1	AANPIDSMLCHFCHNVSFPCSR + Carboxymethyl (C)
☑	114	512.3700	1534.0882	1533.7634	0.3247	0	14	41	1	AGLSTSPSAIASTSER
☑	375	889.0800	2664.2182	2664.2923	-0.0741	1	14	33	1	VETCMFMIKLPQYSSLEIMLEK + 2 Oxidation (M)
☑	462	1058.3600	2114.7054	2115.9176	-1.2121	1	14	31	1	REDAWACIIEQLCYSCR + Carboxymethyl (C)
☑	434	998.7000	1995.3854	1994.9026	0.4829	1	14	35	1	LPSCGCHPKHSWGTLCR + 2 Carbamidomethyl (C)
☑	236	675.3500	2023.0282	2023.0138	0.0144	1	14	38	1	AAFFGLLGYSLYWMGRR + Oxidation (M)
☑	209	643.6400	1285.2654	1284.6132	0.6522	1	14	39	1	CGAEDHVEAVKK
☑	274	724.0800	1446.1454	1445.7119	0.4335	1	14	41	1	SPAACSGCVLPTKR + Carbamidomethyl (C)
☑	399	932.0400	2793.0982	2792.2303	0.8679	0	14	31	1	DPMVQEQVCSALCSLGEAQPDETLR + Carboxymethyl (C); Oxidation (M)
☑	200	631.7000	1892.0782	1890.8815	1.1967	1	14	38	1	MPINKSEKPESCDNVK + Carbamidomethyl (C); Oxidation (M)
☑	397	930.7700	2789.2882	2788.1900	1.0982	1	14	34	1	CVLSWISWIQSCCSSNRTAPSPMC + 2 Carbamidomethyl (C); Oxidation (M)
☑	89	475.4600	1423.3582	1422.5955	0.7627	1	14	49	1	HRMMSAWATCR + Carboxymethyl (C); Oxidation (M)
☑	18	359.1800	1074.5182	1074.4876	0.0305	1	14	50	1	SRASPCPSGR + Carboxymethyl (C)
☑	176	596.3800	1190.7454	1190.5608	0.1847	0	14	42	1	TEVNSGFFYK
☑	258	708.3400	2121.9982	2121.0061	0.9920	1	14	39	1	MPQLNRSAPFSWTQEASR + Oxidation (M)
☑	404	935.7700	2804.2882	2804.8646	-0.5764	0	14	36	1	VGCCGGCCGGCCGCCGCCRPVVVCCR + 2 Carbamidomethyl (C); 4 Carboxymethyl (
☑	374	887.9700	1773.9254	1774.7945	-0.8690	0	14	37	1	AMVTGGWGGPGGDTNGVDK
☑	206	639.8900	1916.6482	1915.9687	0.6795	0	14	39	1	GRPTIQQCPLPGLEDHR
☑	9	328.5200	982.5382	981.4623	1.0758	1	14	36	1	SMEGMGKVK + Oxidation (M)
☑	390	917.4600	1832.9054	1833.7431	-0.8376	0	14	34	1	MTMTTMADGLEGQDSK + 2 Oxidation (M)
☑	401	934.3400	2799.9982	2800.3571	-0.3589	1	14	35	1	TATRPVRTMWMHQSPCTAVTSAGPAK + Oxidation (M)
☑	415	966.8300	2897.4682	2897.4229	0.0453	1	14	34	1	LDLNMLSDSENPQHLELHEPPKVDK
☑	412	959.0000	2873.9782	2873.0532	0.9250	0	14	35	1	FLCWATGSFCETMCQSVMCCFLTG + 3 Carboxymethyl (C); 2 Oxidation (M)
☑	419	968.7300	1935.4454	1937.0179	-1.5725	0	14	38	1	EADQIPDCEILLLLAPK + Carbamidomethyl (C)
☑	239	679.6900	2036.0482	2037.0419	-0.9937	0	14	42	1	IDVLFLLDSSAGTTPEGFR
☑	23	378.5300	1132.5682	1133.5573	-0.9891	0	14	43	1	MAETCVPVLR + Oxidation (M)
☑	62	437.1300	872.2454	873.4920	-1.2465	0	14	47	1	ISVINGGSK
☑	88	475.4200	1423.2382	1422.6483	0.5899	0	14	52	1	GNSCILADEMGLGK + Oxidation (M)
☑	131	536.5800	1606.7182	1607.8089	-1.0908	1	14	49	1	ELSPEAICHRTAPK + Carbamidomethyl (C)
☑	492	1150.0700	2298.1254	2298.1777	-0.0522	0	14	32	1	MSETAPAASSTLVPAPVEKPATK + Oxidation (M)
☑	432	996.0200	2985.0382	2983.5293	1.5089	1	14	36	1	LSCRLQCALAALCIVLALGGVTGAPSDPR + 2 Carboxymethyl (C)
☑	466	1068.4800	3202.4182	3201.7797	0.6385	1	14	33	1	LLQTVLTAALMFLVYEKLTAATFTVMGLK + Oxidation (M)
☑	146	557.7000	1113.3854	1112.3395	1.0459	0	14	44	1	CHSCCCVVS + Carbamidomethyl (C); 2 Carboxymethyl (C)
☑	80	458.5600	915.1054	915.4848	-0.3793	0	14	53	1	QNLGLMPK + Oxidation (M)
☑	125	532.4000	1594.1782	1592.6849	1.4933	1	14	51	1	QGCASEPKDGSSQSR + Carbamidomethyl (C)
☑	478	1103.3600	3307.0582	3306.7686	0.2896	1	14	37	1	SSHSPGIDKLLALFYTAVTSMLNPIIYSLR
☑	296	752.2700	1502.5254	1503.6914	-1.1659	0	14	41	1	ASSHSSQSQGGGSVTK
☑	451	1022.3000	3063.8782	3062.4525	1.4257	1	13	40	1	SFTTCLLASTQVQWHSVHTCIQACRK + Carbamidomethyl (C); Carboxymethyl (C)
☑	64	442.0900	882.1654	881.5334	0.6320	0	13	48	1	TITHIVAK
☑	201	632.8500	1263.6854	1263.5513	0.1341	1	13	47	1	MNGEAEDRAGSK
☑	439	1004.0600	3009.1582	3008.3487	0.8095	0	13	37	1	MLEIQCIVNVCLEIMEPGGNVGEEDDK + 2 Oxidation (M)
☑	66	443.4000	884.7854	885.5144	-0.7290	1	13	54	1	GARGSLGLR
☑	351	848.0700	1694.1254	1693.6503	0.4751	1	13	42	1	CKNGGNCVMDMYMR + Carbamidomethyl (C); Oxidation (M)
☑	297	752.4000	2254.1782	2253.0192	1.1589	1	13	42	1	RSASEQSPNVPHSSHMTETR + Oxidation (M)
☑	338	817.4800	1632.9454	1632.6847	0.2607	1	13	43	1	TVKGVPCCWHCER + 2 Carboxymethyl (C)
☑	67	443.5000	1327.4782	1328.5602	-1.0820	1	13	57	1	CQECNKSFTR + 2 Carbamidomethyl (C)
☑	323	794.5800	1587.1454	1586.7246	0.4208	0	13	48	1	MTYAEESATSATGLR
☑	10	338.2500	1011.7282	1010.3975	1.3307	0	13	58	1	CASSENTDK + Carbamidomethyl (C)
☑	407	946.8400	2837.4982	2836.3814	1.1168	0	13	39	1	EVVLLMNLPADSPAGPAEEGHHLPDGR + Oxidation (M)
☑	336	814.3600	1626.7054	1625.8494	0.8561	1	13	43	1	MAAAQPRLPAGAAMVR + Oxidation (M)
☑	446	1014.2700	3039.7882	3039.4985	0.2897	1	13	44	1	KNAVIVDTTICDPHYNLAHSSRPFER + Carbamidomethyl (C)
☑	158	579.3600	1156.7054	1157.5499	-0.8444	1	13	52	1	TSKGAAMSFSR + Oxidation (M)
☑	221	657.3100	1968.9082	1970.0255	-1.1173	1	13	46	1	SGKGAAAMIPGPQTVATEIR + Oxidation (M)
☑	93	482.6300	963.2454	963.4549	-0.2095	0	13	54	1	GFTDPSVEI
☑	488	1127.7100	3380.1082	3381.4379	-1.3297	1	13	40	1	NMCLNGMCINEDGSFKCICKPGFQLASDGR + Carbamidomethyl (C); Carboxymethyl (
☑	165	585.9700	1754.8882	1755.8574	-0.9692	1	13	51	1	GPGPKGPSGTMSEAQLAR + Oxidation (M)
☑	465	1062.2100	3183.6082	3182.6299	0.9783	1	13	41	1	QLICPICLEMFSKPVVILPCQHNLCRK + Carboxymethyl (C)
☑	250	695.8100	1389.6054	1390.6989	-1.0934	0	13	52	1	AICIEEIGVWMK
☑	391	919.3300	2754.9682	2755.4102	-0.4420	0	13	44	1	EPSPLHVLEGLEGSSAAEVPCIPSLPK
☑	22	377.4900	1129.4482	1128.5047	0.9435	0	13	54	1	ENEATTAEHK
☑	417	967.5700	2899.6882	2899.3732	0.3150	1	13	42	1	DEFIPTIVASSNLRVYTSVTHCDMK + Carboxymethyl (C); Oxidation (M)
☑	259	708.9100	1415.8054	1415.6391	0.1663	0	13	51	1	GIFGFTDSDCIGK + Carbamidomethyl (C)
☑	442	1005.9600	3014.8582	3016.4207	-1.5625	0	13	44	1	NCFEEELICPICLHVFVEPVQLPCK + Carbamidomethyl (C); Carboxymethyl (C)
☑	312	772.5800	2314.7182	2313.7995	0.9187	1	13	53	1	TSCCSCCPVGCAKCSQGCICK + Carbamidomethyl (C); 3 Carboxymethyl (C)
☑	330	805.0600	2412.1582	2411.1652	0.9929	1	13	47	1	DPGGQSGPGDPLTFGLCPQSRIR + Carbamidomethyl (C)
☑	370	879.8200	2636.4382	2637.1067	-0.6685	1	13	45	1	ETMCSSTNPIMAQCFDKSELSDK + Carbamidomethyl (C); Oxidation (M)
☑	98	491.6300	1471.8682	1472.6322	-0.7641	1	13	52	1	CACHICGGREAPEK
☑	380	896.5800	1791.1454	1790.8720	0.2734	0	13	48	1	GTANTCIPSISSIGTPSK + Carboxymethyl (C)
☑	231	670.0800	2007.2182	2006.1128	1.1053	0	13	56	1	ALLSYPLPFFAAVEVLEK
☑	441	1005.7000	2009.3854	2009.7991	-0.4137	1	13	47	1	LFEVDMDSCERWMSCK + 2 Oxidation (M)
☑	94	483.8000	1448.3782	1447.8147	0.5634	0	13	55	1	DGVPVPLIIAAGGGGR
☑	175	596.3300	1785.9682	1785.8250	0.1431	1	13	54	1	KGGCCSGTHPVAISQQR + Carboxymethyl (C)
☑	436	999.8900	1997.7654	1996.8441	0.9213	0	13	43	1	CELCDVSCTGADAYAAHIR
☑	92	480.5100	1438.5082	1439.7593	-1.2511	1	13	67	1	AAALAAGATGNGPSRR
☑	154	576.3300	1150.6454	1149.4543	1.1912	0	13	54	1	EEQATCPNCR
☑	288	741.2800	2220.8182	2220.1038	0.7144	1	13	48	1	FPHYFGKEVIGGMLDLEPR + Oxidation (M)
☑	189	620.3800	1858.1182	1856.8575	1.2607	0	13	52	1	SDMYAGTQEGVTAGSVLR + Oxidation (M)
☑	263	713.2800	2136.8182	2135.8573	0.9609	1	13	51	1	MWNDMACGTAYNWVCKK + 2 Carboxymethyl (C)
☑	378	894.1500	2679.4282	2678.1862	1.2420	0	13	51	1	NHCCPVCNMTFSSPVVAQSHYLGK + Carbamidomethyl (C)
☑	496	1199.8600	3596.5582	3595.5694	0.9887	0	13	43	1	CWSSDTYNPVPGVMDGVPSSNNYQGGFGTTLMAK + Oxidation (M)
☑	237	676.3200	2025.9382	2025.0465	0.8916	1	13	52	1	RPDLYALAMGYSGQLKSR
☑	220	657.1500	1312.2854	1310.7281	1.5573	0	13	60	1	WLCLGTLPRPR
☑	96	488.9900	975.9654	975.4661	0.4993	0	13	73	1	EEEPGLFR
☑	168	590.3400	1178.6654	1177.5979	1.0676	0	13	56	1	AADLLAADFSGK
☑	63	439.0800	1314.2182	1315.6554	-1.4372	1	13	70	1	MLPDLKADNQR + Oxidation (M)
☑	103	496.5100	991.0054	990.5320	0.4734	1	13	70	1	KAFMEPLR

☑	140	551.7600	1652.2582	1653.7127	-1.4545	0	13	65	1	VCSNPSGCSDIAYPK + 2 Carbamidomethyl (C)
☑	307	766.3400	1530.6654	1531.9081	-1.2427	1	13	51	1	LTIKLICMEIVLK + Oxidation (M)
☑	238	676.7700	1351.5254	1352.6473	-1.1218	1	13	55	1	HASSANQYKYGK
☑	48	423.5400	845.0654	844.4304	0.6351	1	12	72	1	AAADRWR
☑	358	857.4100	2569.2082	2570.2111	-1.0029	1	12	49	1	GEEECVLHYVENGYSLFLERK + Carbamidomethyl (C)
☑	354	850.6800	2549.0182	2549.5244	-0.5062	1	12	56	1	LLVIIVSLGYGIVKPRLGTVMHR + Oxidation (M)
☑	85	466.5800	1396.7182	1397.8031	-1.0849	1	12	65	1	WKVGLTVITPER
☑	215	650.8300	1949.4682	1948.8917	0.5764	1	12	61	1	LSCCLQEKPTCGSRGPR + Carbamidomethyl (C); Carboxymethyl (C)
☑	270	717.3700	1432.7254	1432.7092	0.0162	1	12	54	1	MDSGSLAAERPKR + Oxidation (M)
☑	471	1085.8300	2169.6454	2171.0173	-1.3719	1	12	49	1	VNRFGCIECLVTMAAASSNK + Carboxymethyl (C)
☑	295	747.6100	1493.2054	1493.6966	-0.4912	0	12	63	1	QVCEQLISGQMSR + Oxidation (M)
☑	348	839.2500	1676.4854	1677.8946	-1.4091	1	12	61	1	LYCQIAKTCPIQIK + Carbamidomethyl (C)
☑	398	931.3900	2791.1482	2792.2923	-1.1441	1	12	49	1	EVIMRADDLLPLGEDQTQDGDFGSR + Oxidation (M)
☑	276	726.2000	1450.3854	1451.5996	-1.2141	0	12	63	1	CLDHAPHCPLCK + 2 Carboxymethyl (C)
☑	112	508.9300	1523.7682	1522.7007	1.0675	1	12	69	1	ELKEQMECQLEK + Oxidation (M)
☑	123	528.4300	1582.2682	1581.6924	0.5757	1	12	70	1	CLWCKAMVHTSCK + Carbamidomethyl (C); Oxidation (M)
☑	292	746.8800	2237.6182	2237.8704	-0.2522	0	12	61	1	LYFYSYGSCNSSCMGPTER + Carboxymethyl (C); Oxidation (M)
☑	487	1124.0800	3369.2182	3369.4233	-0.2051	1	12	47	1	LSELHTYNSNFTCCSDGTMVSGKVWSCCPK + Carbamidomethyl (C); Oxidation (M)
☑	108	503.5900	1507.7482	1508.7358	-0.9877	1	12	67	1	KDEISVDSLDFNK
☑	464	1061.7000	3182.0782	3181.6225	0.4556	1	12	53	1	MAEGELRVDSFITCLLECLPIGVLAIFR + Carboxymethyl (C); Oxidation (M)
☑	227	667.7600	2000.2582	2001.0201	-0.7619	1	12	62	1	VKLDSSAGVSLSSGHVKPMS + Oxidation (M)
☑	279	732.5600	2194.6582	2193.8356	0.8225	0	12	66	1	VEAIQCQTCLSSCCSCSQK + 3 Carboxymethyl (C)
☑	87	467.3300	1398.9682	1397.6106	1.3576	1	12	71	1	CDPQGNHEKSAR + Carbamidomethyl (C)
☑	483	1116.0800	2230.1454	2230.1151	0.0304	0	12	49	1	AEEGIAAGGVMDVNTALQEVLK + Oxidation (M)
☑	406	943.9300	2828.7682	2828.2716	0.4966	1	12	54	1	VKAGMCTPETEPLPSQMMLFCWEK + Carbamidomethyl (C); Oxidation (M)
☑	321	790.7600	2369.2582	2369.2090	0.0492	1	12	56	1	FWFLEASTEAKVTSTMPLVGR
☑	2	307.8700	613.7254	612.2980	1.4275	0	12	66	1	QHGGSK
☑	229	668.8900	2003.6482	2002.9704	0.6778	1	12	63	1	SYVTMVTTALIDRMGDAK + 2 Oxidation (M)
☑	122	527.6600	1053.3054	1052.4862	0.8193	1	12	63	1	RCPPGYFR + Carboxymethyl (C)
☑	290	744.2100	1486.4054	1486.7239	-0.3184	1	12	67	1	TNPGKTCPVNGWTI
☑	472	1085.8500	2169.6854	2168.9329	0.7525	0	12	50	1	WGLICGDDWGTLEAMVACR + Carboxymethyl (C); Oxidation (M)
☑	235	674.9400	2021.7982	2020.8030	0.9951	0	12	62	1	DPDMFCDPFTSSSTTANK + Carboxymethyl (C)
☑	293	747.4400	1492.8654	1491.6987	1.1667	1	12	58	1	MDSQRELAEELR + Oxidation (M)
☑	422	977.3900	2929.1482	2928.4660	0.6822	1	12	52	1	RLVSMNMPLNSDGTVMFNATLFALVR + 2 Oxidation (M)
☑	394	926.6100	1851.2054	1850.7878	0.4176	1	12	62	1	GVQAREESSDSAEESDR
☑	448	1019.8600	3056.5582	3057.5811	-1.0229	1	12	52	1	WQLLSELEAAPYLPQEEKSPLFSVQR
☑	29	393.1500	1176.4282	1175.5493	0.8789	0	12	71	1	TTDHTMVDLK + Oxidation (M)
☑	353	849.5500	1697.0854	1697.8171	-0.7316	1	12	63	1	CGVPFFCWVRTLR + Carbamidomethyl (C); Carboxymethyl (C)
☑	194	623.3900	1867.1482	1865.9809	1.1673	1	12	69	1	KDTIVCLDGLFSVLGTK + Carboxymethyl (C)
☑	275	724.2000	2169.5782	2168.8885	0.6897	1	12	72	1	GRGNSSMDSTAGCSGTPPICR + 2 Carboxymethyl (C)
☑	381	899.4000	2695.1782	2694.1111	1.0671	1	12	58	1	RSMGMQRPLGMAGSGMGQSMEMER + 5 Oxidation (M)
☑	278	731.0800	2190.2182	2189.0941	1.1241	0	12	72	1	CFEAMPPFVTLLPNFVFLK + Carboxymethyl (C); Oxidation (M)
☑	36	406.8500	811.6854	812.4868	-0.8014	0	12	69	1	NQLAVLR
☑	199	629.2900	1884.8482	1885.9866	-1.1384	1	12	67	1	EVLSALSQLVPCVGCRR + Carbamidomethyl (C)
☑	379	896.3600	2686.0582	2685.3731	0.6851	1	12	58	1	CIKPNTQKMPDQFDQAVVLNQLR
☑	71	445.2700	888.5254	889.4869	-0.9614	1	12	80	1	ETGAAKVSK
☑	105	499.1100	1494.3082	1492.7816	1.5266	0	12	73	1	LLAAGVVSAMTCMVK
☑	191	621.4000	1861.1782	1859.9233	1.2548	1	12	68	1	MSSSNDPIVIPMIERR + Oxidation (M)
☑	310	770.8300	2309.4682	2308.1964	1.2717	0	12	67	1	SISFHPSGDFILVGTQHPTLR
☑	352	849.0300	2544.0682	2543.0257	1.0424	0	12	62	1	SGCEPCSSGSSFFTEFLDPFQR + 2 Carboxymethyl (C)
☑	368	875.8500	2624.5282	2623.1651	1.3630	1	12	63	1	NIVAMGSGFCDSFCCGNSTKAAVIR + Carbamidomethyl (C); Oxidation (M)
☑	271	717.6400	2149.8982	2150.1055	-0.2073	1	12	73	1	HTQATTLVCQIFGGYLRSR
☑	223	664.3000	1326.5854	1326.6390	-0.0536	1	12	69	1	FEIQKCQFER
☑	269	716.8000	1431.5854	1431.6711	-0.0856	1	12	69	1	AVMAQQPNMDRR + Oxidation (M)
☑	473	1087.6400	3259.8982	3259.5893	0.3088	0	12	57	1	ELVHQSNTSNIFFSPMSITTAFAMLSVGSK + Oxidation (M)
☑	183	614.1400	1226.2654	1226.7751	-0.5096	1	11	81	1	LRVPLFEILK
☑	474	1088.7300	3263.1682	3263.6278	-0.4596	1	11	64	1	ASALEQFVNSVRQLSAQGQMTQLCELINK + Carboxymethyl (C)
☑	272	717.8000	1433.5854	1434.8095	-1.2241	1	11	70	1	RIYIPLPEAHR
☑	145	556.4300	1666.2682	1666.7919	-0.5238	0	11	83	1	LAHCDLGTDHGLLMR + Oxidation (M)
☑	118	519.3700	1555.0882	1556.6455	-1.5574	0	11	78	1	LMMLQSCSGPTCR + Carbamidomethyl (C); Carboxymethyl (C); Oxidation (M)
☑	287	739.3900	2215.1482	2214.0939	1.0543	1	11	70	1	SICSLRYQLHPAFLCGHGR + Carbamidomethyl (C)
☑	454	1037.8200	3110.4382	3111.2539	-0.8158	1	11	61	1	LNQCTDSCVRSDMPSVSSCDGHSWGPR + 2 Carbamidomethyl (C); Carboxymethyl (C
☑	440	1005.4400	3013.2982	3014.4518	-1.1536	1	11	58	1	IPAHRLVLSSVSDYFAAMFTSDVCEAK + Carboxymethyl (C)
☑	228	668.4100	1334.8054	1334.7306	0.0748	0	11	74	1	APPAAATAASLPAAR
☑	364	863.0300	1724.0454	1722.7553	1.2902	0	11	65	1	QEQGSLANTDCQLMK + Carboxymethyl (C)
☑	405	935.8900	2804.6482	2805.1723	-0.5241	1	11	64	1	EQGMIGGCVCVCVLSEKQMWQECR + Carboxymethyl (C); 2 Oxidation (M)
☑	49	423.5400	845.0654	844.4766	0.5888	1	11	95	1	LQKATER
☑	184	614.2000	1839.5782	1838.7200	0.8582	0	11	74	1	CDDSWDINDANVVCR + Carbamidomethyl (C); Carboxymethyl (C)
☑	248	693.2300	2076.6682	2075.7554	0.9128	1	11	75	1	RCECSSAGDSACATFCHR + Carbamidomethyl (C); 2 Carboxymethyl (C)
☑	214	648.7900	1295.5654	1296.5326	-0.9672	0	11	77	1	DCSSIPGATGTCK + Carboxymethyl (C)
☑	356	853.7400	2558.1982	2559.3553	-1.1571	1	11	71	1	MSPEIALNRISPMLSPFISSVVR + Oxidation (M)
☑	162	583.3700	1747.0882	1747.9329	-0.8447	1	11	79	1	REGSGNPTPLINPLAGR
☑	202	633.1900	1896.5482	1898.0070	-1.4589	0	11	78	1	AASPLETCLLELEVSPVK
☑	481	1109.4600	2216.9054	2218.1494	-1.2440	0	11	57	1	GAGAPGAAVHSPLSLGSPRPYEK
☑	499	1254.8500	3761.5282	3762.9113	-1.3831	1	11	59	1	SGENPYPPLNLLADFGGGGLMCTLGILLALFERTR + Carboxymethyl (C)
☑	27	388.2700	1161.7882	1160.5682	1.2200	1	11	93	1	MMSLADKTHK
☑	26	387.6300	1159.8682	1158.6873	1.1808	1	11	96	1	VPVKHLVPDR
☑	361	859.6600	2575.9582	2576.0400	-0.0818	1	11	79	1	QHMDSDSPSKSSPTYCNVMMSGR + 2 Oxidation (M)
☑	280	733.0100	2196.0082	2195.0728	0.9353	1	11	75	1	GQCRVQHLHFPSVVDMLR + Carboxymethyl (C); Oxidation (M)
☑	284	737.4000	2209.1782	2210.0426	-0.8644	1	11	74	1	YAKGPNYVCAQEVNLQNDK + Carbamidomethyl (C)
☑	341	827.6300	2479.8682	2481.1734	-1.3052	0	11	79	1	LSLGTGSSYGLQVPWMDSTPEEK
☑	469	1077.0200	3228.0382	3229.2814	-1.2432	0	11	65	1	LSLCLCLCVCVCVCVCVCVCVCPCTR + Carbamidomethyl (C); 4 Carboxymethyl (
☑	24	381.3900	1141.1482	1139.5683	1.5799	1	11	1.1e+02	1	QHTKEINDR
☑	428	992.8100	2975.4082	2975.4851	-0.0769	1	11	67	1	TAWELPKTYLAPAHVGSGAYGAVCSAIDK
☑	82	460.6600	1378.9582	1379.6173	-0.6592	1	11	90	1	SNPSDIDVCRMK + Oxidation (M)
☑	365	864.1800	2589.5182	2589.2421	0.2761	0	11	83	1	ESFVGAGFASFDLVAQMTAEDLLR + Oxidation (M)
☑	445	1011.8500	3032.5282	3031.4590	1.0692	1	11	68	1	DVMEGSAMLIQEAKQALIAPGDTESQQR + Oxidation (M)
☑	4	316.5200	631.0254	631.3177	-0.2922	0	11	1.1e+02	1	AEVGEK
☑	360	857.7100	1713.4054	1713.8066	-0.4011	1	11	78	1	LCKEGPAATTSMAYAK + Carbamidomethyl (C); Oxidation (M)
☑	267	716.3900	1430.7654	1429.7487	1.0168	0	11	77	1	EVVPPSIIMSSQK + Oxidation (M)
☑	25	385.1100	768.2054	768.4494	-0.2439	0	11	81	1	ALTLHSK
☑	294	747.6100	1493.2054	1493.6609	-0.4555	0	11	87	1	QTQLWTHEYCK + Carboxymethyl (C)
☑	251	696.5100	2086.5082	2088.0521	-1.5439	1	11	91	1	TRELQTMADQETVSPAAIK
☑	207	642.2400	1923.6982	1922.9329	0.7652	0	11	79	1	EELGTVNGMTITMVGDLK + Oxidation (M)
☑	45	420.3300	1257.9682	1256.4849	1.4833	0	11	88	1	MHECHQCQK + 2 Carbamidomethyl (C)
☑	313	777.1200	1552.2254	1551.6203	0.6051	0	11	89	1	WNTCCTHCIMAR + 2 Carbamidomethyl (C)
☑	152	568.9100	1135.8054	1134.5591	1.2464	0	11	88	1	MTATAAVETPK + Oxidation (M)
☑	309	770.2800	2307.8182	2307.1611	0.6570	1	11	79	1	LKLFLTPHMCLMASLICSR + Carbamidomethyl (C); Carboxymethyl (C); Oxidati
☑	204	635.2400	1902.6982	1901.9088	0.7894	0	11	86	1	DIHNMNNGSTVVCTLIR + Oxidation (M)
☑	170	590.3500	1768.0282	1766.8145	1.2137	0	11	89	1	MPTDLPPASEDAGPDVR
☑	349	841.6400	1681.2654	1679.6854	1.5800	1	11	87	1	SHPEGKADCCFDCLR
☑	246	691.2800	1380.5454	1380.7837	-0.2383	1	11	82	1	SLLETRLHVTGR
☑	113	509.6400	1525.8982	1524.6847	1.2135	1	11	95	1	ACRSCSIPCGSAVSK + Carbamidomethyl (C)
☑	400	932.4900	2794.4482	2795.2968	-0.8487	1	11	73	1	YDKIEDMAMMTHLHEPGVLYNLK + 3 Oxidation (M)
☑	106	500.5100	1498.5082	1499.8136	-1.3054	1	10	1.1e+02	1	KNNFIIISNYFK
☑	435	998.7600	2993.2582	2994.3546	-1.0965	1	10	81	1	MERSSTSVSPNNSITINTMYDTTAQSK + 2 Oxidation (M)
☑	495	1198.7800	2395.5454	2394.2002	1.3453	1	10	71	1	ERNRPEVFEVIQEMFQISK + Oxidation (M)
☑	452	1024.0200	3069.0382	3068.3527	0.6855	0	10	75	1	TIDFDCEDDDMNLNCFILMFDLLLK + Carbamidomethyl (C); Oxidation (M)
☑	490	1141.0200	2280.0254	2279.0382	0.9872	0	10	71	1	GGESIFGQLYGDQASFFEAEK
☑	185	615.4100	1228.8054	1229.6299	-0.8244	1	10	93	1	CSAPARLTGAGAR
☑	173	593.3600	1777.0582	1777.8846	-0.8265	0	10	93	1	TSLPPVGPSGLYSSSSSR

☑	386	912.3900	2734.1482	2734.2148	-0.0666	1	10	80	1	TTAATGGHSCSQSNLRGAGDLQGTCQR + Carboxymethyl (C)
☑	86	466.7100	1397.1082	1396.5963	0.5119	0	10	1.1e+02	1	STVGMCSNDLGEK + Carbamidomethyl (C)
☑	387	913.9500	2738.8282	2737.3091	1.5190	1	10	83	1	NGELDFSTFLTIMHMQIKQEDPK + Oxidation (M)
☑	79	458.5500	1372.6282	1371.5482	1.0800	1	10	1.1e+02	1	NKHICGHDCCK + Carbamidomethyl (C); Carboxymethyl (C)
☑	316	785.2100	1568.4054	1566.8518	1.5536	0	10	93	1	AGPGGPPPPPEIAAALR
☑	494	1192.4100	3574.2082	3572.6118	1.5964	0	10	73	1	MVTPIPEGFLLISSPPCPLGNCAHECGVCGSQK + Carbamidomethyl (C); 2 Carboxymet
☑	242	686.1200	2055.3382	2055.9718	-0.6336	0	10	1e+02	1	GGVSTTPPEPSCCAQLGLPSR
☑	90	475.4700	1423.3882	1424.7342	-1.3460	1	10	1.2e+02	1	RKPILMCFLCK + Carboxymethyl (C); Oxidation (M)
☑	180	605.9500	1209.8854	1208.5343	1.3511	0	10	94	1	ICAASDDVGSSK + Carbamidomethyl (C)
☑	245	690.6500	2068.9282	2068.9598	-0.0316	0	10	99	1	YETNLTFVGCVGMLDPPR + Carboxymethyl (C)
☑	482	1111.7100	3332.1082	3333.6283	-1.5202	1	10	78	1	IMGNGFAGCVHFPHTAPCEVRVLMLLYSSK + Carbamidomethyl (C)
☑	438	1003.5800	3007.7182	3008.4120	-0.6938	0	10	79	1	LICEGHYGPGMAELEQQVAEHNILQR + Carboxymethyl (C); Oxidation (M)
☑	289	742.8900	2225.6482	2227.0255	-1.3774	0	10	98	1	LWNSLCNSEDPYNLLNFK + Carboxymethyl (C)
☑	19	363.6000	1087.7782	1086.5921	1.1861	0	10	99	1	GEEVTILAQK
☑	411	957.6400	2869.8982	2871.4001	-1.5019	1	10	90	1	DICPNDENIFPVTSYIGTATQKIFVS
☑	421	977.2300	2928.6682	2928.3352	0.3329	0	10	91	1	VCSYLMLGSYLMGFSDAMIHTGCILR + 3 Oxidation (M)
☑	59	433.4200	1297.2382	1295.6908	1.5474	0	10	1.2e+02	1	TVAPGVVPAPCASK
☑	126	532.4800	1062.9454	1062.5240	0.4214	1	10	1.2e+02	1	ATRIASEGCR
☑	135	543.3400	1626.9982	1627.8828	-0.8846	1	10	1.1e+02	1	MVQAQGILLRDNVR + Oxidation (M)
☑	355	851.6600	2551.9582	2552.1887	-0.2305	1	10	99	1	LQPGQVCSSKVQLCYVGAGDEEK + Carbamidomethyl (C); Carboxymethyl (C)
☑	167	588.5200	1762.5382	1763.8804	-1.3422	0	10	1.3e+02	1	LFDISMAISYLYNSK
☑	37	411.2100	820.4054	820.4000	0.0054	0	10	1.2e+02	1	CTDILEK
☑	423	980.7000	2939.0782	2937.8873	1.1908	1	10	93	1	GGCGSCGGCSSCGGCKGGCGSCGGCGSCGGCK + 5 Carbamidomethyl (C); Carboxymeth
☑	115	513.0800	1536.2182	1536.7714	-0.5532	1	10	1.2e+02	1	LMAKMESVGPMTVK + Oxidation (M)
☑	31	402.5300	1204.5682	1205.6808	-1.1127	0	10	1.3e+02	1	KPSLLSFWTK
☑	155	577.2300	1728.6682	1727.9029	0.7653	0	10	1.1e+02	1	TPGVMDSFLVLHQLR + Oxidation (M)
☑	315	784.0400	2349.0982	2350.0830	-0.9848	0	10	94	1	IDMHADGTQDLMMMIIGAPFK + Oxidation (M)
☑	340	824.6600	2470.9582	2471.0991	-0.1410	1	10	1.1e+02	1	CINLEGSYTCQCLTARDANPSR + Carbamidomethyl (C)
☑	186	616.2300	1845.6682	1846.0537	-0.3855	1	10	1e+02	1	ASGPGRLLAGGAGLAVPGGLR
☑	205	638.9400	1275.8654	1274.6540	1.2114	0	10	1e+02	1	STMPTSLPNLAK + Oxidation (M)
☑	476	1100.4200	3298.2382	3297.6989	0.5393	0	10	84	1	MPTTPLLLAALAALAALAVAAYSSCSPGPDPSGK + Carbamidomethyl (C); Oxidation (
☑	212	645.3700	1933.0882	1932.8280	0.2601	1	10	1e+02	1	CRCLVEYGGEFCQEAAR
☑	254	702.4500	2104.3282	2105.1456	-0.8174	1	10	1.1e+02	1	LRVWGECLEALPGLPPTVR
☑	461	1054.9800	2107.9454	2107.1174	0.8280	1	10	87	1	QIQEVFNQFRVVLSSSAR
☑	497	1214.1400	3639.3982	3640.4343	-1.0362	1	10	77	1	WQCIECKSCILCGTSENDDQLLFCDDCDR + 4 Carbamidomethyl (C); Carboxymethyl
☑	384	906.3500	2716.0282	2716.9937	-0.9655	1	10	92	1	EDAAGSDREMNEDDSTDCDDDIQR + Oxidation (M)
☑	224	665.4800	1328.9454	1328.6725	0.2730	1	10	1.2e+02	1	KLHTSDWLSDK
☑	388	915.0800	2742.2182	2742.1996	0.0186	1	10	94	1	GFRCPLHSCMSCHASNPSNPRPSK + 2 Carbamidomethyl (C); Oxidation (M)
☑	107	501.9000	1502.6782	1501.7736	0.9046	1	10	1.2e+02	1	EEEVSLQGINTRK
☑	332	810.8700	1619.7254	1619.8552	-0.1298	1	9	1.1e+02	1	NLNMSTKGALLSDLK + Oxidation (M)
☑	493	1179.1000	3534.2782	3535.6568	-1.3786	1	9	85	1	TVGSRMTTAPAPMEVTCSGSLSVAWGPTMGPVGAR + Carboxymethyl (C); Oxidation (M
☑	402	935.2700	2802.7882	2803.4006	-0.6124	0	9	1.1e+02	1	CVLLGPVVPATCAPCAPAACVPVPAASGR + 2 Carbamidomethyl (C)
☑	226	667.5100	1999.5082	2001.0419	-1.5337	1	9	1.3e+02	1	EILPEAVKDTGDVVFANGK
☑	367	871.8300	2612.4682	2610.9799	1.4883	0	9	98	1	DDFSCPYLHGDVCSGHGECEGGR + 2 Carbamidomethyl (C); Carboxymethyl (C)
☑	319	789.2000	2364.5782	2364.2116	0.3666	1	9	1.2e+02	1	TLLHCAAGVSRSAALCLAYLMK + Carbamidomethyl (C); Oxidation (M)
☑	377	892.2200	2673.6382	2674.4655	-0.8273	1	9	1.2e+02	1	LIVNLSQTYIAMYLTYSLSLPKK + Oxidation (M)
☑	283	735.8100	1469.6054	1470.6635	-1.0581	0	9	1.2e+02	1	AEWMIAAQTCFK + Carbamidomethyl (C); Oxidation (M)
☑	302	759.7600	2276.2582	2276.1017	0.1565	1	9	1.1e+02	1	MCGWSPRNPRPTGLFLPMK + Carbamidomethyl (C); 2 Oxidation (M)
☑	329	805.0200	2412.0382	2410.9659	1.0723	0	9	1.1e+02	1	ADCGHPCMAPCHPSLPCPVTACK + Carboxymethyl (C); Oxidation (M)
☑	260	710.0200	2127.0382	2126.8820	0.1562	0	9	1.2e+02	1	CQGFTSVPSHGGCTLGMASR + 2 Carboxymethyl (C); Oxidation (M)
☑	234	673.3000	1344.5854	1345.5941	-1.0087	0	9	1.2e+02	1	APCPVEVVCCR + 3 Carbamidomethyl (C)
☑	498	1245.1100	3732.3082	3731.6881	0.6201	0	9	93	1	ASLSFAVCWSTQAVCGWNSATIVPSWALCSCTTAP + 2 Carbamidomethyl (C)
☑	489	1135.9100	3404.7082	3405.5436	-0.8355	1	9	92	1	LGVLGYGDQIEMYHMNQNAGMREMVFMQK + Oxidation (M)
☑	447	1018.8100	3053.4082	3052.2606	1.1476	1	9	1.1e+02	1	CSPGYTGALCQDECPVGTYGVRCAETCR + 2 Carbamidomethyl (C)
☑	299	754.2400	2259.6982	2259.0147	0.6834	0	9	1.2e+02	1	EQGACGLGTAEVNVHLAEMEK + Carboxymethyl (C); Oxidation (M)
☑	8	325.4800	973.4182	972.4335	0.9847	0	9	1.8e+02	1	APTQGCNPK + Carboxymethyl (C)
☑	486	1121.0500	3360.1282	3360.6085	-0.4803	0	9	1.1e+02	1	VHPGESNSSWIMNGFLDFILSDSPDAQLLR + Oxidation (M)
☑	433	997.4700	1992.9254	1993.9900	-1.0645	1	9	1.1e+02	1	MVGVPAAFDMMLTGRNIR + Oxidation (M)
☑	382	902.2700	2703.7882	2704.0597	-0.2715	1	9	1.3e+02	1	DCEMKTCHPEHFQCTSGHCVPK + 2 Carbamidomethyl (C); Carboxymethyl (C); Ox
☑	444	1011.0200	3030.0382	3028.4768	1.5614	1	9	1.2e+02	1	WWQLPYYWVGIKMYDLVAGSHCLK + Carbamidomethyl (C); Oxidation (M)
☑	457	1044.7700	3131.2882	3130.4688	0.8194	1	9	1.2e+02	1	MDLGLFGFNRHLFCGGLNMFGQGSGNIR + Carbamidomethyl (C); Oxidation (M)
☑	430	994.3400	1986.6654	1988.0844	-1.4189	0	9	1.2e+02	1	HVPGGGSVHIVYKPVDLSK
☑	318	788.5400	2362.5982	2363.1647	-0.5665	1	8	1.4e+02	1	SQLELLISPTCSCKQADICVR + Carbamidomethyl (C)
☑	100	493.6300	1477.8682	1478.8205	-0.9523	0	8	1.5e+02	1	LPSVSQLINPQQR
☑	424	981.8300	2942.4682	2943.1555	-0.6873	1	8	1.2e+02	1	GSFQCVCTDPYMLGEDEKTCIDSGR + 3 Carboxymethyl (C); Oxidation (M)
☑	485	1119.1500	3354.4282	3354.5988	-0.1707	1	8	1.1e+02	1	RGVTFTEHFPNPLLWFMLCMLGPGSGTTR + Carboxymethyl (C); 2 Oxidation (M)
☑	470	1081.2800	3240.8182	3240.5222	0.2960	1	8	1.3e+02	1	QRQPMPASMPGTLPNPTMPGSSAVLMPMER + 2 Oxidation (M)
☑	484	1118.9300	2235.8454	2235.0091	0.8364	1	8	1.1e+02	1	CCLLHQKLQMLNCCIER + 2 Carbamidomethyl (C); Carboxymethyl (C); Oxidati
☑	1	301.7500	902.2282	901.4730	0.7552	1	8	2.1e+02	1	RGAGSAEVR
☑	325	797.4300	2389.2682	2389.9173	-0.6491	1	8	1.4e+02	1	MRMMAMQMMTNSITDAMER + 7 Oxidation (M)
☑	350	842.7900	1683.5654	1683.8289	-0.2634	1	8	1.3e+02	1	TERLVHSNDGTQTAR
☑	255	703.6400	2107.8982	2106.9562	0.9420	1	8	1.6e+02	1	SQTLNPTQDPSECVKEMK + Carbamidomethyl (C); Oxidation (M)
☑	347	837.6200	1673.2254	1671.8540	1.3714	1	8	1.5e+02	1	KNSSRPAVTQGTAPSTA
☑	344	832.3700	1662.7254	1662.8941	-0.1686	1	8	1.3e+02	1	NAAQFLLSTNDKTIK
☑	450	1021.5500	2041.0854	2042.0531	-0.9677	1	8	1.3e+02	1	GKASIEIANESGETPLDIAK
☑	174	595.2100	1782.6082	1782.9588	-0.3506	1	8	1.6e+02	1	NLDRQAGLAVVLETER
☑	306	766.0400	2295.0982	2295.0706	0.0275	1	8	1.5e+02	1	VFSKLMSVCVMFTNCMQVR + Carbamidomethyl (C); Oxidation (M)
☑	477	1102.3300	3303.9682	3302.5093	1.4588	1	8	1.3e+02	1	CVIINHLNSGWGWNDIPCSGKQQSVCQMK + Carboxymethyl (C)
☑	389	916.8300	2747.4682	2746.1294	1.3388	1	8	1.3e+02	1	VAPSGAMSDSESSSSDSSDAEELARCR + Oxidation (M)
☑	264	714.4500	2140.3282	2140.9704	-0.6422	1	8	1.6e+02	1	MVASHNEDTVSFTLCRMK + Carbamidomethyl (C); Oxidation (M)
☑	150	565.6100	1693.8082	1692.8011	1.0071	0	8	1.9e+02	1	NVGLMICGHVHMGPR + Carbamidomethyl (C); Oxidation (M)
☑	456	1041.1800	3120.5182	3121.6534	-1.1352	1	7	1.4e+02	1	TVSHRDGAHLLPGPESFPGLQRPTQVLGR
☑	392	921.9000	2762.6782	2761.1876	1.4906	1	6	1.9e+02	1	MHCDKQFTSFPFQHFYCDQPAK + Carbamidomethyl (C)

Search Parameters

```
Type of search         : MS/MS Ion Search
Enzyme                 : Trypsin
Variable modifications : Carbamidomethyl (C),Carboxymethyl (C),Oxidation (M)
Mass values            : Monoisotopic
Protein Mass           : Unrestricted
Peptide Mass Tolerance : ± 1.6 Da
Fragment Mass Tolerance: ± 0.8 Da
Max Missed Cleavages   : 1
Instrument type        : ESI-TRAP
Number of queries      : 500
```

Mascot: http://www.matrixscience.com/

MASCOT Search Results

**User**	: pbm
**E-mail**	:
**Search title**	: Submitted from MRosenberg Data2009 by Mascot Daemon on SCT-14-65A4
**MS data file**	: G:\PRESTATIONS\Labos académiques\Internationaux\Etats Unis\Martina Rosenberg\Data pour publi M Rosenberg\Identifications Mascot\MR__2116.mgf
**Database**	: NCBIprot 20171205 (139,213,787 sequences; 51,013,024,959 residues)
**Taxonomy**	: Rattus (77,467 sequences)
**Timestamp**	: 25 Jan 2019 at 10:10:45 GMT

Not what you expected? Try **the peptide summary**.

Search parameters

Score distribution

Modification statistics

Legend

Protein Family Summary

Significance threshold p<	0.05	Max. number of families	20
Display non-sig. matches	☐	Dendrograms cut at	0
Preferred taxonomy	All entries		

Sensitivity

Protein families 1–8 (out of 8)

10 per page 1

**1**	**NP_036767.1**	111	anionic trypsin-1 precursor [Rattus norvegicus]
**2**	**NP_001009542.1**	103	programmed cell death protein 10 [Rattus norvegicus]

		Score	Mass	Matches	Sequences	emPAI
**2.1**	**NP_001009542.1** programmed cell death protein 10 [Rattus norvegicus]	103	24340	4 (4)	4 (4)	1.04

4 peptide matches (4 non-duplicate, 0 duplicate)

Query	Dupes	Observed	Mr(expt)	Mr(calc)	Delta	M	Score	Expect	Rank	U	Peptide
190		536.8200	1071.6254	1071.6036	0.0218	0	64	5.3e-05	1	U	R.VNLSAAQTLR.A
197		552.8300	1103.6454	1103.6087	0.0367	0	45	0.0043	1	U	K.AINVFISANR.L
327		784.7100	1567.4054	1567.9086	-0.5032	0	41	0.0022	1	U	R.LIHQTNLILQTFK.T
426		977.2700	2928.7882	2929.2633	-0.4751	0	50	0.0017	1	U	R.MAADDVEEYMIERPEPEFQDLNEK.A + 2 Oxidation (M)

**3**	**XP_008774848.1**	101	PREDICTED: LOW QUALITY PROTEIN: keratin, type II cytoskeletal 6A-like [Rattus norvegicus]
**4**	**NP_001008751.1**	59	keratin, type I cytoskeletal 14 [Rattus norvegicus]
**5**	**NP_001008802.2**	52	keratin, type II cytoskeletal 1 [Rattus norvegicus]
**6**	**XP_006247453.1**	50	PREDICTED: keratin, type I cytoskeletal 10 isoform X2 [Rattus norvegicus]
**7**	**XP_008770740.1**	40	PREDICTED: nuclear factor of activated T-cells, cytoplasmic 3 isoform X1 [Rattus norvegicus]
**8**	**EDM02456.1**	34	pam, highwire, rpm 1 (predicted) [Rattus norvegicus]

10 per page 1

Not what you expected? Try **the peptide summary**.

Mascot: http://www.matrixscience.com/

MATRIX SCIENCE MASCOT Search Results

Protein View: NP_001009542.1

programmed cell death protein 10 [Rattus norvegicus]

**Database:**	NCBIprot
**Score:**	103
**Monoisotopic mass (M_r):**	24340
**Calculated pI:**	6.78
**Taxonomy:**	Rattus norvegicus

This protein sequence matches the following other entries:

- XP_006232563.1 from Rattus norvegicus
- Q6NX65.1 from Rattus norvegicus
- AAH67245.1 from Rattus norvegicus
- EDM00884.1 from Rattus norvegicus

Sequence similarity is available as an NCBI BLAST search of NP_001009542.1 against nr.

Search parameters

**MS data file:**	G:\PRESTATIONS\Labos académiques\Internationaux\Etats Unis\Martina Rosenberg\Data pour publi M Rosenberg\Identifications Mascot\MR__2116.mgf
**Enzyme:**	Trypsin: cuts C-term side of KR unless next residue is P.
**Variable modifications:**	Carbamidomethyl (C), Oxidation (M)

Protein sequence coverage: 27%

Matched peptides shown in ***bold red***.

1 MTMEEMKTEA EAASMVSMPL YAVMYPVFNE LER**VNLSAAQ TLR**AAFIKAE
51 KENPGLTQDI IMKILEKKSV EVNFTESLLR **MAADDVEEYM IERPEPEFQD**
101 **LNEK**ARALKQ ILSKIPDEIN DRVRFLQTIK DIASAIKELL DTVNNVFKKY
151 QYQNRRALEH QKKEFVKYSK SFSDTLKTYF KDGK**AINVFI SANRLIHQTN**
201 **LILQTFK**TVA

Unformatted sequence string: 210 residues (for pasting into other applications).

Sort by ◉ residue number ○ increasing mass ○ decreasing mass
Show ◉ matched peptides only ○ predicted peptides also

Query	Start – End	Observed	Mr(expt)	Mr(calc)	Delta	M	Score	Expect	Rank	U	Peptide
190	34 – 43	536.8200	1071.6254	1071.6036	0.0218	0	64	5.3e-05	1	U	R.VNLSAAQTLR.A
426	81 – 104	977.2700	2928.7882	2929.2633	-0.4751	0	50	0.0017	1	U	R.MAADDVEEYMIERPEPEFQDLNEK.A + 2 Oxidation (M)
197	185 – 194	552.8300	1103.6454	1103.6087	0.0367	0	45	0.0043	1	U	K.AINVFISANR.L
327	195 – 207	784.7100	1567.4054	1567.9086	-0.5032	0	41	0.0022	1	U	R.LIHQTNLILQTFK.T

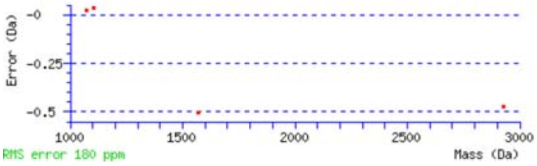

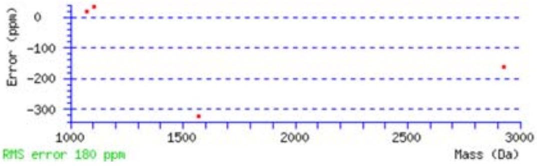


```
LOCUS       NP_001009542             210 aa            linear   ROD 19-JAN-2019
DEFINITION  programmed cell death protein 10 [Rattus norvegicus].
ACCESSION   NP_001009542
VERSION     NP_001009542.1
DBSOURCE    REFSEQ: accession NM_001009542.2
KEYWORDS    RefSeq.
SOURCE      Rattus norvegicus (Norway rat)
  ORGANISM  Rattus norvegicus
            Eukaryota; Metazoa; Chordata; Craniata; Vertebrata; Euteleostomi;
            Mammalia; Eutheria; Euarchontoglires; Glires; Rodentia; Myomorpha;
            Muroidea; Muridae; Murinae; Rattus.
REFERENCE   1  (residues 1 to 210)
  AUTHORS   Mardakheh FK, Self A and Marshall CJ.
  TITLE     RHO binding to FAM65A regulates Golgi reorientation during cell
            migration
  JOURNAL   J. Cell. Sci. 129 (24), 4466-4479 (2016)
   PUBMED   27807006
REFERENCE   2  (residues 1 to 210)
  AUTHORS   Zhang M, Dong L, Shi Z, Jiao S, Zhang Z, Zhang W, Liu G, Chen C,
            Feng M, Hao Q, Wang W, Yin M, Zhao Y, Zhang L and Zhou Z.
  TITLE     Structural mechanism of CCM3 heterodimerization with GCKIII kinases
  JOURNAL   Structure 21 (4), 680-688 (2013)
   PUBMED   23541896
REFERENCE   3  (residues 1 to 210)
  AUTHORS   You C, Sandalcioglu IE, Dammann P, Felbor U, Sure U and Zhu Y.
  TITLE     Loss of CCM3 impairs DLL4-Notch signalling: implication in
            endothelial angiogenesis and in inherited cerebral cavernous
            malformations
  JOURNAL   J. Cell. Mol. Med. 17 (3), 407-418 (2013)
   PUBMED   23388056
REFERENCE   4  (residues 1 to 210)
  AUTHORS   Kim HW, Mallick F, Durrani S, Ashraf M, Jiang S and Haider KH.
  TITLE     Concomitant activation of miR-107/PDCD10 and hypoxamir-210/Casp8ap2
            and their role in cytoprotection during ischemic preconditioning of
            stem cells
  JOURNAL   Antioxid. Redox Signal. 17 (8), 1053-1065 (2012)
   PUBMED   22482882
  REMARK    GeneRIF: The ischemic preconditioning (IPC) enhances stem cell
            survival via the combined participation of hypoxia responsive miRs
            miR-107 and miR-210 via their respective putative target genes
            Pdcd10 and Casp8ap2.
```

```
REFERENCE   5  (residues 1 to 210)
  AUTHORS   Zhang H, Ma X, Deng X, Chen Y, Mo X, Zhang Y, Zhao H and Ma D.
  TITLE     PDCD10 interacts with STK25 to accelerate cell apoptosis under
            oxidative stress
  JOURNAL   Front Biosci (Landmark Ed) 17, 2295-2305 (2012)
   PUBMED   22652780
  REMARK    Publication Status: Online-Only
REFERENCE   6  (residues 1 to 210)
  AUTHORS   Buschow SI, van Balkom BW, Aalberts M, Heck AJ, Wauben M and
            Stoorvogel W.
  TITLE     MHC class II-associated proteins in B-cell exosomes and potential
            functional implications for exosome biogenesis
  JOURNAL   Immunol. Cell Biol. 88 (8), 851-856 (2010)
   PUBMED   20458337
REFERENCE   7  (residues 1 to 210)
  AUTHORS   Tarantino C, Paolella G, Cozzuto L, Minopoli G, Pastore L, Parisi S
            and Russo T.
  TITLE     miRNA 34a, 100, and 137 modulate differentiation of mouse embryonic
            stem cells
  JOURNAL   FASEB J. 24 (9), 3255-3263 (2010)
   PUBMED   20439489
REFERENCE   8  (residues 1 to 210)
  AUTHORS   Fidalgo M, Fraile M, Pires A, Force T, Pombo C and Zalvide J.
  TITLE     CCM3/PDCD10 stabilizes GCKIII proteins to promote Golgi assembly
            and cell orientation
  JOURNAL   J. Cell. Sci. 123 (Pt 8), 1274-1284 (2010)
   PUBMED   20332113
REFERENCE   9  (residues 1 to 210)
  AUTHORS   Gonzales PA, Pisitkun T, Hoffert JD, Tchapyjnikov D, Star RA, Kleta
            R, Wang NS and Knepper MA.
  TITLE     Large-scale proteomics and phosphoproteomics of urinary exosomes
  JOURNAL   J. Am. Soc. Nephrol. 20 (2), 363-379 (2009)
   PUBMED   19056867
REFERENCE   10 (residues 1 to 210)
  AUTHORS   Ma X, Zhao H, Shan J, Long F, Chen Y, Chen Y, Zhang Y, Han X and Ma
            D.
  TITLE     PDCD10 interacts with Ste20-related kinase MST4 to promote cell
            growth and transformation via modulation of the ERK pathway
  JOURNAL   Mol. Biol. Cell 18 (6), 1965-1978 (2007)
   PUBMED   17360971
COMMENT     PROVISIONAL REFSEQ: This record has not yet been subject to final
            NCBI review. The reference sequence was derived from BC067245.1.

            Publication Note:  This RefSeq record includes a subset of the
            publications that are available for this gene. Please see the Gene
            record to access additional publications.

            ##Evidence-Data-START##
            Transcript exon combination :: BC067245.1, FQ213019.1 [ECO:0000332]
            RNAseq introns              :: single sample supports all introns
                                           SAMN01906347, SAMN01906349
                                           [ECO:0000348]
            ##Evidence-Data-END##
FEATURES             Location/Qualifiers
     source          1..210
                     /organism="Rattus norvegicus"
                     /db_xref="taxon:10116"
                     /chromosome="2"
                     /map="2q32"
     Protein         1..210
                     /product="programmed cell death protein 10"
                     /calculated_mol_wt=24224
     Region          12..159
                     /region_name="DUF1241"
                     /note="Protein of unknown function (DUF1241); pfam06840"
                     /db_xref="CDD:284303"
     Site            177
                     /site_type="other"
                     /experiment="experimental evidence, no additional details
                     recorded"
                     /note="N6-acetyllysine. {ECO:0000250|UniProtKB:Q9BUL8};
                     propagated from UniProtKB/Swiss-Prot (Q6NX65.1)"
     CDS             1..210
                     /gene="Pdcd10"
                     /coded_by="NM_001009542.2:208..840"
                     /db_xref="GeneID:494345"
                     /db_xref="RGD:1359329"
```

Mascot: http://www.matrixscience.com/

MATRIX SCIENCE MASCOT Search Results

User : pbm
E-mail :
Search title : Submitted from MRosenberg Data2009 by Mascot Daemon on SCT-14-65A4
MS data file : G:\PRESTATIONS\Labos académiques\Internationaux\Etats Unis\Martina Rosenberg\Data pour publi M Rosenberg\Identifications Mascot\MR_2944.mgf
Database : NCBIprot 20171205 (139,213,787 sequences; 51,013,024,959 residues)
Taxonomy : Rattus (77,467 sequences)
Timestamp : 25 Jan 2019 at 10:27:41 GMT

Not what you expected? Try **the peptide summary**.

Search parameters

Score distribution

Legend

Protein Family Summary

Significance threshold p<	0.05	Max. number of families	20
Display non-sig. matches	☐	Dendrograms cut at	0
Preferred taxonomy	All entries		

Sensitivity

Protein families 1–5 (out of 5)

10 per page 1

1

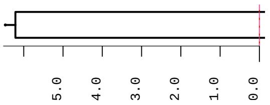

1	**NP_001008825.1**	67	keratin, type II cytoskeletal cochleal [Rattus norvegicus]
2	**EDL86882.1**	56	rCG50690, partial [Rattus norvegicus]

2 **NP_036767.1** 61 anionic trypsin-1 precursor [Rattus norvegicus]

3 **NP_034085.2** 59 dihydropyrimidinase-related protein 2 [Mus musculus]

		Score	Mass	Matches	Sequences	emPAI
3.1	NP_034085.2	59	62239	1 (1)	1 (1)	0.07

dihydropyrimidinase-related protein 2 [Mus musculus]

1 sameset of NP_034085.2

1 peptide matches (1 non-duplicate, 0 duplicate)

Query	Dupes	Observed	Mr(expt)	Mr(calc)	Delta	M	Score	Expect	Rank	U	Peptide
114		508.2400	1014.4654	1014.5458	-0.0803	0	59	0.0023	1	U	K.SAAEVIAQAR.K

4 **AAI28704.1** 39 Pgm1 protein, partial [Rattus norvegicus]

5 **XP_006247453.1** 36 PREDICTED: keratin, type I cytoskeletal 10 isoform X2 [Rattus norvegicus]

10 per page 1

Not what you expected? Try **the peptide summary**.

Mascot: http://www.matrixscience.com/

MATRIX SCIENCE MASCOT Search Results

Protein View: NP_034085.2

dihydropyrimidinase-related protein 2 [Mus musculus]

**Database:**	NCBIprot
**Score:**	59
**Monoisotopic mass (M_r):**	62239
**Calculated pI:**	5.95
**Taxonomy:**	Mus musculus

This protein sequence matches the following other entries:

- NP_001099187.1 from Rattus norvegicus
- XP_020031346.1 from Castor canadensis
- XP_021036957.1 from Mus caroli
- P47942.1 from Rattus norvegicus
- O08553.2 from Mus musculus
- CAA86981.1 from Rattus rattus
- AAH62955.1 from Mus musculus
- EDL35988.1 from Mus musculus
- EDL85394.1 from Rattus norvegicus

Sequence similarity is available as an NCBI BLAST search of NP_034085.2 against nr.

Search parameters

**MS data file:**	G:\PRESTATIONS\Labos académiques\Internationaux\Etats Unis\Martina Rosenberg\Data pour publi M Rosenberg\Identifications Mascot\MR_2944.mgf
**Enzyme:**	Trypsin: cuts C-term side of KR unless next residue is P.
**Variable modifications:**	Carbamidomethyl (C), Oxidation (M)

Protein sequence coverage: 1%

Matched peptides shown in ***bold red***.

```
  1 MSYQGKKNIP RITSDRLLIK GGKIVNDDQS FYADIYMEDG LIKQIGENLI
 51 VPGGVKTIEA HSRMVIPGGI DVHTRFQMPD QGMTSADDFF QGTKAALAGG
101 TTMIIDHVVP EPGTSLLAAF DQWREWADSK SCCDYSLHVD ITEWHKGIQE
151 EMEALVKDHG VNSFLVYMAF KDRFQLTDSQ IYEVLSVIRD IGAIAQVHAE
201 NGDIIAEEQQ RILDLGITGP EGHVLSRPEE VEAEAVNRSI TIANQTNCPL
251 YVTKVMSKSA AEVIAQARKK GTVVYGEPIT ASLGTDGSHY WSKNWAKAAA
301 FVTSPPLSPD PTTPDFLNSL LSCGDLQVTG SAHCTFNTAQ KAVGKDNFTL
351 IPEGTNGTEE RMSVIWDKAV VTGKMDENQF VAVTSTNAAK VFNLYPRKGR
401 ISVGSDADLV IWDPDSVKTI SAKTHNSALE YNIFEGMECR GSPLVVISQG
451 KIVLEDGTLH VTEGSGRYIP RKPFPDFVYK RIKARSRLAE LRGVPRGLYD
501 GPVCEVSVTP KTVTPASSAK TSPAKQQAPP VRNLHQSGFS LSGAQIDDNI
551 PRRTTQRIVA PPGGRANITS LG
```

Unformatted sequence string: 572 residues (for pasting into other applications).

Sort by ◉ residue number ○ increasing mass ○ decreasing mass
Show ◉ matched peptides only ○ predicted peptides also

Query	Start – End	Observed	Mr(expt)	Mr(calc)	Delta	M	Score	Expect	Rank	U	Peptide
114	259 – 268	508.2400	1014.4654	1014.5458	-0.0803	0	59	0.0023	1	U	K.SAAEVIAQAR.K

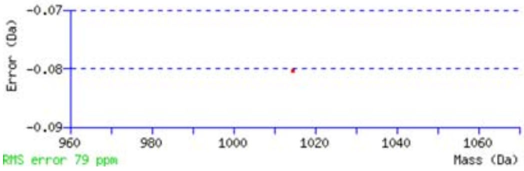

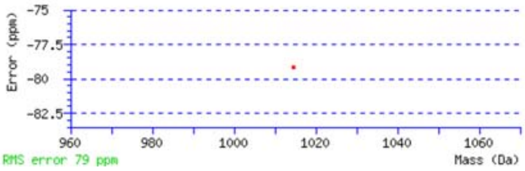


```
LOCUS       NP_034085                572 aa            linear   ROD 13-JAN-2019
DEFINITION  dihydropyrimidinase-related protein 2 [Mus musculus].
ACCESSION   NP_034085
VERSION     NP_034085.2
DBSOURCE    REFSEQ: accession NM_009955.3
KEYWORDS    RefSeq.
SOURCE      Mus musculus (house mouse)
  ORGANISM  Mus musculus
            Eukaryota; Metazoa; Chordata; Craniata; Vertebrata; Euteleostomi;
            Mammalia; Eutheria; Euarchontoglires; Glires; Rodentia; Myomorpha;
            Muroidea; Muridae; Murinae; Mus; Mus.
REFERENCE   1  (residues 1 to 572)
  AUTHORS   Abe H, Jitsuki S, Nakajima W, Murata Y, Jitsuki-Takahashi A,
            Katsuno Y, Tada H, Sano A, Suyama K, Mochizuki N, Komori T,
            Masuyama H, Okuda T, Goshima Y, Higo N and Takahashi T.
  TITLE     CRMP2-binding compound, edonerpic maleate, accelerates motor
            function recovery from brain damage
  JOURNAL   Science 360 (6384), 50-57 (2018)
   PUBMED   29622647
REFERENCE   2  (residues 1 to 572)
  AUTHORS   Dustrude ET, Perez-Miller S, Francois-Moutal L, Moutal A, Khanna M
            and Khanna R.
  TITLE     A single structurally conserved SUMOylation site in CRMP2 controls
            NaV1.7 function
  JOURNAL   Channels (Austin) 11 (4), 316-328 (2017)
   PUBMED   28277940
  REMARK    GeneRIF: postulate that this state forces CRMP2 toward a monomer,
            exposing the SUMO site and consequently, resulting in constitutive
            regulation of NaV1.7.
```

```
REFERENCE   3  (residues 1 to 572)
  AUTHORS   Sarhan AR, Szyroka J, Begum S, Tomlinson MG, Hotchin NA, Heath JK
            and Cunningham DL.
  TITLE     Quantitative Phosphoproteomics Reveals a Role for Collapsin
            Response Mediator Protein 2 in PDGF-Induced Cell Migration
  JOURNAL   Sci Rep 7 (1), 3970 (2017)
   PUBMED   28638064
  REMARK    GeneRIF: Results show that collapsin response mediator protein 2 is
            required for platelet derived growth factor-directed cell migration
            in vitro.
            Publication Status: Online-Only
REFERENCE   4  (residues 1 to 572)
  AUTHORS   Wang Y, Wang XL, Xie GL, Li HY and Wang YL.
  TITLE     Collapsin Response Mediator Protein-2-induced Retinal Ischemic
            Injury in a Novel Mice Model of Ocular Ischemia Syndrome
  JOURNAL   Chin. Med. J. 130 (11), 1342-1351 (2017)
   PUBMED   28524835
  REMARK    GeneRIF: These results revealed that bilateral ligation of the
            internal carotid artery causes retinal ischemia in mice. Moreover,
            CRMP2 might play a pivotal role during the ischemic injury in the
            retina and inhibit the cleavage of CRMP2 can ameliorate the IH
            injury.
REFERENCE   5  (residues 1 to 572)
  AUTHORS   Yamane M, Yamashita N, Hida T, Kamiya Y, Nakamura F, Kolattukudy P
            and Goshima Y.
  TITLE     A functional coupling between CRMP1 and Nav1.7 for retrograde
            propagation of Semaphorin3A signaling
  JOURNAL   J. Cell. Sci. 130 (8), 1393-1403 (2017)
   PUBMED   28254884
REFERENCE   6  (residues 1 to 572)
  AUTHORS   Fukada M, Watakabe I, Yuasa-Kawada J, Kawachi H, Kuroiwa A, Matsuda
            Y and Noda M.
  TITLE     Molecular characterization of CRMP5, a novel member of the
            collapsin response mediator protein family
  JOURNAL   J. Biol. Chem. 275 (48), 37957-37965 (2000)
   PUBMED   10956643
REFERENCE   7  (residues 1 to 572)
  AUTHORS   Inagaki H, Kato Y, Hamajima N, Nonaka M, Sasaki M and Eimoto T.
  TITLE     Differential expression of dihydropyrimidinase-related protein
            genes in developing and adult enteric nervous system
  JOURNAL   Histochem. Cell Biol. 113 (1), 37-41 (2000)
   PUBMED   10664068
REFERENCE   8  (residues 1 to 572)
  AUTHORS   Byk T, Ozon S and Sobel A.
  TITLE     The Ulip family phosphoproteins--common and specific properties
  JOURNAL   Eur. J. Biochem. 254 (1), 14-24 (1998)
   PUBMED   9652388
REFERENCE   9  (residues 1 to 572)
  AUTHORS   Kamata T, Subleski M, Hara Y, Yuhki N, Kung H, Copeland NG, Jenkins
            NA, Yoshimura T, Modi W and Copeland TD.
  TITLE     Isolation and characterization of a bovine neural specific protein
            (CRMP-2) cDNA homologous to unc-33, a C. elegans gene implicated in
            axonal outgrowth and guidance
  JOURNAL   Brain Res. Mol. Brain Res. 54 (2), 219-236 (1998)
   PUBMED   9555025
REFERENCE   10 (residues 1 to 572)
  AUTHORS   Hamajima N, Matsuda K, Sakata S, Tamaki N, Sasaki M and Nonaka M.
  TITLE     A novel gene family defined by human dihydropyrimidinase and three
            related proteins with differential tissue distribution
  JOURNAL   Gene 180 (1-2), 157-163 (1996)
   PUBMED   8973361
COMMENT     VALIDATED REFSEQ: This record has undergone validation or
            preliminary review. The reference sequence was derived from
            BC062955.1 and AC154693.2.
            On Dec 20, 2003 this sequence version replaced NP_034085.1.

            Publication Note:  This RefSeq record includes a subset of the
            publications that are available for this gene. Please see the Gene
            record to access additional publications.

            ##Evidence-Data-START##
            Transcript exon combination :: BC062955.1, X87242.1 [ECO:0000332]
            RNAseq introns              :: single sample supports all introns
                                           SAMN01164139, SAMN01164141
                                           [ECO:0000348]
            ##Evidence-Data-END##
FEATURES             Location/Qualifiers
     source          1..572
                     /organism="Mus musculus"
                     /strain="C57BL/6"
                     /db_xref="taxon:10090"
                     /chromosome="14"
                     /map="14 34.6 cM"
     Protein         1..572
                     /product="dihydropyrimidinase-related protein 2"
                     /note="DRP-2; ULIP-2; unc-33-like phosphoprotein 2;
                     collapsin response mediator protein 2"
                     /calculated_mol_wt=62147
     Region          17..466
                     /region_name="D-HYD"
                     /note="D-hydantoinases (D-HYD) also called
                     dihydropyrimidases (DHPase) and related proteins; DHPases
                     are a family of enzymes that catalyze the reversible
                     hydrolytic ring opening of the amide bond in five- or
                     six-membered cyclic diamides, like dihydropyrimidine...;
                     cd01314"
                     /db_xref="CDD:238639"
     Site            order(28..29,31,44..45,180,201,204,207,230,234,238,241,
                     268..269,271..272,390,395,400)
                     /site_type="other"
                     /note="tetramer interface [polypeptide binding]"
                     /db_xref="CDD:238639"
     Site            32
                     /site_type="other"
                     /experiment="experimental evidence, no additional details
                     recorded"
                     /note="Phosphotyrosine, by FYN.
                     {ECO:0000250|UniProtKB:Q16555}; propagated from
                     UniProtKB/Swiss-Prot (O08553.2)"
     Site            order(73,75,165,198,254,332)
                     /site_type="active"
                     /db_xref="CDD:238639"
     Site            258
```

```
                     /site_type="modified"
                     /experiment="experimental evidence, no additional details
                     recorded"
                     /note="N6-succinyllysine. {ECO:0000244|PubMed:23806337};
                     propagated from UniProtKB/Swiss-Prot (O08553.2)"
     Site            259
                     /site_type="other"
                     /experiment="experimental evidence, no additional details
                     recorded"
                     /note="Phosphoserine. {ECO:0000250|UniProtKB:P47942};
                     propagated from UniProtKB/Swiss-Prot (O08553.2)"
     Site            402
                     /site_type="other"
                     /experiment="experimental evidence, no additional details
                     recorded"
                     /note="Phosphoserine. {ECO:0000244|PubMed:21183079};
                     propagated from UniProtKB/Swiss-Prot (O08553.2)"
     Site            431
                     /site_type="other"
                     /experiment="experimental evidence, no additional details
                     recorded"
                     /note="Phosphotyrosine. {ECO:0000244|PubMed:18034455};
                     propagated from UniProtKB/Swiss-Prot (O08553.2)"
     Site            465
                     /site_type="other"
                     /experiment="experimental evidence, no additional details
                     recorded"
                     /note="Phosphoserine. {ECO:0000244|PubMed:15648052};
                     propagated from UniProtKB/Swiss-Prot (O08553.2)"
     Site            499
                     /site_type="other"
                     /experiment="experimental evidence, no additional details
                     recorded"
                     /note="Phosphotyrosine. {ECO:0000244|PubMed:18034455};
                     propagated from UniProtKB/Swiss-Prot (O08553.2)"
     Site            504
                     /site_type="nitrosylation"
                     /experiment="experimental evidence, no additional details
                     recorded"
                     /note="S-nitrosocysteine. {ECO:0000250|UniProtKB:P47942};
                     propagated from UniProtKB/Swiss-Prot (O08553.2)"
     Site            507
                     /site_type="other"
                     /experiment="experimental evidence, no additional details
                     recorded"
                     /note="Phosphoserine. {ECO:0000244|PubMed:21183079};
                     propagated from UniProtKB/Swiss-Prot (O08553.2)"
     Site            509
                     /site_type="other"
                     /experiment="experimental evidence, no additional details
                     recorded"
                     /note="Phosphothreonine. {ECO:0000244|PubMed:18034455,
                     ECO:0000244|PubMed:21183079}; propagated from
                     UniProtKB/Swiss-Prot (O08553.2)"
     Site            512
                     /site_type="other"
                     /experiment="experimental evidence, no additional details
                     recorded"
                     /note="Phosphothreonine. {ECO:0000244|PubMed:21183079};
                     propagated from UniProtKB/Swiss-Prot (O08553.2)"
     Site            514
                     /site_type="other"
                     /experiment="experimental evidence, no additional details
                     recorded"
                     /note="Phosphothreonine, by GSK3-beta.
                     {ECO:0000244|PubMed:19131326, ECO:0000244|PubMed:21183079,
                     ECO:0000269|PubMed:22057101}; propagated from
                     UniProtKB/Swiss-Prot (O08553.2)"
     Site            517
                     /site_type="other"
                     /experiment="experimental evidence, no additional details
                     recorded"
                     /note="Phosphoserine. {ECO:0000244|PubMed:19131326,
                     ECO:0000244|PubMed:21183079}; propagated from
                     UniProtKB/Swiss-Prot (O08553.2)"
     Site            518
                     /site_type="other"
                     /experiment="experimental evidence, no additional details
                     recorded"
                     /note="Phosphoserine. {ECO:0000244|PubMed:19131326,
                     ECO:0000244|PubMed:21183079}; propagated from
                     UniProtKB/Swiss-Prot (O08553.2)"
     Site            521
                     /site_type="other"
                     /experiment="experimental evidence, no additional details
                     recorded"
                     /note="Phosphothreonine. {ECO:0000244|PubMed:21183079};
                     propagated from UniProtKB/Swiss-Prot (O08553.2)"
     Site            522
                     /site_type="other"
                     /experiment="experimental evidence, no additional details
                     recorded"
                     /note="Phosphoserine. {ECO:0000244|PubMed:19131326,
                     ECO:0000244|PubMed:21183079}; propagated from
                     UniProtKB/Swiss-Prot (O08553.2)"
     Site            537
                     /site_type="other"
                     /experiment="experimental evidence, no additional details
                     recorded"
                     /note="Phosphoserine. {ECO:0000244|PubMed:21183079};
                     propagated from UniProtKB/Swiss-Prot (O08553.2)"
     Site            540
                     /site_type="other"
                     /experiment="experimental evidence, no additional details
                     recorded"
                     /note="Phosphoserine. {ECO:0000244|PubMed:21183079};
                     propagated from UniProtKB/Swiss-Prot (O08553.2)"
     Site            542
                     /site_type="other"
                     /experiment="experimental evidence, no additional details
                     recorded"
                     /note="Phosphoserine. {ECO:0000244|PubMed:21183079};
                     propagated from UniProtKB/Swiss-Prot (O08553.2)"
```

```
     Site            555
                     /site_type="other"
                     /experiment="experimental evidence, no additional details
                     recorded"
                     /note="Phosphothreonine, by ROCK2.
                     {ECO:0000250|UniProtKB:O02675}; propagated from
                     UniProtKB/Swiss-Prot (O08553.2)"
     Site            565
                     /site_type="other"
                     /experiment="experimental evidence, no additional details
                     recorded"
                     /note="Asymmetric dimethylarginine.
                     {ECO:0000244|PubMed:24129315}; propagated from
                     UniProtKB/Swiss-Prot (O08553.2)"
     CDS             1..572
                     /gene="Dpysl2"
                     /gene_synonym="AI851130; Crmp2; DRP2; Musunc33; TOAD-64;
                     Ulip2"
                     /coded_by="NM_009955.3:233..1951"
                     /db_xref="CCDS:CCDS27224.1"
                     /db_xref="GeneID:12934"
                     /db_xref="MGI:MGI:1349763"
```

Mascot: http://www.matrixscience.com/
