# Supplementary material for: Ethanol-Induced Alterations in Placental and Fetal Cerebrocortical Annexin-A4 and Cerebral Cavernous Malformation Protein 3 Are Associated With Reductions in Fetal Cortical VEGF Receptor Binding and Microvascular Density
Source: Front Neurosci. 2020 Jun 3;14:519. doi: 10.3389/fnins.2020.00519 (PMC7325964; doi:10.3389/fnins.2020.00519)
Supplement: Supplementary file 2 [file Table_1.DOCX]

**Supplementary Table 1**

Gene ontology analysis of the biological processes associated to the PAE-dysregulated placental proteins

| **Proteins** | **Gene** | **Angiogenesis** | **Brain development** | **Cell differentiation** | **Cell cycle/DNA synthesis** | **Cell metabolism** | **Cell survival** | **Intracellular transport** | **Structural** | **GO terms** |
| --- | --- | --- | --- | --- | --- | --- | --- | --- | --- | --- |
| Annexin A4 | ANXA4 | +  (Notch signaling) |  | + |  |  | +  (apoptosis) |  |  | GO:0007165 GO:0007219 |
| Alpha soluble NSF | NAPA |  | +  (synaptic transmission) | +  (neuronal differentiation) |  |  |  | + |  | GO:0007420 |
| Pyruvate dehydrogenase | PDHB |  |  |  |  | +  (energetic metabolism) |  |  |  | GO:0005975 GO:0006006 GO:0006086 |
| Dipeptidyl peptidase 3 | DPP3 |  |  |  |  | +  (proteolysis) |  |  |  | GO:0006508 |
| Cytoplasmic dynein 1 intermediate chain 2 | DYNC1/2 |  |  |  | +  (G2/M transition) |  |  | +  (vesicle motility) |  | GO:0000086  GO:0007018 |
| Apolipoprotein A-IV | APOA4 |  |  |  |  | +  (cholesterol) |  |  |  | GO:0006695 |
| Keratin 7 CRA_a | KRT7 |  |  |  |  |  |  |  | + (keratinization) | GO:0031424 |
| Heat shock protein 8 | HSPA8 |  |  |  | + | +  (ATP metabolism) | + (autophagy) |  |  | GO:0000398  GO:0006986  GO:0007269 |
| Heat shock protein 60 | HSPD1 |  |  |  |  |  | + (apoptosis) |  |  | GO:0002755  GO:0002842 |
| Protein disulfide isomerase 3 | PDIA3 |  |  |  |  |  | +  (apoptosis) |  |  | GO:0002474  GO:0006508 |
| EH domain containing protein 1 | EHD1 |  |  |  |  | + (cholesterol) |  | + |  | GO:0006886  GO:0010886 |
| Growth differentiation factor 7 | GDF7 |  | +  (axon guidance) | +  (neuronal differentiation) |  |  | + (apoptosis) |  |  | GO:0007411  GO:0021509  GO:0021527 |
| CCM3 | PDC10 | +  (VEGFR2, Notch signaling) | +  (CNS angiogenesis) |  |  |  | + (apoptosis) |  |  | GO:0001525  GO:0006915  GO:0008284 |
| Dihydropyrimidinase-related protein 2 | DPYSL2 |  | + |  |  |  |  |  |  | GO:0007010  GO:0007165 |
